# Supplementary material for: Whole-genome sequencing reveals sex determination and liver high-fat storage mechanisms of yellowstripe goby (Mugilogobius chulae)
Source: Commun Biol. 2021 Jan 4;4:15. doi: 10.1038/s42003-020-01541-9 (PMC7782490; doi:10.1038/s42003-020-01541-9)
Supplement: Supplementary file 2 — Supplementary Information [file 42003_2020_1541_MOESM2_ESM.pdf]

Whole-genome sequencing reveals sex determination and liver high-fat storage mechanisms of yellowstripe goby (*Mugilogobius chulae*)

Lei Cai[1 †*], Guocheng Liu[2†], Yuanzheng Wei[1†], Yabing Zhu[2†], Jianjun Li[1], Zongyu Miao[1], Meili Chen[1], Zhen Yue[2], Lujun Yu[1], Zhensheng Dong[2], Huixin Ye[1], Wenjing Sun[2], Ren Huang[1*]

[1]Guangdong Provincial Key Laboratory of Laboratory Animals, Guangdong Laboratory Animals Monitoring Institute, Guangzhou, China.
[2]BGI Genomics, BGI-Shenzhen, Shenzhen, China.
[†] These authors contributed equally to this manuscript.
*Correspondence and requests for materials should be addressed to L.C. (email: cailei17@163.com) or to R.H. (email: 1649405216@qq.com).

Supplementary information

Supplementary Table 1 Sequencing statistics

Sequencing Platform	Library Size	Average Read Length (bp)	Clean Reads (M)	Clean Base (Mb)	Sequencing Depth (X)
**Hiseq 4000**	270	150	405	60,725	60.7
	800	125	331	41,364	41.4
	20,000	49	321	15,716	15.7
	40,000	49	348	17,057	17.1
**RSII**	20,000	6,902	41.7	28,726	28.7

Supplementary Table 2 BUSCO evaluation

Type	Number	Percentage (%)
Complete BUSCOs (C)	3,989	87.0
Complete and single-copy BUSCOs (S)	3,856	84.1
Complete and duplicated BUSCOs (D)	133	2.9
Fragmented BUSCOs (F)	252	5.5
Missing BUSCOs (M)	343	7.5
Total BUSCO groups searched	4,584	100

Supplementary Table 3 Coverage evaluation

Samples	Dataset	Number	Total length(bp)	Covered by Assembly
**B1**	All	42331	3,193,1460	99.32
	>500	17310	24,385,099	99.86
	>1000	8703	18,357,350	99.91

**C1**	All	39109	24,949,295	99.46
	>500	13535	17,331,514	99.8
	>1000	6199	12,242,646	99.89

Supplementary Table 4 Protein-coding gene statistics

Gene number	20,531
Average gene length	24,832.78
Average CDS length	1,658
Average exon number per gene	9.01
Average exon length	183.99
Average intron length	2,892.76

Supplementary Table5 Gene functional annotation statistics

Type	Number	Percentage (%)
NR	19,608	95.50
NT	19,678	95.85
Swissprot	18,224	88.76
KEGG	17,072	83.15
COG	6,957	33.89
TrEMBL	19,541	95.18
Interpro	16,713	81.40
GO	11,665	56.82
Overall	19,729	96.09

Supplementary Table 6 Classification of repeat sequences

Type	Repbase TEs		TE protiens		De novo		Combined TEs	
	Length (Bp)	% in genome	Length (Bp)	% in genome	Length (Bp)	% in genome	Length (Bp)	% in genome
DNA	29305828	2.90	2134461	0.21	140212644	13.89	157634904	15.61
LINE	23732313	2.35	16751030	1.66	148415392	14.70	160353367	15.88
SINE	8526437	0.84	0	0.00	15644356	1.55	22821525	2.26
LTR	11082060	1.10	7657980	0.76	141869127	14.05	144909185	14.35
Other	13361	0.00	0	0.00	10496	0.00	22953	0.00
Unknown	0	0.00	0	0.00	27047698	2.68	27047698	2.68
Total	66868036	6.62	26540334	2.63	417808016	41.39	429658788	42.56

Supplementary Table 7 Significantly associated SNPs

SNP ID	Type(♀)	Type (♂)	SNP position
s247-672252	G	R	scaffold247:672252
s247-764174	G	R	scaffold247:764174
s247-842340	C	Y	scaffold247:842340

s247-842371	C	Y	scaffold247:842371
s247-849608	A	R	scaffold247:849608
s247-849806	G	R	scaffold247:849806
s247-888402	G	R	scaffold247:888402
s247-1213950	C	S	scaffold247:1213950
s247-1359738	A	M	scaffold247:1359738
s247-1831678	C	Y	scaffold247:1831678
s247-1921993	G	R	scaffold247:1921993
s247-1921995	G	R	scaffold247:1921995
s247-1946176	T	Y	scaffold247:1946176
s247-1982340	T	W	scaffold247:1982340
s247-1995608	C	Y	scaffold247:1995608
s247-2045083	G	R	scaffold247:2045083
s371-443786	A	W	scaffold371:443786
s371-645380	C	Y	scaffold371:645380
s572-209102	G	R	scaffold572:209102
s572-255894	C	Y	scaffold572:255894
s585-311970	G	S	scaffold585:311970
s585-338602	A	R	scaffold585:338602
s585-421493	A	M	scaffold585:421493
s585-453288	G	S	scaffold585:453288
s658-533324	G	R	scaffold658:533324

Supplementary Table 8 Genotype of locus numbered S247-888402

Sample ID	S247-888402	Sample ID	S247-888402	Sample ID	S247-888402
M1	G	M68	G	F35	G
M2	G	M69	G	F36	G
M3	G	M70	AG	F37	G
M4	G	M71	AG	F38	G
M5	G	M72	AG	F39	G
M6	G	M73	AG	F40	G
M7	G	M74	G	F41	G
M8	AG	M75	G	F42	G
M9	AG	M76	AG	F43	G
M10	AG	M77	G	F44	G
M11	G	M78	G	F45	G
M12	AG	M79	G	F46	G
M13	G	M80	AG	F47	G
M14	G	M81	G	F48	G
M15	AG	M82	G	F49	G
M16	G	M83	AG	F50	G
M17	AG	M84	G	F51	G
M18	G	M85	G	F52	G
M19	G	M86	G	F53	G
M20	AG	M87	G	F54	G
M21	G	M88	AG	F55	G
M22	AG	M89	G	F56	G
M23	G	M90	G	F57	G
M24	G	M91	AG	F58	G
M25	G	M92	G	F59	G
M26	G	M93	AG	F60	G

M27	AG	M94	G	F61	G
M28	G	M95	AG	F62	G
M29	AG	M96	G	F63	G
M30	AG	M97	G	F64	G
M31	G	M98	G	F65	G
M32	G	M99	G	F66	G
M33	A	M100	G	F67	G
M34	AG	F1	G	F68	G
M35	G	F2	G	F69	G
M36	G	F3	G	F70	G
M37	G	F4	G	F71	G
M38	AG	F5	G	F72	G
M39	G	F6	G	F73	G
M40	G	F7	G	F74	G
M41	G	F8	G	F75	G
M42	AG	F9	G	F76	G
M43	AG	F10	G	F77	G
M44	AG	F11	G	F78	G
M45	G	F12	G	F79	G
M46	AG	F13	G	F80	G
M47	AG	F14	G	F81	G
M48	G	F15	G	F82	G
M49	G	F16	G	F83	G
M50	AG	F17	G	F84	G
M51	AG	F18	G	F85	G
M52	G	F19	G	F86	G
M53	G	F20	G	F87	G
M54	AG	F21	G	F88	G
M55	AG	F22	G	F89	G
M56	G	F23	G	F90	G
M57	G	F24	G	F91	G
M58	G	F25	G	F92	G
M59	G	F26	G	F93	G
M60	AG	F27	G	F94	G
M61	AG	F28	G	F95	G
M62	G	F29	G	F96	G
M63	AG	F30	G	F97	G
M64	G	F31	G	F98	G
M65	G	F32	G	F99	G
M66	G	F33	G	F100	G
M67	G	F34	G	/	/

Supplementary Table 9 sex determination genes of *M. chulae*

Category	Gene ID	Gene name	FPKM* (SA)	FPKM (OV)
Sex determination region	Goby_GLEAN_10018952	MSL3	13.7	11.8
	Goby_GLEAN_10012546	H2AFY	27	15
	Goby_GLEAN_10018965	GALNT10	1.7	12.9
Female sex determination	Goby_GLEAN_10023745	FOXL2	0.27	8.39

Male sex determination	Goby_GLEAN_10028381	DMRT1	41	0
	Goby_GLEAN_10023987	GSDF	588	8.7
Female or male sex determination	Goby_GLEAN_10028465	FOXL3	6.59	0

FPKM: Fragments per Kilobase Million

Supplementary Table 10 Functional annotation of the 102 genes

Gene ID	Annotation
Goby_GLEAN_10018948	NA
Goby_GLEAN_10018949	PREDICTED: uncharacterized protein LOC102082692 [Oreochromis niloticus]
Goby_GLEAN_10018950	PREDICTED: FERM and PDZ domain-containing protein 4-like isoform X5 [Pundamilia nyererei]
Goby_GLEAN_10018951	unnamed protein product, partial [Tetraodon nigroviridis]
Goby_GLEAN_10018952	PREDICTED: male-specific lethal 3 homolog isoform X2 [Haplochromis burtoni]
Goby_GLEAN_10018953	PREDICTED: E3 ubiquitin
Goby_GLEAN_10018954	PREDICTED: trafficking kinesin-binding protein 2-like isoform X2 [Pundamilia nyererei]
Goby_GLEAN_10018955	hypothetical protein cypCar_00009947, partial [Cyprinus carpio]
Goby_GLEAN_10018956	PREDICTED: neurobeachin-like protein 1 [Larimichthys crocea]
Goby_GLEAN_10018957	PREDICTED: adrenodoxin, mitochondrial [Stegastes partitus]
Goby_GLEAN_10018958	PREDICTED: paraspeckle component 1 [Stegastes partitus]
Goby_GLEAN_10018959	hypothetical protein EGK_10984, partial [Macaca mulatta]
Goby_GLEAN_10018961	PREDICTED: methylmalonic aciduria and homocystinuria type D protein, mitochondrial [Larimichthys crocea]
Goby_GLEAN_10018964	NA
Goby_GLEAN_10018965	PREDICTED: polypeptide N-acetylgalactosaminyltransferase 10-like [Larimichthys crocea]
Goby_GLEAN_10018968	PREDICTED: microfibril-associated glycoprotein 3 [Stegastes partitus]
Goby_GLEAN_10018969	PREDICTED: protein FAM114A2 isoform X2 [Oreochromis niloticus]
Goby_GLEAN_10018970	PREDICTED: CCR4-NOT transcription complex subunit 8 [Stegastes partitus]
Goby_GLEAN_10018971	Histone H3c [Culex quinquefasciatus]
Goby_GLEAN_10018973	PREDICTED: histone H2B 1
Goby_GLEAN_10018975	PREDICTED: histone H4-like [Bombyx mori]
Goby_GLEAN_10018976	PREDICTED: histone H2B 1
Goby_GLEAN_10018977	PREDICTED: uncharacterized protein LOC101175291 [Oryzias latipes]
Goby_GLEAN_10018978	PREDICTED: uncharacterized protein LOC101175291 [Oryzias latipes]
Goby_GLEAN_10018980	PREDICTED: histone H2B 1
Goby_GLEAN_10018981	histone H4 [Culex quinquefasciatus]
Goby_GLEAN_10018982	PREDICTED: histone H4-like [Ursus maritimus]
Goby_GLEAN_10018984	histone H3.3 type 2 [Culex quinquefasciatus]
Goby_GLEAN_10018986	PREDICTED: solute carrier family 22 member 5-like [Maylandia zebra]
Goby_GLEAN_10018989	PREDICTED: histone H1-like [Xiphophorus maculatus]
Goby_GLEAN_10018990	Histone H3c [Culex quinquefasciatus]
Goby_GLEAN_10018991	PREDICTED: histone H2A-like [Cynoglossus semilaevis]
Goby_GLEAN_10018992	PREDICTED: histone H2B 1
Goby_GLEAN_10018996	core histone H2A
Goby_GLEAN_10018997	PREDICTED: histone H1-like [Xiphophorus maculatus]
Goby_GLEAN_10018998	PREDICTED: chloride intracellular channel protein 4 [Oryzias latipes]

Goby_GLEAN_10018999	PREDICTED: gem-associated protein 5 [Stegastes partitus]
Goby_GLEAN_10019000	PREDICTED: 39S ribosomal protein L22, mitochondrial isoform X1 [Takifugu rubripes]
Goby_GLEAN_10019001	Sterile alpha motif domain-containing protein 3 [Larimichthys crocea]
Goby_GLEAN_10019004	PREDICTED: coiled-coil domain-containing protein 106-like [Oryzias latipes]
Goby_GLEAN_10019005	NA
Goby_GLEAN_10019007	PREDICTED: uncharacterized protein LOC100701486 [Oreochromis niloticus]
Goby_GLEAN_10012532	NA
Goby_GLEAN_10012533	PREDICTED: thioredoxin domain-containing protein 15 [Fundulus heteroclitus]
Goby_GLEAN_10012534	PREDICTED: ubiquitin-conjugating enzyme E2 A-like [Neolamprologus brichardi]
Goby_GLEAN_10012535	PREDICTED: pterin-4-alpha-carbinolamine dehydratase 2-like isoform X1 [Oreochromis niloticus]
Goby_GLEAN_10012537	PREDICTED: pituitary homeobox 1 isoform X1 [Cynoglossus semilaevis]
Goby_GLEAN_10012538	NA
Goby_GLEAN_10012540	PREDICTED: putative nuclease HARBI1 [Fundulus heteroclitus]
Goby_GLEAN_10012543	NA
Goby_GLEAN_10012544	PREDICTED: homeodomain-interacting protein kinase 2-like [Poecilia formosa]
Goby_GLEAN_10012545	PREDICTED: homeodomain-interacting protein kinase 3-like isoform X2 [Poecilia mexicana]
Goby_GLEAN_10012546	PREDICTED: core histone macro-H2A.1 isoform X1
Goby_GLEAN_10012547	PREDICTED: fatty acid hydroxylase domain-containing protein 2 [Notothenia coriiceps]
Goby_GLEAN_10012548	PREDICTED: la-related protein 1B isoform X6 [Pan troglodytes]
Goby_GLEAN_10012550	PREDICTED: la-related protein 1 isoform X4 [Stegastes partitus]
Goby_GLEAN_10012552	PREDICTED: 15-hydroxyprostaglandin dehydrogenase [NAD(+)]-like [Stegastes partitus]
Goby_GLEAN_10012553	PREDICTED: beta-galactoside alpha-2,6-sialyltransferase 1-like [Stegastes partitus]
Goby_GLEAN_10012554	PREDICTED: neutrophil cytosol factor 1 [Austrofundulus limnaeus]
Goby_GLEAN_10012555	PREDICTED: heterogeneous nuclear ribonucleoprotein A
Goby_GLEAN_10012556	PREDICTED: maternal B9.10 protein-like [Larimichthys crocea]
Goby_GLEAN_10007973	PREDICTED: protein arginine N-methyltransferase 3-like [Neolamprologus brichardi]
Goby_GLEAN_10007974	PREDICTED: protein arginine N-methyltransferase 3 isoform X2 [Takifugu rubripes]
Goby_GLEAN_10007975	PREDICTED: oxidoreductase HTATIP2 [Larimichthys crocea]
Goby_GLEAN_10007980	PREDICTED: homeobox protein DBX1-B-like [Salmo salar]
Goby_GLEAN_10007981	PREDICTED: protein NLRC3-like [Maylandia zebra]
Goby_GLEAN_10007983	NA
Goby_GLEAN_10007984	PREDICTED: LOW QUALITY PROTEIN: uncharacterized protein LOC100198014 [Hydra vulgaris]
Goby_GLEAN_10007985	PREDICTED: rho GTPase-activating protein 7-like [Poecilia formosa]
Goby_GLEAN_10007986	PREDICTED: arginine--tRNA ligase, cytoplasmic [Austrofundulus limnaeus]
Goby_GLEAN_10007987	PREDICTED: ADP-ribosylation factor-like protein 9 [Notothenia coriiceps]
Goby_GLEAN_10007988	Nucleolar protein 16 [Larimichthys crocea]
Goby_GLEAN_10009650	NA

Goby_GLEAN_10009651	Polypeptide N-acetylgalactosaminyltransferase 10 [Larimichthys crocea]
Goby_GLEAN_10009656	PREDICTED: histone deacetylase complex subunit SAP30L isoform X1 [Xiphophorus maculatus]
Goby_GLEAN_10009657	PREDICTED: heart- and neural crest derivatives-expressed protein 1 [Takifugu rubripes]
Goby_GLEAN_10009658	PREDICTED: cornifelin homolog B-like isoform X2 [Stegastes partitus]
Goby_GLEAN_10009659	NA
Goby_GLEAN_10009660	Histone H3c [Culex quinquefasciatus]
Goby_GLEAN_10009661	histone H2A type 1-like [Scleropages formosus]
Goby_GLEAN_10009662	PREDICTED: histone H2B 1
Goby_GLEAN_10009663	PREDICTED: histone H2B 1
Goby_GLEAN_10009664	PREDICTED: histone H2A-like [Cynoglossus semilaevis]
Goby_GLEAN_10009665	Histone H3c [Culex quinquefasciatus]
Goby_GLEAN_10009666	PREDICTED: histone H1-like [Xiphophorus maculatus]
Goby_GLEAN_10009667	Histone H4 [Caprimulgus carolinensis]
Goby_GLEAN_10009668	NA
Goby_GLEAN_10009670	Histone H3c [Culex quinquefasciatus]
Goby_GLEAN_10009671	PREDICTED: histone H1-like [Xiphophorus maculatus]
Goby_GLEAN_10009672	PREDICTED: histone H4-like [Ursus maritimus]
Goby_GLEAN_10009673	PREDICTED: histone H2B 1
Goby_GLEAN_10009674	PREDICTED: solute carrier family 22 member 5-like isoform X2 [Oreochromis niloticus]
Goby_GLEAN_10009675	PREDICTED: PDZ and LIM domain protein 4 [Fundulus heteroclitus]
Goby_GLEAN_10009793	PREDICTED: brain-specific homeobox
Goby_GLEAN_10009797	PREDICTED: Krueppel-like factor 5 [Stegastes partitus]
Goby_GLEAN_10009798	NA
Goby_GLEAN_10009801	NA
Goby_GLEAN_10009802	PREDICTED: RNA-binding protein 41 isoform X1 [Oreochromis niloticus]
Goby_GLEAN_10009804	PREDICTED: claudin-9 isoform X2 [Maylandia zebra]
Goby_GLEAN_10009805	PREDICTED: transmembrane gamma-carboxyglutamic acid protein 3 isoform X1 [Larimichthys crocea]
Goby_GLEAN_10009806	PREDICTED: mitochondrial fission factor homolog B-like isoform X2 [Salmo salar]
Goby_GLEAN_10009808	PREDICTED: angiomotin-like isoform X3 [Stegastes partitus]

Supplementary Table 11 The lipid content in marine fishes

Species	Total lipids (% of livers)	References
Yellowstripe goby (*M.chulae*)	77	This study
European sea bass (*Dicentrarchus labrax*)	33.81	1
Cod (*Gadus morhua L.*)	38.30	2
Red sea bream （*Pagrus major*）	21.83	3
Amberjack （*Seriola dumerili*）	33.32	3
Striped jack （*Caranx delicatissimus* ）	53.26	3

Supplementary Table 12 Enriched KEGG pathways between G2M and G3M

Serial number	Pathway	DEGs	*P* value
1.	Metabolic pathways	857 (15.85%)	1.12E-39
2.	Oxidative phosphorylation	105 (1.94%)	1.18E-26
3.	Proteasome	52 (0.96%)	1.63E-18
4.	Carbon metabolism	104 (1.92%)	3.85E-12
5.	Non-alcoholic fatty liver disease (NAFLD)	113 (2.09%)	2.91E-10
6.	Glycerolipid metabolism	51 (0.94%)	2.24E-07
7.	Steroid biosynthesis	20 (0.37%)	1.82E-05
8.	Cholesterol metabolism	57 (1.05%)	2.53E-05
9.	Peroxisome	67 (1.24%)	2.84E-05
10.	Fatty acid metabolism	42 (0.78%)	4.00E-05
11.	Fat digestion and absorption	34 (0.63%)	1.37E-04
12.	Fatty acid elongation	23 (0.43%)	2.80E-03
13.	Glycerophospholipid metabolism	59 (1.09%)	5.02E-03
14.	Lysosome	81 (1.5%)	5.94E-03
15.	Biosynthesis of unsaturated fatty acids	22 (0.41%)	6.65E-03
16.	PPAR signaling pathway	60 (1.11%)	8.90E-03
17.	Fatty acid degradation	29 (0.54%)	1.02E-02
18.	Glycosylphosphatidylinositol (GPI)-anchor biosynthesis	14 (0.26%)	1.91E-02
19.	Arachidonic acid metabolism	35 (0.65%)	2.48E-02
20.	Linoleic acid metabolism	17 (0.31%)	2.90E-02

Supplementary Table 13 Key lipid metabolism genes expression between G2M and G3M group

Gene Name	FPKM								
	G2M-1	G2M-2	G2M-3	G2M-mean	Std	G3M-1	G3M-3	Goby3M- mean	Std
GPAT3	17.66	20.09	12.49	16.75	3.88	3.61	4.92	4.27	0.93
PAP1	4.85	6.56	2.88	4.76	1.84	1.17	2.36	1.77	0.84
DGAT2	274.02	346.09	117.14	245.75	117.06	24.20	57.13	40.67	23.29
LPCAT3	7.68	9.28	18.24	11.73	5.69	2.66	4.26	3.46	1.13
FDFT1	44.97	39.58	89.24	57.93	27.25	18.94	20.91	19.93	1.39
SQLE	24.18	20.98	114.81	53.32	53.27	9.17	13.03	11.10	2.73
LIPC	224.76	250.01	217.43	230.73	17.09	56.07	81.95	69.01	18.30
LDLR	54.60	49.80	38.22	47.54	8.42	13.91	22.39	18.15	6.00
MGLL	3.01	1.97	2.9	2.63	0.47	21.52	28.36	24.94	3.42
CPT1	43.73	21.95	7.02	24.23	18.46	14166.95	7189.37	10678.16	4933.89

Supplementary Table 14 The copy number of ABCA1 gene in different species[4, 5]

Species	ABCA1 number
Yellowstripe goby (*M. chulae*)	4
Bluespotted mudskipper (*Boleophthalmus pectinirostris*)	4
Channel catfish (*Ictalurus punctatus*)	3
Zebrafish (*Danio rerio*)	2
Japanese Medaka (*Oryzias latipes*)	3
Marine Medaka (*Oryzias melastigma*)	3
Japanese pufferfish (*Fugu rubripes*)	2

Freshwater pufferfish (*Tetraodon nigroviridis*)	2
Threespine stickleback (*Gasterosteus aculeatus*)	2
Nile tilapia (*Oreochromis niloticus*)	2
Atlantic cod (*Gadus morhua*)	2
Mexican cave tetra (*Astyanax mexicanus*)	2
Coelacanth (*Latmeria chalumnae*)	1
Mouse (*Mus musculus*)	1
Human (*Homo sapiens*)	1

Supplementary Table 15 The copy number of TLR23 gene in different species[6-8]

Species	TLR23 number
Yellowstripe goby (*M. chulae*)	15
Glacier lanternfish (*Benthosema glaciale*)	49
Round goby (*Neogobius melanostomus*)	40
European perch (*Perca fluviatilis*)	17
Kissing Gourami (*Helostoma temminkii*)	14
Bluespotted mudskipper (*Boleophthalmus pectinirostris*)	12
Minute mudskipper (*Periophthalmus magnuspinnatus*)	7
Giant mudskipper (*Periophthalmodon schlosseri*)	9
Walking goby (*Scartelaos histophorus*)	6
Mexican cave tetra (*Astyanax mexicanus*)	3
Amazon molly (*Poecilia formosa*)	3
Southern platyfish (*Xiphophorus maculatus*)	1
Nile tilapia (*Oreochromis niloticus*)	1
Japanese pufferfish (*Fugu rubripes*)	1
Freshwater pufferfish (*Tetraodon nigroviridis*)	1
Zebrafish (*Danio rerio*)	0
Medaka (*Oryzias latipes*)	0
coelacanth (*Latmeria chalumnae*)	0
Mouse (*Mus musculus*)	0
Human (*Homo sapiens*)	0

Supplementary Table 16 Number of TRIM family in seven species

Species	TRIM number
M. chulae	234
Danio rerio	240
Tetraodon nigroviridis	58[9]
Human	65[9]
Mice	64
Worm	~20
Flies	<10

Supplementary Table 17 Gene copy number of TRIM family

Gene name	Copy number	Gene name	Copy number
TRIM2	2	TRIM35	22
TRIM3	2	TRIM36	2
TRIM8	2	TRIM37	1
TRIM9	1	TRIM39	24
TRIM11	1	TRIM44	1
TRIM13	2	TRIM45	1
TRIM14	19	TRIM46	2

TRIM16	37	TRIM47	2
TRIM21	58	TRIM55	2
TRIM23	1	TRIM59	1
TRIM24	1	TRIM62	4
TRIM25	28	TRIM65	1
TRIM27	2	TRIM67	1
TRIM29	1	TRIM68	1
TRIM31	1	TRIM69	5
TRIM32	1	TRIM71	2
TRIM33	1	MID2	2
	Total		234

Supplementary Table 18 Individual ID for identifying sex determination region

Serial number	Parent ID			
	Female parent ID		Male parent ID	
1	WHYD17055725		WHYD17055726	
Serial number	Offspring ID			
	Female		Male	
1	WH1705002044	WHYD17055614	WHYD17055511	WHYD17055593
2	WHYD17055513	WHYD17055615	WHYD17055512	WHYD17055594
3	WHYD17055514	WHYD17055619	WH1705002047	WHYD17055595
4	WHYD17055516	WHYD17055620	WH1705002049	WHYD17055596
5	WHYD17055519	WHYD17055621	WH1705002050	WHYD17055597
6	WH1705002059	WHYD17055625	WH1705002051	WHYD17055605
7	WHYD17055521	WHYD17055627	WH1705002053	WHYD17055612
8	WHYD17055523	WHYD17055628	WHYD17055518	WHYD17055616
9	WHYD17055528	WHYD17055636	WHYD17055522	WHYD17055618
10	WHYD17055529	WHYD17055639	WHYD17055524	WHYD17055622
11	WHYD17055534	WHYD17055640	WHYD17055530	WHYD17055624
12	WHYD17055536	WHYD17055641	WHYD17055531	WHYD17055626
13	WHYD17055540	WHYD17055645	WHYD17055532	WHYD17055629
14	WHYD17055542	WHYD17055650	WHYD17055533	WHYD17055630
15	WH1705002089	WHYD17055661	WHYD17055536	WHYD17055637
16	WHYD17055548	WHYD17055667	WHYD17055538	WHYD17055642
17	WH1705002093	WHYD17055669	WHYD17055539	WHYD17055649
18	WH1705002094	WHYD17055673	WHYD17055543	WHYD17055657
19	WHYD17055551	WHYD17055676	WHYD17055544	WHYD17055662
20	WHYD17055552	WHYD17055680	WHYD17055545	WHYD17055665
21	WHYD17055553	WHYD17055685	WHYD17055547	WHYD17055668
22	WHYD17055555	WHYD17055688	WH1705002092	WHYD17055677
23	WHYD17055557	WHYD17055690	WHYD17055549	WHYD17055684
24	WHYD17055560	WHYD17055691	WHYD17055550	WHYD17055689
25	WHYD17055568	WH1705002240	WHYD17055554	WHYD17055699
26	WHYD17055571	WHYD17055693	WHYD17055558	WHYD17055707
27	WHYD17055574	WHYD17055695	WHYD17055559	WHYD17055709
28	WHYD17055577	WHYD17055698	WHYD17055561	WHYD17055714

29	WHYD17055583	WHYD17055702	WHYD17055562	WHYD17055717
30	WHYD17055586	WHYD17055703	WHYD17055566	WHYD17055718
31	WHYD17055592	WHYD17055705	WHYD17055570	WHYD17055722
32	WHYD17055600	WHYD17055710	WHYD17055580	/
33	WHYD17055601	WHYD17055711	WHYD17055584	/
34	WHYD17055603	WHYD17055715	WHYD17055587	/
35	WHYD17055610	WHYD17055716	WHYD17055589	/
36	WHYD17055613	WHYD17055721	WHYD17055591	/

Supplementary figure legends

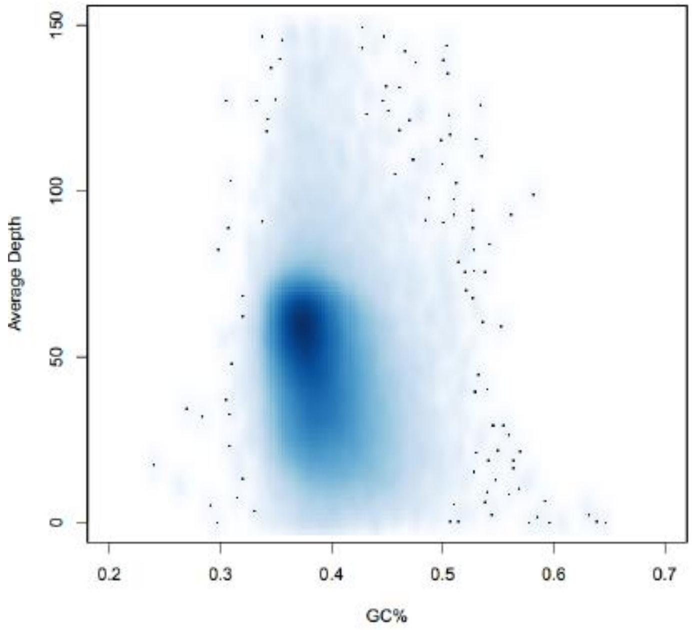

Supplementary Fig. 1 Relationship between GC content (horizontal axis) and sequencing depth (vertical axis) in a 10-kb window.

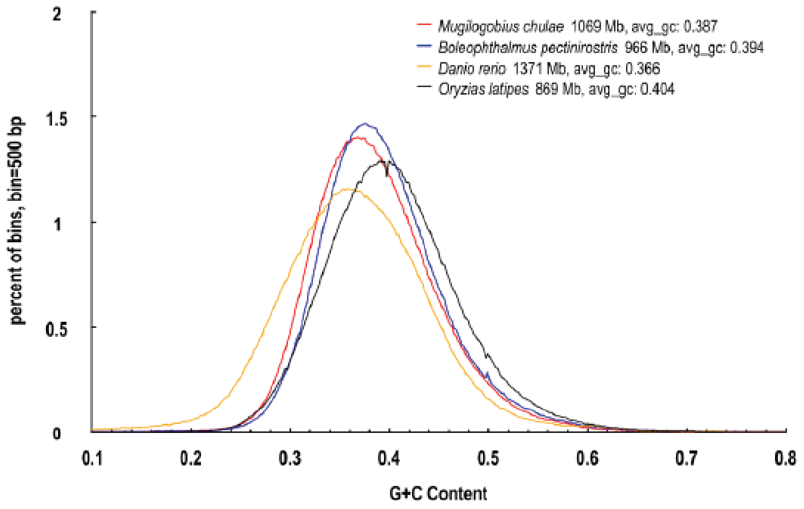

Supplementary Fig. 2 GC-content distributions for the genomes of the indicated species. The horizontal axis shows the GC content and the vertical axis shows the proportion of bin numbers in the total number of windows. We used 500-bp bins (with a 250-bp overlap) sliding along the genome. Using this graph, we could compare the GC-content distributions of related species. In general, related species had similar distribution curves.

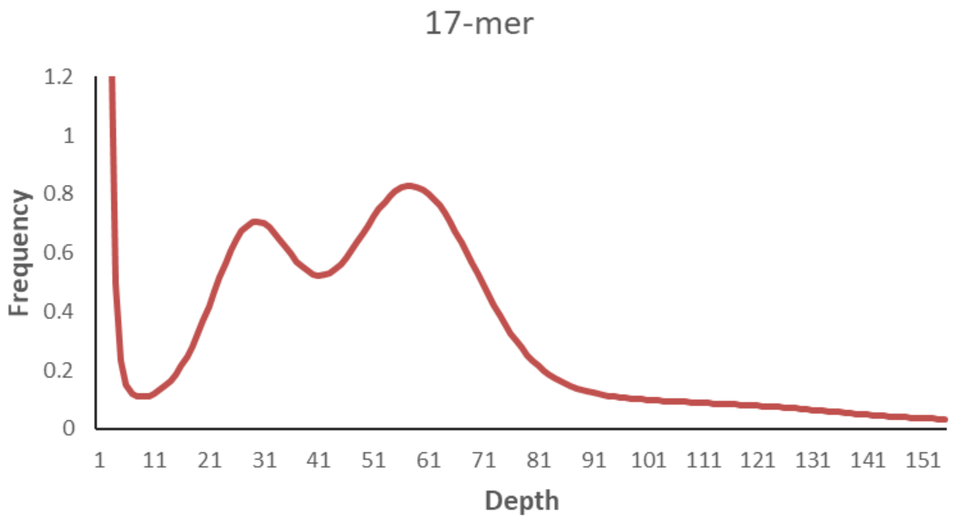

Supplementary Fig. 3 Genome size estimation（k-mer=17)

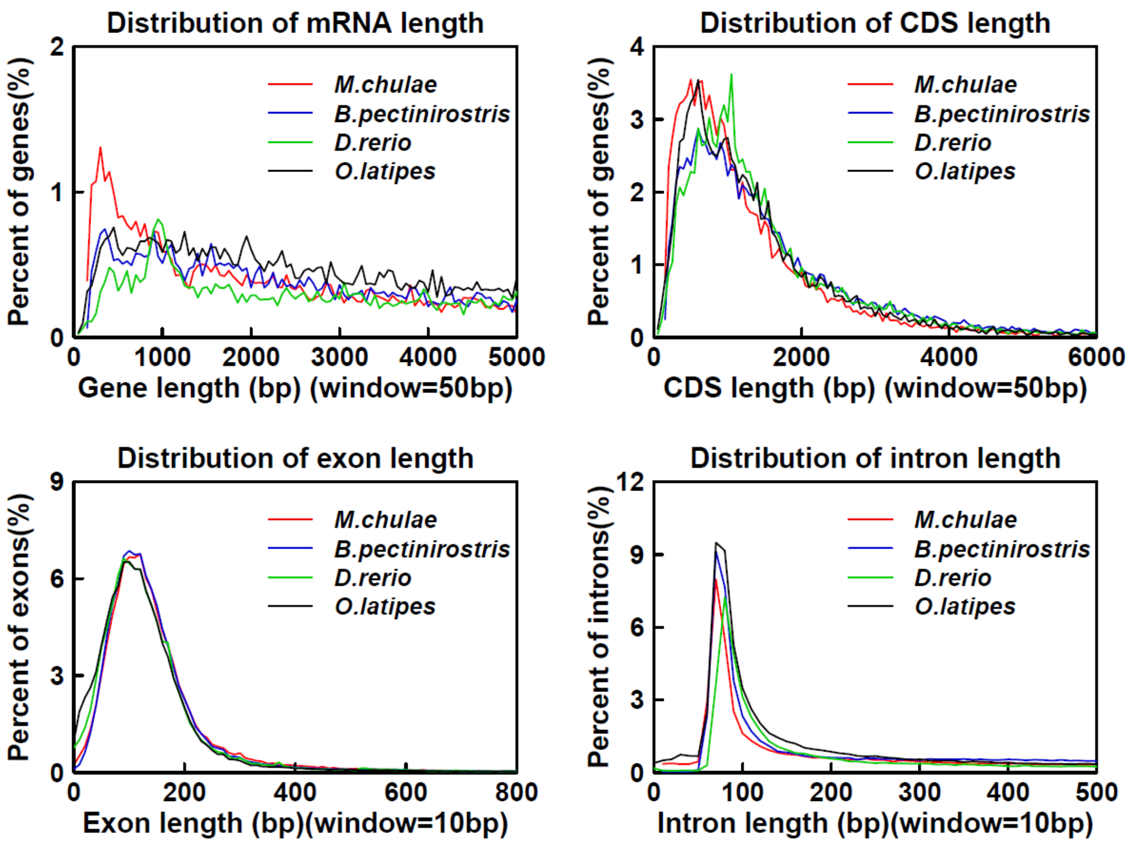

Supplementary Fig. 4 Comparison of gene-prediction between four fishes

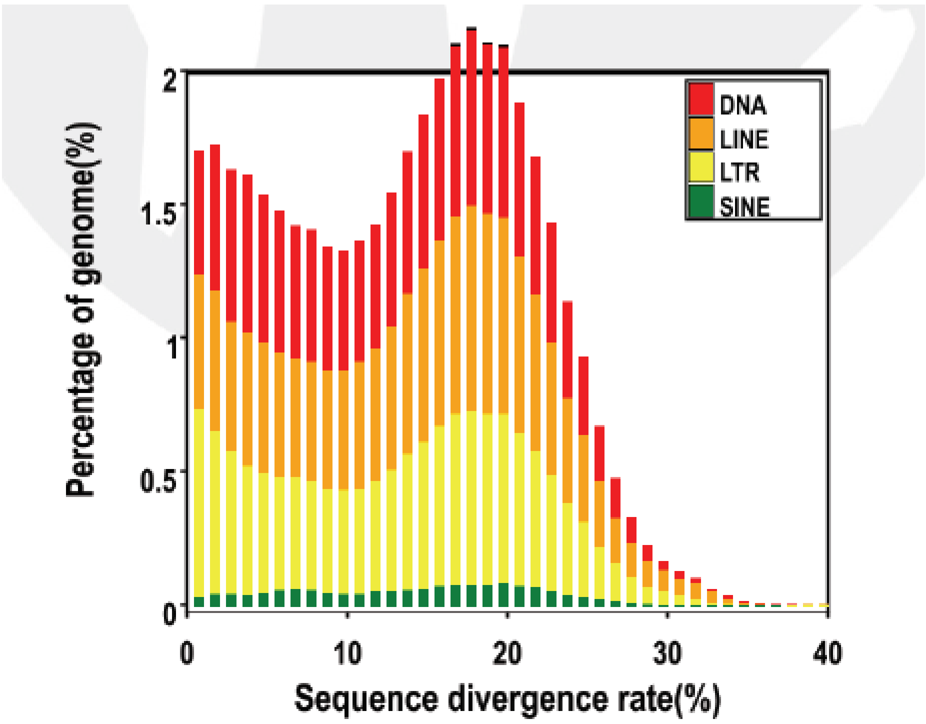

Supplementary Fig. 5 Divergence rates of four types of repeat sequences

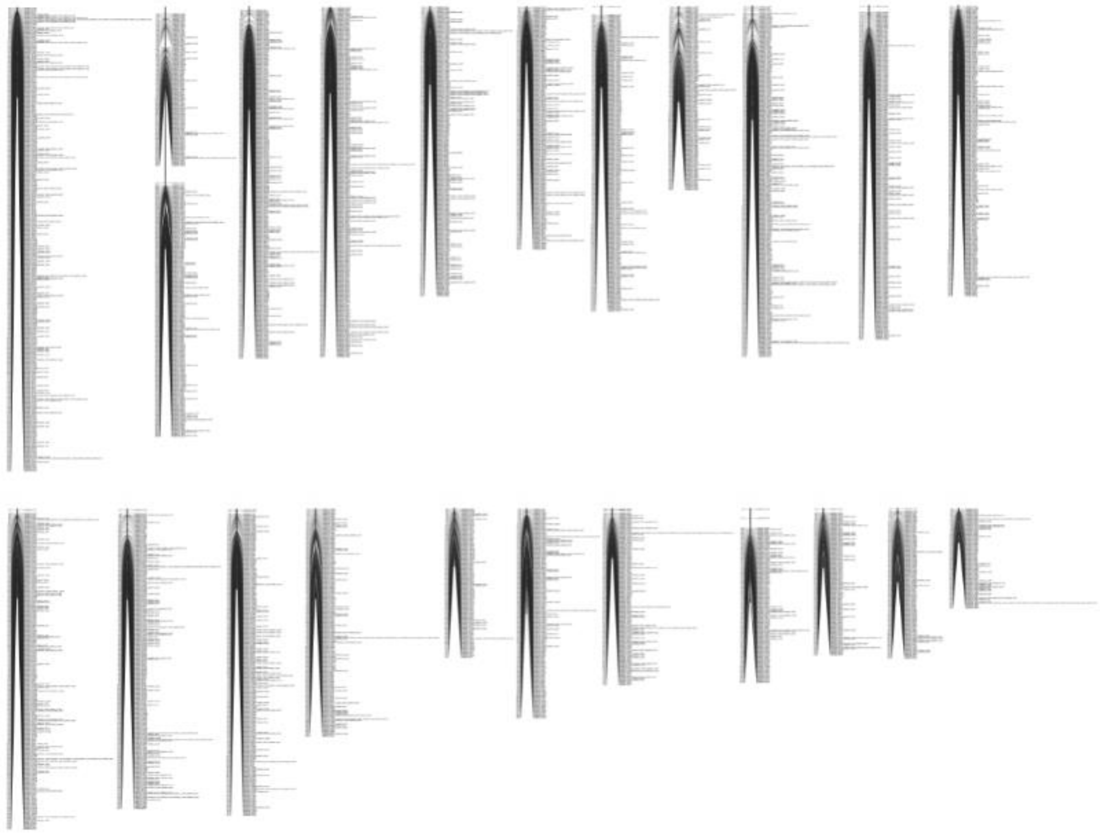

Supplementary Fig. 6 High-density SNP-based genetic linkage map of yellowstripe goby

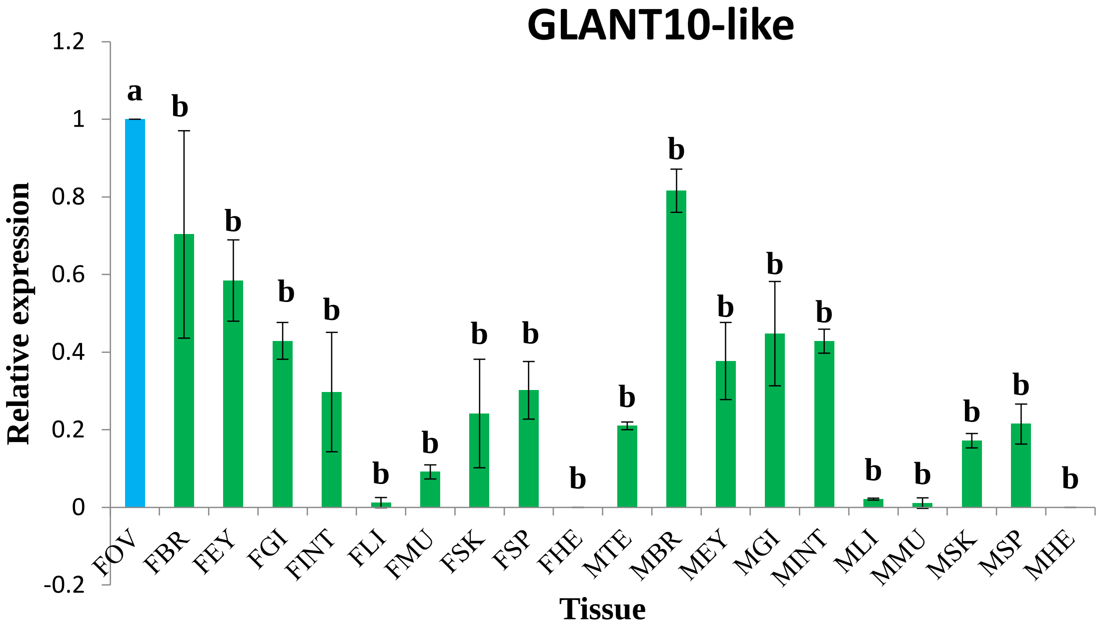

Supplementary Fig. 7a Quantitative real-time PCR validation of *GLANT10-like* in different tissues of female and male individuals. Means not sharing a common letter are significantly different at $p < 0.05$, as assessed using one-way ANOVA followed by Dunnett's test. (FOV~FHE: female fish, MTE~MHE: male fish. OV: ovary, TE: testis, BR: brain, EY: eye, GI: gill, INT: intestine, LI: liver, MU: muscle, SK: skin, SP: spleen, HE: heart.)

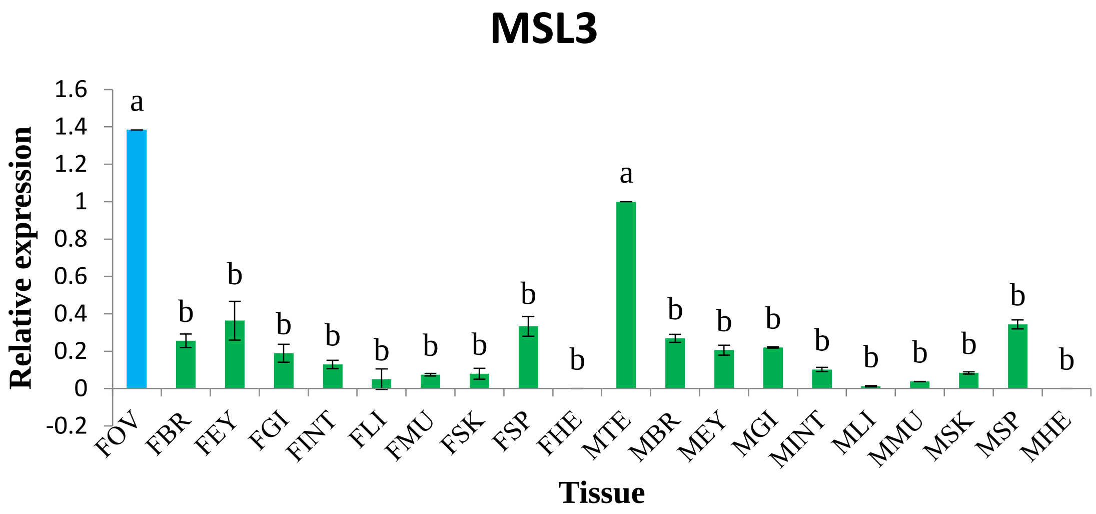

Supplementary Fig. 7b Quantitative real-time PCR validation of *MSL3* in different tissues of female and male individuals. Means not sharing a common letter are significantly different at $p < 0.05$, as assessed using one-way ANOVA followed by Dunnett's test. (FOV~FHE: female fish, MTE~MHE: male fish. OV: ovary, TE: testis, BR: brain, EY: eye, GI: gill, INT: intestine, LI: liver, MU: muscle, SK: skin, SP: spleen, HE: heart.)

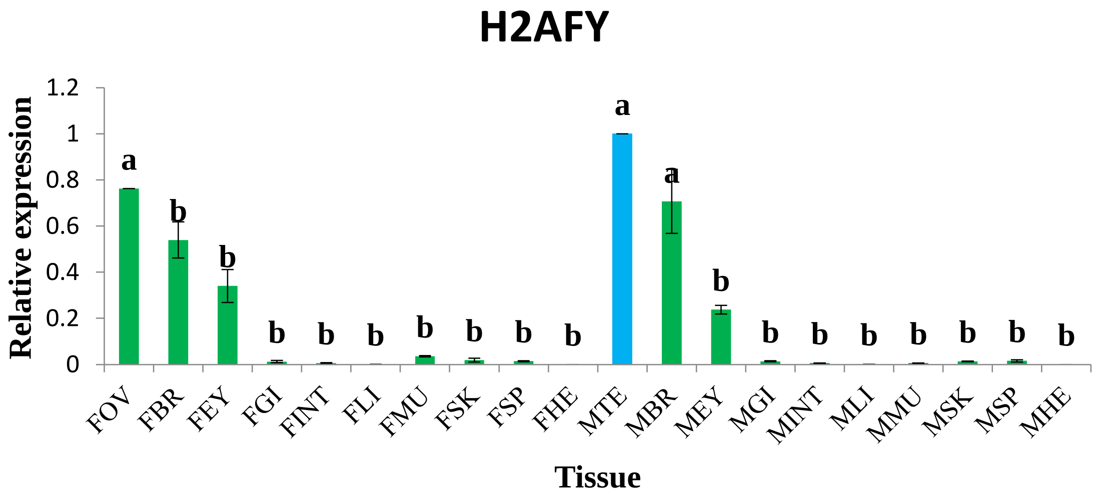

Supplementary Fig. 7c Quantitative real-time PCR validation of *H2AFY* in different tissues of female and male individuals. Means not sharing a common letter are significantly different at $p < 0.05$, as assessed using one-way ANOVA followed by Dunnett's test. (FOV~FHE: female fish, MTE~MHE: male fish. OV: ovary, TE: testis, BR: brain, EY: eye, GI: gill, INT: intestine, LI: liver, MU: muscle, SK: skin, SP: spleen, HE: heart.)

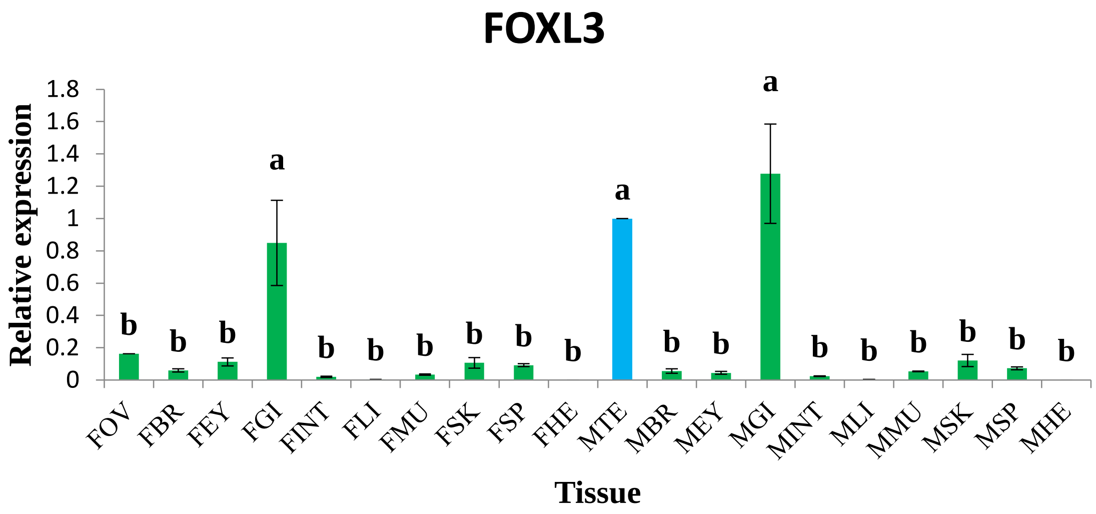

Supplementary Fig. 7d Quantitative real-time PCR validation of *FOXL3* in different tissues of female and male individuals. Means not sharing a common letter are significantly different at $p < 0.05$, as assessed using one-way ANOVA followed by Dunnett's test. (FOV~FHE: female fish, MTE~MHE: male fish. OV: ovary, TE: testis, BR: brain, EY: eye, GI: gill, INT: intestine, LI: liver, MU: muscle, SK: skin, SP: spleen, HE: heart.)

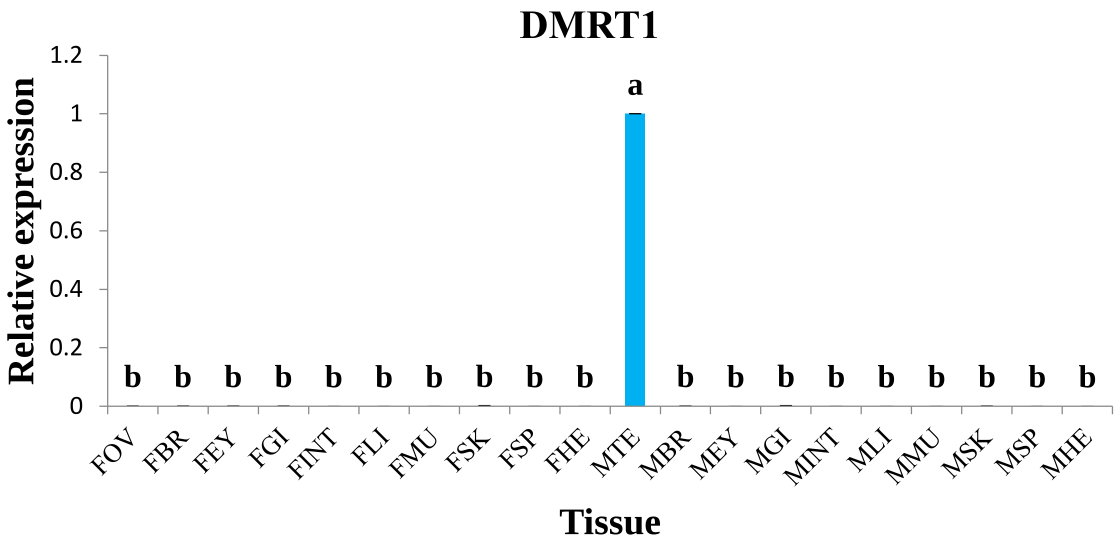

Supplementary Fig. 7e Quantitative real-time PCR validation of *DMRT1* in different tissues of female and male individuals. Means not sharing a common letter are significantly different at $p < 0.05$, as assessed using one-way ANOVA followed by Dunnett's test. (FOV~FHE: female fish, MTE~MHE: male fish. OV: ovary, TE: testis, BR: brain, EY: eye, GI: gill, INT: intestine, LI: liver, MU: muscle, SK: skin, SP: spleen, HE: heart.)

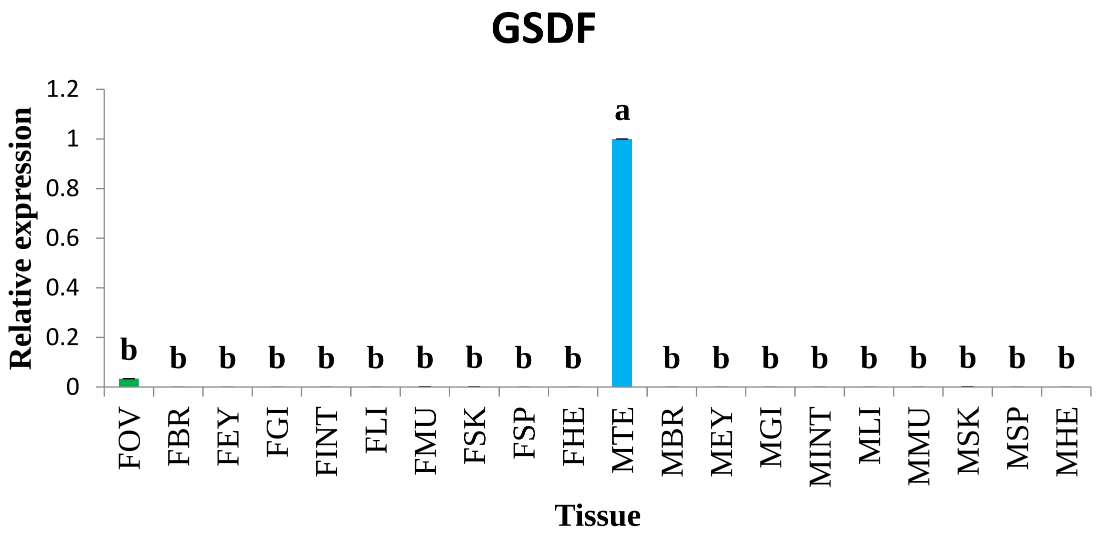

Supplementary Fig. 7f Quantitative real-time PCR validation of *GSDF* in different tissues of female and male individuals. Means not sharing a common letter are significantly different at $p < 0.05$, as assessed using one-way ANOVA followed by Dunnett's test. (FOV~FHE: female fish, MTE~MHE: male fish. OV: ovary, TE: testis, BR: brain, EY: eye, GI: gill, INT: intestine, LI: liver, MU: muscle, SK: skin, SP: spleen, HE: heart.)

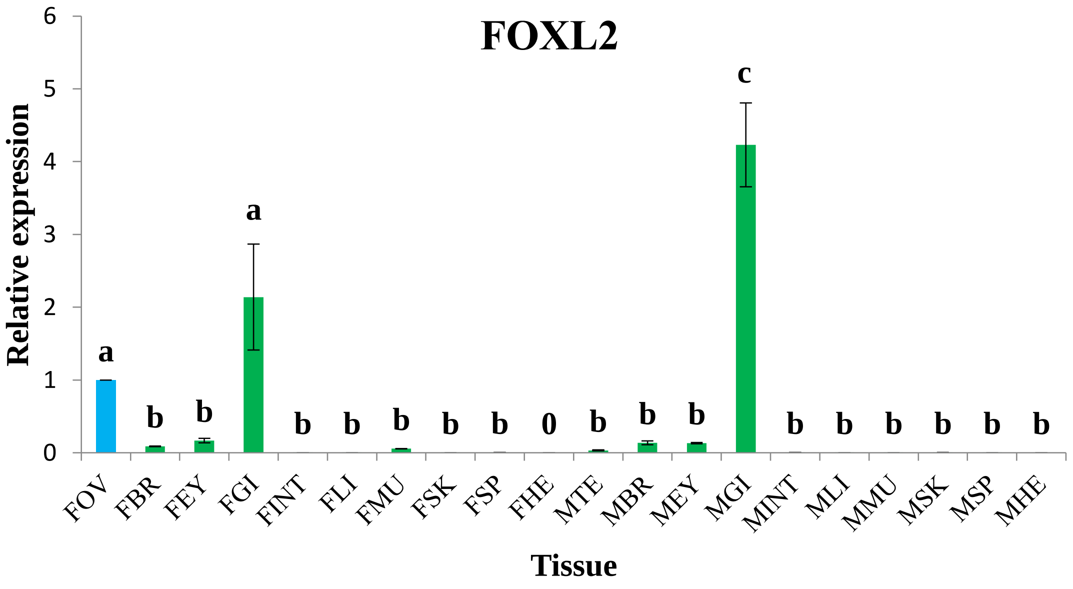

Supplementary Fig. 7g Quantitative real-time PCR validation of *FOXL2* in different tissues of female and male individuals. Means not sharing a common letter are significantly different at $p < 0.05$, as assessed using one-way ANOVA followed by Dunnett's test. (FOV~FHE: female fish, MTE~MHE: male fish. OV: ovary, TE: testis, BR: brain, EY: eye, GI: gill, INT: intestine, LI: liver, MU: muscle, SK: skin, SP: spleen, HE: heart.)

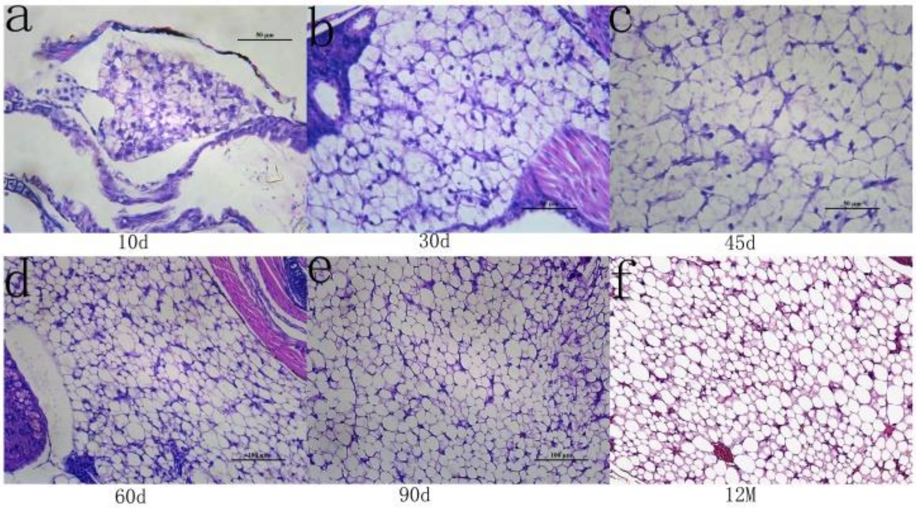

Supplementary Fig. 8 Histological observation of the liver of yellowstripe goby. a Histological observation of the liver in10 dah (day after hatching) fish by HE staning. **b** Histological observation of the liver in 30 dah fish by HE staning. **c** Histological observation of the liver in 45 dah fish by HE staning. **d** Histological observation of the liver in 60 dah fish by HE staining. **e** Histological observation of the liver in 90 dah fish by HE staining. **f** Histological observation of the liver in twelve months old fish by HE staining.

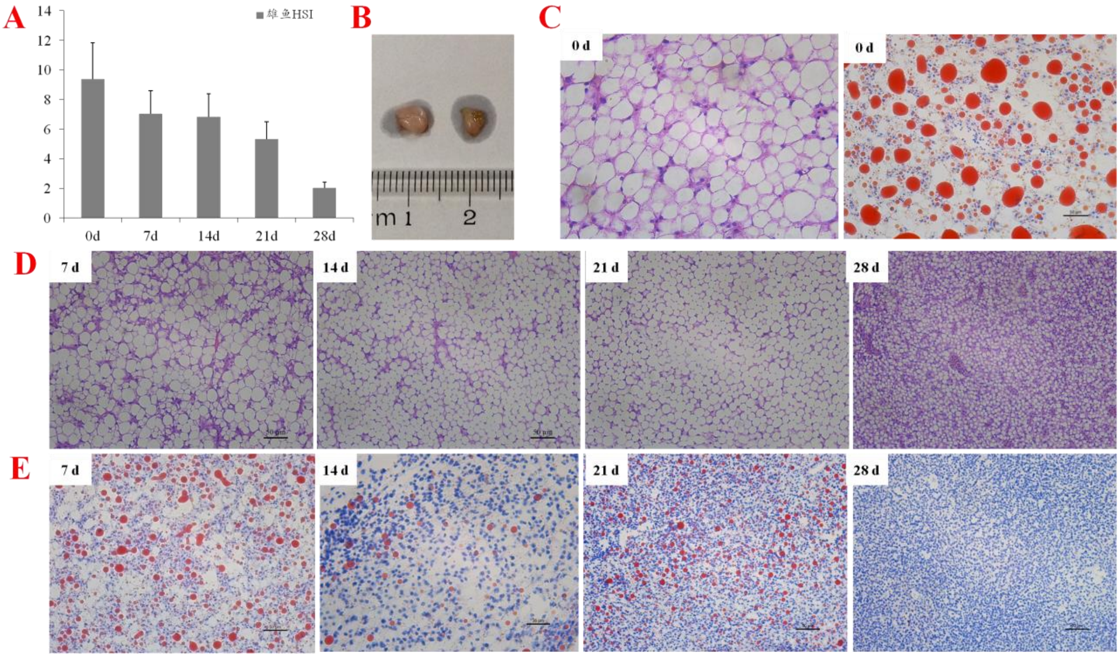

Supplementary Fig. 9 Histological observation of the liver of yellowstripe goby during 28-day starvation assay.

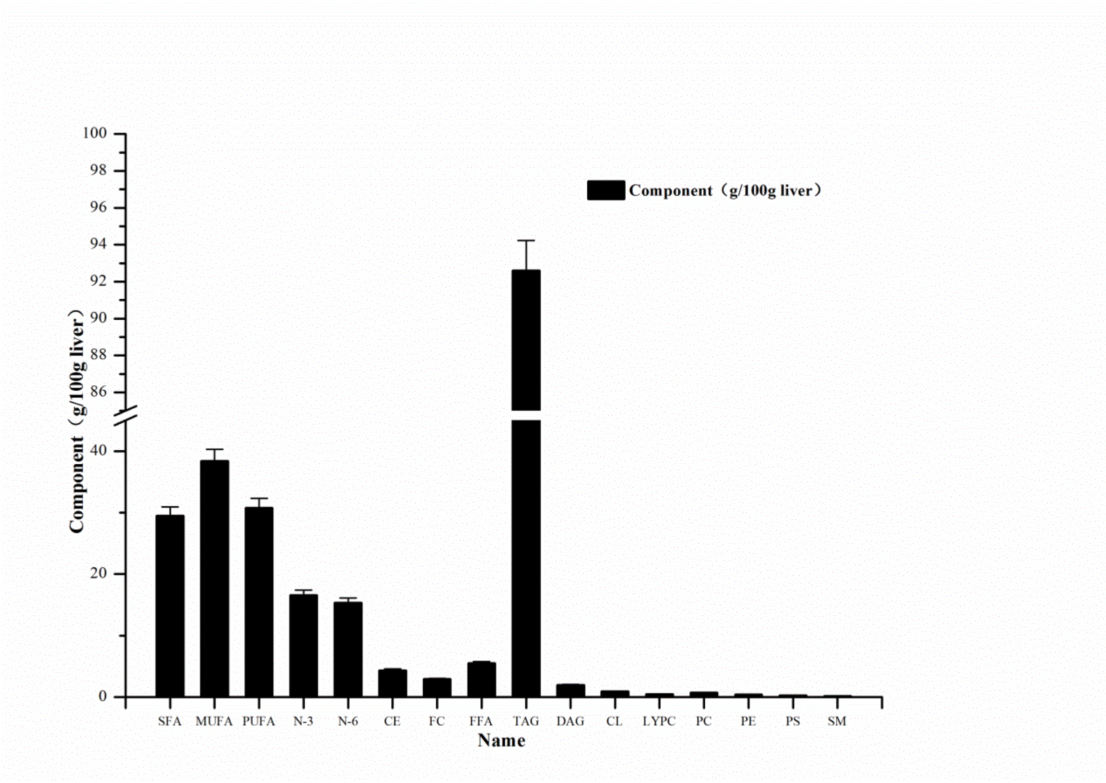

Supplementary Fig. 10 Lipid component of the liver of yellowstripe goby. Abbreviations: CE: cholesterol ester, CL: diphosphatidylglycerol, DAG: diacylglycerol, FC: free cholesterol, FFA: free fatty acid, LYPC: lysophosphatidyl choline, MUFA: mono-unsaturated fatty acid, N-3: 3-poly-unsaturated fatty acid, N-6: 6-poly-unsaturated fatty acid, PC: phosphatidyl choline (lecithin), PE: phosphatidylethanolamine (cephalin), PS: phosphatidylserine, PUFA: poly-unsaturated fatty acid, SFA: saturated fatty acid, SM: sphingomyelin, TAG: triglyceride

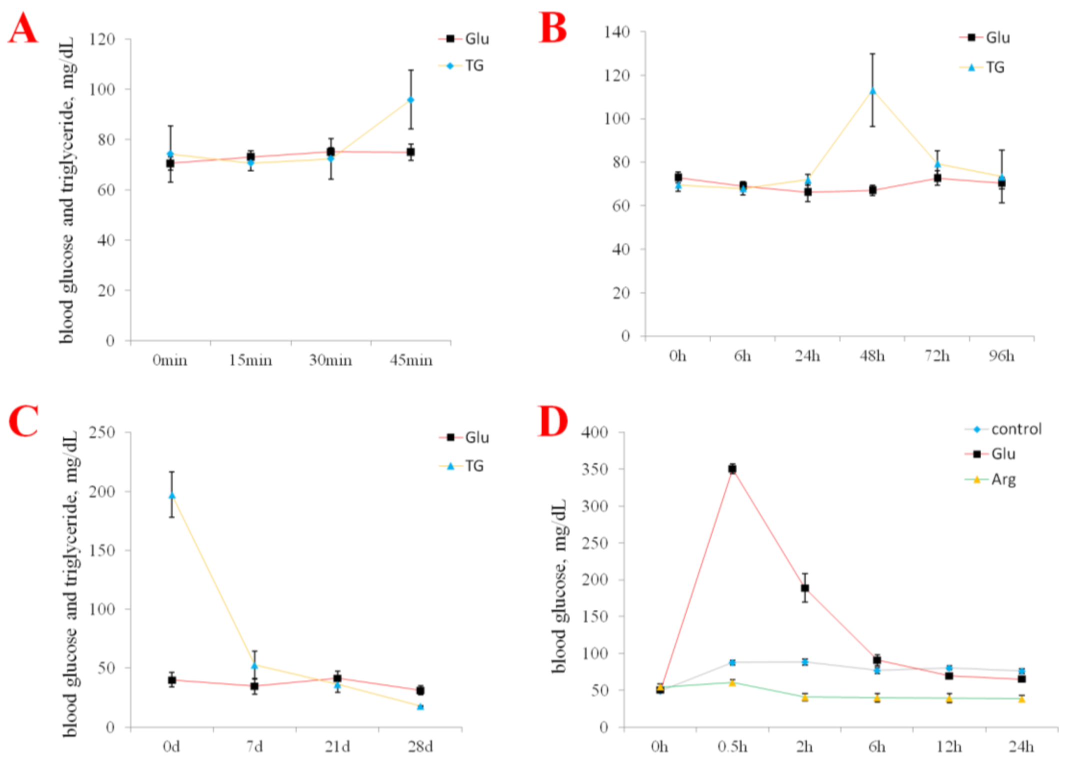

Supplementary Fig. 11 Blood glucose content of yellowstripe goby. a Effects of short-term fasting on TAG and blood glucose contents in yellowstripe goby. **b** Effects of medium and long-term fasting on TAG and blood-glucose contents in yellowstripe goby. **c** Effects of long-term fasting on TAG and blood-glucose contents in yellowstripe goby. **d** Effects of injecting Cortland solution (control), glucose, and arginine on TAG and blood glucose contents in yellowstripe goby.

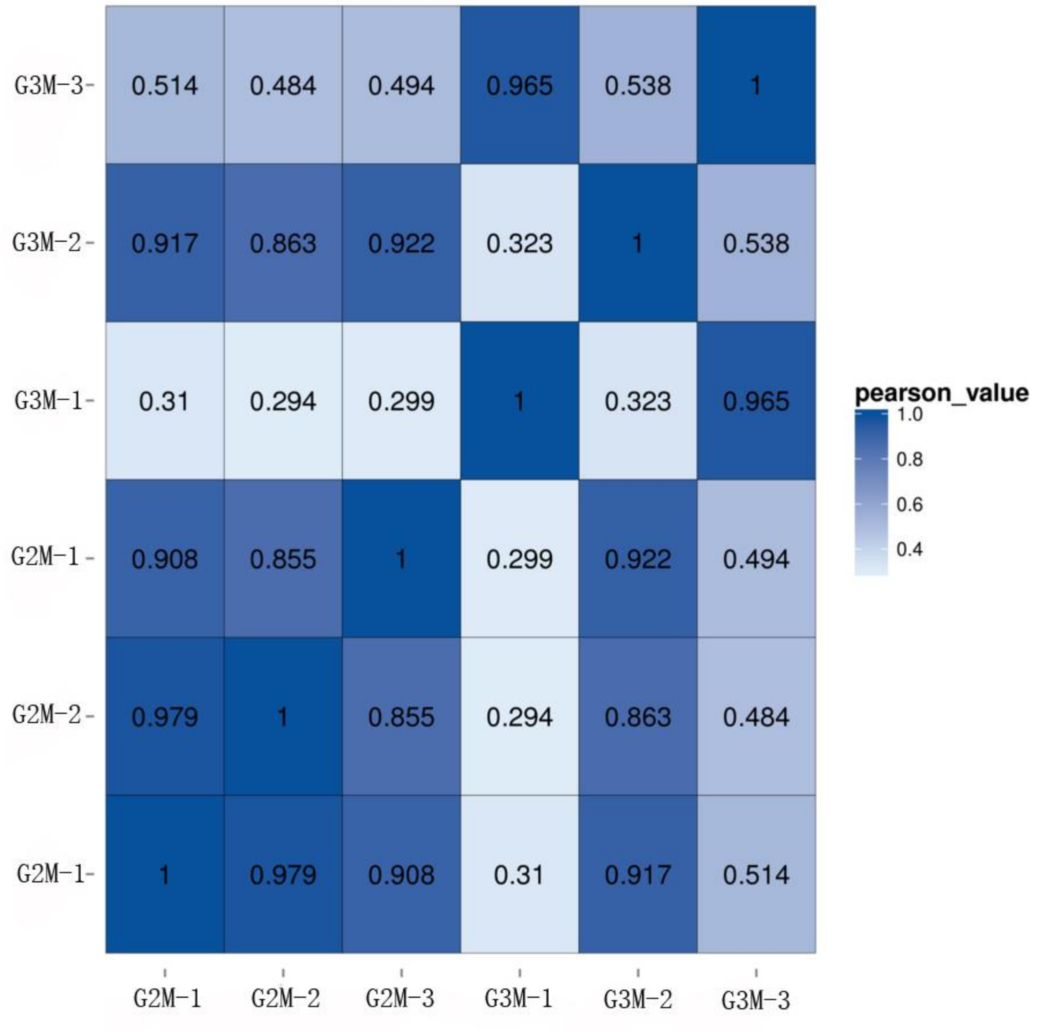

Supplementary Fig. 12 Correlation heatmap of the G2M group and G3M group

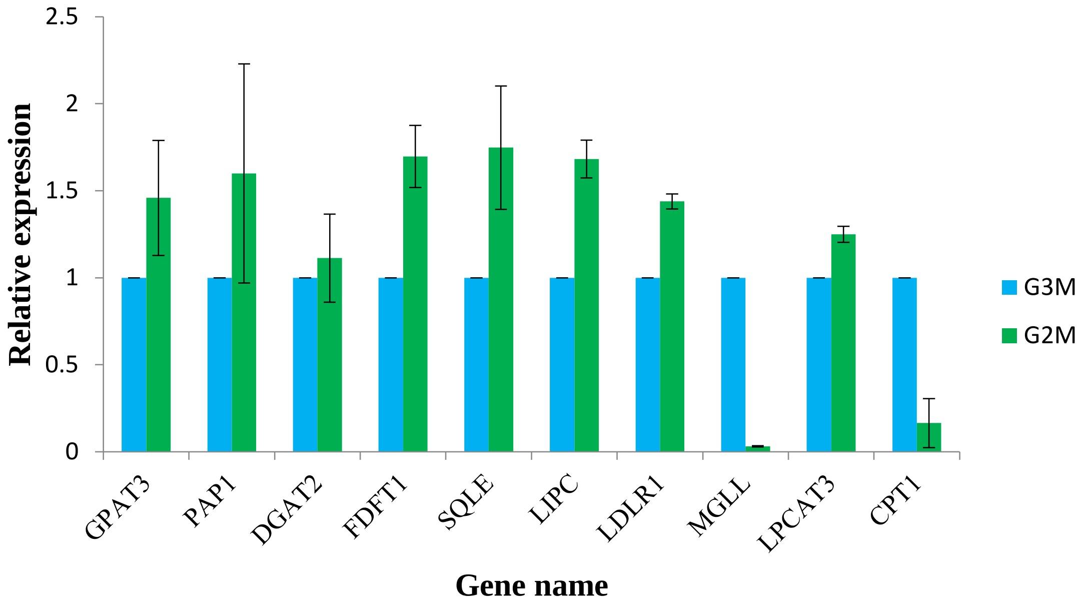

Supplementary Fig. 13 Quantitative real-time PCR validation of ten key lipid metabolism genes between G2M and G3M group. Concordance between RNA-seq and qPCR results was observed for all ten genes.

Supplementary Methods

SNP typing

Test samples

All experimental operations were performed according to the standard welfare regulations for laboratory fish and were approved by the Animal Care and Use Committee of Guangdong Laboratory Animals Monitoring Institute (Guangzhou, China). The samples used for SNP genotyping ($n = 200$, half male and half female) were from a wild population purchased from Panyu District Market (Guangzhou City, Guangdong Province), which were morphologically identified as yellowstripe goby.

SNP genotyping

The samples were sent to Shanghai Generay Biotech Co., Ltd., and SNP genotyping was carried out using the Snapshot method, as described previously[10].

Transcriptome sequencing

Materials

The samples used for sex-transcriptome analysis were 12-month-old closed groups of yellowstripe goby bred in the laboratory, comprising nine males and nine females. The tissues of every three fishes of the same group were pooled into one sample, for a total of twenty samples (ten from males and ten from females). The samples from females were labeled as FOV (ovary), FBR (brain), FEY (eye), FGI (gill), FINT (intestine), FLI (liver), FMU (muscle), FSK (skin), FSP (spleen), and FHE (heart), while the samples from males were labeled as MSA (testis), MBR (brain), MEY (eye), MGI (gill), MINT (intestine), MLI (liver), MMU (muscle), MSK (skin), MSP (spleen), and MHE (heart). FOV and MSA were used for transcriptome sequencing. The remaining samples were used for quantitative real-time PCR Validation

The samples used for liver fat-accumulation transcriptome analysis were from a closed colony of 2-month-old and 3-month-old yellowstripe goby, with 18 and 9 individuals, respectively. The fish were bred in our laboratory. The livers of three 2-month-old and three 3-month-old fishes were pooled into two samples each, to give a total of six samples (three duplicates in each group). The 2-month-old sample was labeled G2M (duplicate samples: G2M-1, G2M-2 and G2M-3), and the 3-month-old sample was labeled G3M (duplicate samples: G3M-1, G3M-2 and G3M-3).

Sequencing

The transcriptomes of the above samples were sequenced using the Illumina sequencing platform.

Data analysis and processing

The data were analyzed and processed mainly based on previous research[11]. Raw reads were processed by removing the adapters along with low-quality sequences. All unigenes were

aligned to the Nr, Nt, Swissprot, Kyoto Encyclopedia of Genes and Genomes (KEGG), and Gene Ontology (GO) databases with the Basic Local Alignment Search Tool program. A robust and efficient stack memory management method was used to count the reads mapped to each unigene. The reads per kilobase per million mapped reads (RPKM) value was measured based on transcript length and read counts mapped to the transcript. Differentially expressed genes were detected with the DEGSeq R package.

Glucose-tolerance test

Experimental fish

The experimental fish were selected from a 200-day-old closed group bred in our laboratory, totaling 180 individuals.

Assays

The experimental fish were randomly divided into six groups, and three individuals in each group were used in repeated glucose-tolerance experiments. In brief, the fish were subjected to fasting for 24 h, followed by ice-water bath anesthesia. Next, the fish were weighed and injected intraperitoneally with glucose diluted in Cortland's salt solution (1 mg/g body weight) and arginine diluted in Cortland's salt solution (2.5 μg/g body weight). The control group received the same amount of Cortland's salt solution. Blood samples were collected at 0, 0.5, 2, 6, 12, and 24 h after intraperitoneal injection, as follows. First, the adult fish were placed in an ice-water bath for anesthesia, after which the water on the fish body surface was removed completely, the tail was cut off with sterile surgical scissors, blood was taken from capillaries treated with 10 mg/L heparin sodium, and blood samples from every 10th fish were pooled.

Blood glucose assay

After centrifuging the blood samples at 680 × *g* for 3 min at room temperature, the upper plasma layer was aspirated and blood glucose levels were determined using a fully automatic glucometer.

References

1. dos Santos, J., Burkow, I. C., & Jobling, M.. Patterns of growth and lipid deposition in cod (*Gadus morhua* L.) fed natural prey and fish-based feeds. *Aquaculture*, 110(2), 173-189 (1993).
2. Valente, L. M. P., Bandarra, N. M., Figueiredo-Silva, A. C., Cordeiro, A. R., Simoes, R. M., & Nunes, M. L.. Influence of conjugated linoleic acid on growth, lipid composition and hepatic lipogenesis in juvenile European sea bass (*Dicentrarchus labrax*). *Aquaculture*, 267(1-4), 225-235 (2007).
3. Ando S , Mori Y , Nakamura K , et al. Characteristics of Lipid Accumulation Types in Five Species of Fish.[J]. *Nippon Suisan Gakkaishi*, 1993, 59(9):1559-1564.
4. Jeong, C.-B. *et al.* Marine medaka ATP-binding cassette (ABC) superfamily and new insight into teleost Abch nomenclature. *Scientific Reports* **5**(2015).
5. Liu, S., Li, Q. & Liu, Z. Genome-Wide Identification, Characterization and Phylogenetic Analysis of 50 Catfish ATP-Binding Cassette (ABC) Transporter Genes. *Plos One* 8(2013).
6. Adrian-Kalchhauser, I. et al. The round goby genome provides insights into mechanisms that may facilitate biological invasions. *Bmc Biology* 18(1), 1-33 (2020).
7. Solbakken, M.H., Voje, K.L., Jakobsen, K.S. & Jentoft, S. Linking species habitat and past palaeoclimatic events to evolution of the teleost innate immune system. *Proceedings of the Royal Society B-Biological Sciences* **284** (2017).
8. Solbakken, M.H. *et al.* Evolutionary redesign of the Atlantic cod (Gadus morhua L.) Toll-like receptor repertoire by gene losses and expansions. *Scientific Reports* **6**(1), 1-14 (2016).
9. Sardiello, M., Cairo, S., Fontanella, B., Ballabio, A., & Meroni, G. Genomic analysis of the TRIM family reveals two groups of genes with distinct evolutionary properties. BMC evolutionary biology, 8(1), 225 (2008).
10. Pati, N., Schowinsky, V., Kokanovic, O., Magnuson, V. & Ghosh, S. A comparison between SNaPshot, pyrosequencing, and biplex invader SNP genotyping methods: accuracy, cost, and throughput. *Journal of Biochemical and Biophysical Methods* **60**, 1-12 (2004).
11. Cai, L. *et al.* Characterization of transcriptional responses mediated by benzo[a]pyrene stress in a new marine fish model of goby, Mugilogobius chulae. *Genes Genomics* **41**, 113-123 (2019).
