# Supplementary material for: Whole-genome sequencing reveals sex determination and liver high-fat storage mechanisms of yellowstripe goby (Mugilogobius chulae)
Source: Commun Biol. 2021 Jan 4;4:15. doi: 10.1038/s42003-020-01541-9 (PMC7782490; doi:10.1038/s42003-020-01541-9)
Supplement: Supplementary file 3 — Description of Additional Supplementary Files [file 42003_2020_1541_MOESM3_ESM.pdf]

Description of Additional Supplementary Files

File Name: Supplementary Data 1
Description: Orthologous genes among yellowstripe goby, zebrafish, and 15 humans.
