# Supplementary material for: Whole-genome sequencing reveals sex determination and liver high-fat storage mechanisms of yellowstripe goby (Mugilogobius chulae)
Source: Commun Biol. 2021 Jan 4;4:15. doi: 10.1038/s42003-020-01541-9 (PMC7782490; doi:10.1038/s42003-020-01541-9)
Supplement: Supplementary file 4 — Supplementary Data 1 [file 42003_2020_1541_MOESM4_ESM.pdf]

Whole-genome sequencing reveals sex determination and liver high-fat storage mechanisms of yellowstripe goby (*Mugilogobius chulae*)

Lei Cai[1 †*], Guocheng Liu[2†], Yuanzheng Wei[1†], Yabing Zhu[2†], Jianjun Li[1], Zongyu Miao[1], Meili Chen[1], Zhen Yue[2], Lujun Yu[1], Zhensheng Dong[2], Huixin Ye[1], Wenjing Sun[2], Ren Huang[1*]

[1]Guangdong Provincial Key Laboratory of Laboratory Animals, Guangdong Laboratory Animals Monitoring Institute, Guangzhou, China.
[2]BGI Genomics, BGI-Shenzhen, Shenzhen, China.
[†] These authors contributed equally to this manuscript.
*Correspondence and requests for materials should be addressed to L.C. (email: cailei17@163.com) or to R.H. (email: 1649405216@qq.com).

Supplementary Data 1 Orthologous genes among yellowstripe goby, zebrafish, and humans

fam_id (M.chulae)	fam_id (zebrafish)	fam_id (human)
8	8	3
13	12	12
17	13	13
25	17	17
32	19	19
33	32	31
40	40	32
41	41	40
43	43	43
52	49	52
56	71	57
72	75	75
78	82	82
82	84	84
84	86	86
86	88	91
87	91	93
91	97	97
97	100	122
98	105	126
105	121	134
109	122	139
121	126	146
122	132	153
130	134	154
132	146	157
134	152	163
138	154	170
143	163	175

145	164	195
146	170	198
152	175	223
154	186	231
161	198	234
178	201	238
181	216	242
186	223	243
194	226	247
197	227	258
198	231	262
201	234	277
221	242	287
223	249	289
226	256	290
227	257	291
234	259	298
236	262	307
238	279	312
240	287	313
242	289	317
243	293	320
245	294	344
254	296	359
256	312	370
257	333	380
258	344	384
259	350	391
273	355	392
274	359	399
282	366	405
283	370	407
289	380	408
296	392	409
298	399	412
300	409	417
312	418	418
314	424	424
317	425	425
318	430	430
319	432	432
334	448	435
338	455	440
341	456	454
347	457	458
348	475	463

351	491	475
361	501	501
364	511	511
366	516	512
367	522	516
370	523	522
376	527	523
389	535	527
394	538	535
397	540	539
399	543	540
402	549	549
405	550	550
407	559	559
409	564	560
413	566	564
414	569	569
415	578	573
418	581	577
425	584	582
430	588	583
437	602	586
451	612	588
454	614	589
455	638	596
463	658	597
475	665	602
481	684	614
501	685	638
504	688	651
505	696	654
510	704	665
511	705	684
512	711	685
516	723	688
519	728	689
522	734	703
523	739	704
525	741	705
527	744	709
528	746	711
529	751	723
531	756	724
534	758	729
549	764	730
558	775	739

559	777	741
567	782	744
568	783	757
569	794	763
574	800	764
577	804	771
578	806	776
580	807	778
581	811	782
584	813	783
588	821	791
594	833	804
596	842	806
611	843	807
612	844	809
614	845	819
624	846	833
638	852	842
652	857	843
653	861	844
655	863	845
657	866	846
658	867	852
661	869	857
665	873	860
667	884	864
672	886	866
681	888	869
684	891	873
685	892	878
686	894	886
687	898	888
689	901	889
693	902	891
696	903	892
698	907	894
700	908	902
704	913	903
705	923	907
711	926	908
712	929	912
716	930	913
721	934	917
728	937	926
729	939	929
733	945	934

738	964	937
739	968	939
741	977	944
744	978	945
757	982	952
758	1003	968
764	1004	972
768	1006	977
769	1008	978
771	1031	980
773	1034	986
775	1036	988
783	1040	1003
804	1042	1004
806	1045	1007
807	1046	1008
813	1054	1016
815	1062	1030
817	1068	1031
833	1069	1036
843	1070	1040
844	1073	1045
846	1079	1062
850	1080	1079
852	1083	1080
853	1084	1084
857	1087	1089
858	1094	1094
861	1096	1101
863	1101	1103
864	1107	1105
866	1116	1108
867	1125	1123
869	1131	1125
871	1140	1131
872	1147	1147
877	1148	1148
878	1155	1153
884	1158	1155
886	1169	1158
890	1175	1159
891	1178	1175
892	1179	1176
894	1186	1179
898	1187	1186
901	1191	1187

902	1193	1191
907	1195	1192
911	1197	1197
913	1198	1204
926	1201	1216
934	1204	1221
935	1207	1229
937	1208	1231
939	1216	1259
945	1221	1264
954	1229	1266
955	1232	1280
965	1237	1291
975	1244	1292
977	1246	1300
978	1256	1305
979	1264	1315
980	1266	1316
982	1270	1333
989	1280	1347
990	1295	1348
993	1298	1351
994	1300	1352
999	1301	1354
1003	1303	1359
1004	1304	1361
1007	1306	1362
1008	1323	1365
1019	1325	1366
1024	1338	1368
1030	1341	1370
1033	1344	1377
1036	1346	1379
1040	1347	1380
1045	1353	1385
1046	1361	1387
1054	1362	1389
1066	1366	1390
1084	1370	1391
1090	1380	1394
1094	1382	1396
1096	1383	1398
1105	1384	1399
1108	1385	1402
1110	1387	1403
1112	1390	1404

1122	1393	1410
1123	1397	1411
1131	1398	1432
1140	1399	1436
1147	1402	1438
1156	1403	1439
1158	1404	1440
1159	1405	1441
1162	1411	1442
1165	1412	1446
1175	1413	1448
1178	1438	1455
1179	1440	1458
1182	1442	1459
1184	1443	1465
1186	1446	1468
1191	1447	1488
1193	1448	1494
1194	1455	1495
1195	1458	1503
1197	1468	1506
1198	1479	1507
1204	1485	1509
1206	1486	1539
1212	1489	1545
1219	1494	1547
1221	1495	1553
1232	1498	1561
1242	1504	1575
1250	1507	1576
1253	1509	1577
1254	1510	1605
1255	1511	1624
1256	1536	1630
1258	1537	1631
1264	1539	1635
1270	1542	1643
1279	1545	1661
1280	1548	1665
1287	1562	1667
1291	1564	1668
1301	1565	1673
1304	1575	1676
1319	1576	1692
1322	1579	1697
1336	1595	1701

1337	1603	1702
1340	1605	1707
1341	1616	1710
1345	1630	1711
1346	1631	1717
1347	1634	1718
1353	1635	1722
1360	1637	1728
1361	1643	1734
1362	1666	1736
1363	1667	1741
1364	1668	1742
1366	1671	1780
1370	1677	1791
1374	1702	1797
1380	1706	1802
1382	1710	1805
1383	1717	1806
1385	1722	1807
1387	1725	1808
1390	1734	1810
1398	1736	1823
1399	1740	1824
1401	1742	1829
1402	1751	1837
1403	1762	1847
1404	1780	1848
1411	1790	1854
1412	1791	1859
1413	1802	1865
1424	1803	1869
1431	1805	1884
1432	1806	1886
1439	1807	1893
1440	1808	1894
1441	1810	1904
1442	1811	1912
1445	1824	1950
1446	1837	1957
1447	1839	1961
1450	1847	1973
1455	1848	1977
1458	1859	1982
1459	1860	1987
1461	1865	1989
1468	1869	1991

1485	1872	2001
1488	1874	2002
1489	1880	2006
1494	1886	2007
1498	1893	2012
1500	1901	2013
1509	1904	2014
1510	1912	2020
1513	1950	2025
1521	1957	2036
1524	1963	2037
1531	1973	2040
1544	1977	2050
1545	1978	2053
1548	1986	2054
1549	1987	2057
1553	1989	2062
1560	1994	2079
1564	2001	2082
1565	2002	2094
1566	2005	2096
1569	2006	2103
1570	2013	2116
1572	2014	2117
1573	2020	2123
1575	2025	2132
1578	2031	2136
1581	2036	2141
1594	2040	2144
1595	2050	2167
1597	2053	2176
1603	2054	2179
1605	2057	2180
1609	2058	2181
1613	2076	2188
1615	2096	2192
1616	2097	2196
1617	2103	2199
1626	2106	2200
1631	2117	2201
1634	2123	2203
1643	2132	2204
1661	2136	2209
1666	2141	2211
1667	2144	2215
1668	2145	2245

1672	2167	2257
1678	2180	2261
1681	2181	2297
1693	2188	2301
1703	2196	2323
1706	2198	2330
1709	2199	2335
1717	2200	2337
1720	2203	2341
1722	2204	2343
1728	2207	2347
1734	2209	2350
1736	2210	2351
1740	2214	2352
1741	2215	2353
1743	2241	2358
1746	2242	2361
1756	2243	2396
1758	2245	2398
1761	2250	2400
1765	2270	2403
1771	2297	2404
1773	2301	2411
1775	2302	2412
1782	2306	2413
1783	2308	2414
1786	2321	2415
1788	2322	2424
1789	2325	2426
1790	2330	2437
1792	2336	2440
1794	2343	2442
1799	2347	2443
1805	2350	2456
1806	2363	2459
1807	2375	2465
1808	2377	2472
1810	2385	2474
1813	2392	2479
1817	2397	2480
1823	2399	2484
1834	2400	2487
1842	2401	2488
1843	2403	2490
1846	2411	2492
1847	2412	2506

1848	2413	2513
1852	2414	2514
1857	2415	2519
1860	2426	2522
1865	2431	2524
1869	2437	2527
1872	2442	2528
1880	2443	2531
1884	2456	2547
1889	2458	2554
1893	2459	2558
1900	2465	2564
1901	2479	2565
1902	2490	2568
1904	2492	2570
1906	2505	2571
1912	2509	2584
1923	2513	2585
1932	2514	2587
1939	2519	2588
1945	2522	2589
1949	2524	2590
1953	2525	2593
1956	2530	2602
1957	2556	2614
1962	2558	2619
1966	2565	2622
1973	2567	2628
1976	2568	2632
1982	2589	2648
1987	2590	2665
1989	2593	2667
1991	2597	2673
2001	2601	2676
2002	2607	2688
2016	2608	2690
2025	2615	2695
2036	2618	2697
2040	2619	2698
2050	2622	2700
2057	2628	2702
2062	2630	2708
2072	2632	2718
2081	2634	2731
2115	2640	2732
2120	2648	2767

2123	2671	2770
2131	2673	2772
2132	2676	2773
2133	2688	2791
2136	2690	2793
2141	2692	2795
2143	2695	2801
2145	2698	2811
2148	2702	2815
2167	2707	2816
2175	2715	2818
2178	2718	2819
2180	2720	2820
2188	2732	2822
2199	2780	2823
2200	2782	2824
2201	2789	2828
2203	2795	2833
2204	2797	2839
2207	2798	2840
2209	2799	2847
2218	2800	2851
2220	2801	2853
2232	2808	2860
2233	2811	2862
2245	2815	2864
2246	2816	2866
2262	2819	2873
2263	2822	2883
2264	2823	2889
2267	2825	2900
2268	2828	2902
2269	2831	2904
2270	2832	2912
2273	2840	2915
2274	2847	2916
2280	2851	2918
2281	2853	2919
2287	2862	2923
2289	2864	2931
2293	2866	2940
2300	2883	2946
2302	2890	2948
2310	2899	2952
2316	2903	2953
2317	2905	2955

2321	2914	2957
2322	2915	2962
2326	2919	2971
2330	2922	2988
2336	2926	2991
2341	2933	3002
2345	2937	3003
2347	2940	3004
2349	2943	3015
2361	2948	3016
2364	2952	3017
2370	2954	3019
2374	2957	3020
2377	2962	3026
2385	2971	3030
2396	2983	3037
2397	2984	3041
2403	2988	3044
2409	3002	3047
2412	3004	3048
2414	3015	3049
2415	3016	3050
2431	3017	3051
2433	3020	3052
2435	3027	3053
2437	3029	3056
2442	3037	3061
2443	3041	3065
2456	3042	3068
2458	3044	3073
2465	3047	3083
2469	3048	3084
2471	3049	3086
2477	3050	3106
2478	3051	3111
2479	3052	3113
2480	3053	3116
2482	3061	3134
2485	3065	3136
2486	3066	3141
2488	3075	3149
2496	3083	3151
2506	3084	3158
2509	3086	3172
2514	3096	3173
2522	3104	3186

2524	3112	3188
2525	3116	3190
2532	3134	3191
2536	3136	3206
2538	3141	3212
2543	3142	3213
2545	3144	3215
2549	3149	3218
2551	3150	3221
2552	3151	3226
2553	3160	3228
2556	3170	3230
2558	3172	3231
2559	3173	3233
2564	3176	3239
2568	3184	3243
2571	3190	3248
2583	3193	3255
2588	3202	3257
2589	3205	3262
2593	3206	3265
2596	3207	3267
2601	3215	3275
2602	3221	3281
2604	3231	3286
2607	3239	3287
2609	3255	3288
2614	3257	3300
2618	3267	3301
2619	3280	3305
2622	3283	3314
2623	3286	3315
2626	3288	3323
2630	3299	3326
2632	3300	3327
2634	3305	3328
2638	3314	3332
2641	3316	3333
2643	3317	3336
2648	3319	3337
2665	3323	3338
2673	3327	3340
2676	3328	3341
2688	3332	3342
2689	3337	3344
2696	3338	3348

2698	3341	3353
2699	3344	3357
2701	3348	3365
2705	3352	3371
2708	3353	3372
2715	3357	3383
2718	3362	3384
2720	3365	3388
2730	3367	3389
2731	3383	3391
2732	3388	3393
2756	3389	3396
2758	3391	3404
2769	3393	3407
2783	3396	3414
2784	3403	3418
2794	3404	3422
2797	3413	3429
2799	3414	3435
2801	3418	3438
2803	3427	3440
2811	3436	3441
2813	3440	3459
2815	3441	3475
2816	3459	3481
2817	3472	3482
2819	3474	3488
2820	3481	3489
2821	3489	3491
2822	3491	3492
2823	3492	3496
2825	3496	3499
2826	3499	3509
2828	3500	3510
2831	3504	3511
2838	3507	3513
2839	3509	3515
2840	3511	3520
2847	3513	3525
2853	3515	3526
2856	3519	3527
2862	3520	3528
2866	3521	3529
2869	3525	3530
2872	3526	3536
2873	3527	3537

2885	3528	3539
2889	3529	3547
2891	3530	3568
2893	3531	3569
2896	3534	3570
2899	3537	3573
2900	3539	3574
2902	3540	3576
2903	3547	3580
2904	3570	3591
2905	3573	3607
2906	3574	3610
2910	3576	3615
2914	3580	3626
2917	3591	3627
2919	3607	3638
2920	3610	3646
2922	3618	3647
2923	3626	3656
2932	3646	3658
2937	3647	3665
2940	3656	3676
2952	3658	3677
2953	3664	3678
2957	3676	3686
2969	3677	3689
2970	3678	3690
2971	3680	3697
2981	3686	3700
2984	3688	3703
2988	3691	3712
2990	3692	3718
2993	3697	3723
3000	3703	3724
3001	3705	3734
3004	3712	3740
3008	3713	3742
3014	3717	3743
3015	3718	3744
3016	3723	3745
3017	3733	3746
3021	3735	3748
3027	3743	3750
3029	3744	3755
3033	3745	3756
3037	3746	3759

3040	3748	3761
3041	3750	3763
3042	3755	3769
3044	3756	3771
3048	3757	3772
3049	3759	3778
3051	3767	3780
3052	3768	3786
3060	3769	3787
3061	3770	3791
3063	3772	3795
3064	3778	3801
3065	3779	3808
3066	3780	3813
3067	3786	3815
3073	3787	3818
3083	3801	3820
3086	3808	3821
3089	3812	3822
3105	3813	3830
3106	3821	3832
3108	3822	3836
3111	3826	3837
3112	3833	3840
3116	3837	3842
3118	3840	3845
3119	3845	3847
3121	3847	3849
3130	3848	3850
3134	3849	3852
3136	3850	3853
3142	3852	3856
3149	3853	3860
3151	3854	3866
3153	3855	3871
3158	3866	3872
3168	3871	3877
3170	3872	3880
3172	3880	3881
3175	3887	3893
3177	3893	3897
3179	3896	3901
3184	3898	3904
3188	3901	3908
3190	3904	3909
3193	3907	3912

3199	3909	3916
3201	3913	3920
3202	3916	3924
3206	3919	3925
3207	3920	3929
3212	3924	3931
3214	3925	3934
3215	3927	3938
3216	3929	3950
3219	3931	3952
3221	3935	3954
3223	3938	3960
3225	3939	3965
3226	3948	3972
3228	3950	3975
3236	3952	3976
3239	3954	3981
3243	3960	3993
3246	3972	3994
3251	3983	3996
3252	3984	4001
3257	3988	4002
3265	3993	4013
3267	3997	4018
3269	3999	4021
3271	4002	4028
3278	4006	4031
3288	4011	4034
3289	4013	4036
3296	4015	4043
3298	4018	4053
3299	4021	4058
3301	4028	4062
3317	4034	4065
3319	4043	4066
3323	4049	4068
3326	4058	4071
3327	4059	4074
3328	4062	4075
3331	4065	4077
3337	4066	4085
3338	4070	4088
3340	4072	4090
3342	4074	4094
3344	4075	4095
3348	4076	4108

3351	4077	4109
3352	4080	4115
3353	4085	4116
3354	4088	4127
3357	4089	4132
3362	4090	4134
3366	4094	4137
3367	4095	4138
3371	4105	4150
3372	4106	4151
3383	4108	4161
3384	4109	4163
3388	4115	4166
3389	4116	4170
3391	4118	4180
3393	4123	4188
3403	4127	4193
3404	4132	4194
3414	4137	4195
3418	4138	4197
3420	4143	4201
3424	4155	4203
3425	4163	4209
3429	4165	4212
3430	4179	4214
3436	4180	4215
3440	4185	4217
3442	4193	4220
3446	4195	4222
3449	4197	4224
3455	4199	4225
3464	4201	4226
3472	4212	4227
3475	4214	4228
3476	4217	4230
3481	4224	4235
3489	4225	4236
3491	4226	4238
3492	4227	4240
3494	4228	4241
3496	4233	4242
3497	4235	4243
3499	4236	4246
3500	4238	4247
3501	4240	4251
3505	4241	4277

3509	4243	4278
3511	4246	4279
3515	4247	4281
3518	4251	4283
3519	4279	4284
3520	4282	4287
3525	4284	4288
3526	4287	4289
3528	4288	4293
3529	4289	4294
3530	4294	4296
3531	4296	4299
3532	4297	4301
3533	4301	4303
3537	4303	4304
3539	4304	4307
3540	4305	4314
3554	4307	4315
3557	4311	4323
3561	4315	4324
3570	4323	4325
3576	4324	4326
3584	4325	4327
3585	4326	4335
3592	4327	4338
3594	4336	4340
3607	4337	4342
3608	4338	4345
3610	4339	4348
3612	4345	4351
3615	4348	4352
3621	4352	4354
3625	4354	4357
3626	4357	4367
3627	4367	4368
3631	4368	4369
3635	4369	4372
3645	4372	4373
3646	4373	4374
3647	4374	4376
3650	4376	4384
3656	4380	4385
3658	4384	4387
3664	4385	4388
3667	4387	4391
3668	4391	4393

3671	4392	4394
3676	4393	4395
3677	4394	4401
3678	4395	4411
3680	4399	4413
3681	4401	4417
3685	4407	4418
3688	4413	4420
3691	4421	4421
3695	4425	4425
3697	4430	4426
3700	4432	4432
3703	4443	4433
3707	4445	4443
3716	4446	4444
3718	4448	4446
3721	4449	4447
3728	4452	4448
3731	4457	4449
3732	4459	4452
3735	4462	4455
3740	4466	4456
3742	4482	4457
3743	4484	4459
3746	4486	4462
3748	4489	4463
3750	4504	4466
3752	4505	4474
3753	4510	4476
3754	4518	4482
3755	4520	4484
3756	4522	4489
3759	4527	4501
3761	4535	4503
3762	4547	4504
3765	4549	4505
3767	4550	4510
3768	4551	4518
3771	4552	4520
3775	4554	4522
3778	4556	4527
3780	4559	4530
3785	4568	4535
3787	4570	4538
3799	4571	4547
3801	4578	4549

3802	4586	4550
3804	4587	4551
3808	4589	4554
3813	4591	4556
3822	4594	4558
3827	4600	4559
3831	4602	4561
3833	4603	4562
3837	4608	4565
3840	4615	4570
3847	4618	4571
3848	4619	4578
3849	4627	4581
3851	4637	4585
3855	4644	4587
3866	4653	4589
3871	4665	4592
3873	4669	4600
3874	4678	4602
3877	4682	4603
3880	4685	4608
3881	4686	4615
3883	4692	4618
3885	4698	4620
3886	4707	4625
3887	4708	4635
3893	4719	4644
3901	4736	4653
3904	4739	4658
3911	4747	4664
3916	4755	4666
3920	4758	4697
3924	4762	4698
3927	4770	4712
3932	4771	4755
3933	4772	4758
3935	4777	4760
3938	4779	4761
3939	4780	4770
3942	4791	4777
3943	4792	4778
3945	4793	4779
3948	4798	4785
3952	4802	4786
3958	4818	4791
3960	4819	4800

3967	4826	4813
3979	4827	4819
3981	4829	4821
3983	4830	4826
3993	4834	4827
4004	4839	4829
4011	4840	4830
4013	4843	4837
4017	4845	4840
4018	4849	4849
4021	4851	4850
4022	4855	4851
4023	4863	4853
4025	4864	4854
4031	4867	4855
4034	4873	4880
4043	4874	4883
4045	4881	4894
4046	4885	4895
4049	4887	4896
4051	4896	4901
4052	4897	4902
4054	4898	4905
4059	4902	4906
4061	4904	4907
4062	4905	4908
4068	4908	4909
4070	4909	4914
4071	4914	4920
4072	4924	4924
4073	4925	4928
4074	4927	4953
4075	4928	4956
4087	4931	4961
4089	4941	4980
4091	4945	4984
4093	4946	4998
4094	4947	5006
4096	4948	5037
4098	4949	5039
4105	4953	5042
4108	4956	5044
4109	4961	5050
4113	4970	5056
4114	4973	5060
4116	4982	5061

4121	4984	5062
4123	4996	5071
4124	5005	5072
4127	5009	5076
4132	5014	5077
4134	5019	5078
4137	5022	5079
4145	5026	5085
4160	5034	5087
4166	5037	5089
4179	5039	5091
4180	5042	5099
4185	5056	5106
4187	5060	5107
4190	5061	5108
4193	5062	5110
4195	5068	5123
4196	5071	5124
4200	5075	5125
4201	5076	5128
4203	5077	5132
4212	5078	5138
4214	5080	5139
4217	5087	5141
4220	5089	5145
4224	5093	5147
4225	5099	5160
4226	5100	5162
4227	5106	5180
4230	5107	5193
4232	5109	5197
4235	5111	5204
4236	5123	5208
4240	5124	5226
4241	5125	5227
4242	5127	5231
4243	5128	5238
4246	5132	5246
4247	5134	5247
4251	5136	5248
4267	5145	5249
4276	5150	5252
4277	5151	5256
4279	5165	5260
4280	5193	5262
4281	5197	5264

4284	5205	5265
4287	5208	5289
4289	5209	5290
4294	5225	5299
4296	5231	5304
4301	5234	5306
4302	5238	5308
4304	5247	5309
4307	5248	5322
4309	5251	5333
4311	5255	5335
4314	5256	5340
4316	5257	5345
4318	5260	5352
4323	5262	5355
4324	5264	5357
4326	5281	5366
4327	5284	5372
4328	5288	5375
4330	5299	5378
4334	5300	5379
4338	5302	5380
4342	5308	5383
4346	5309	5386
4350	5312	5387
4353	5333	5388
4354	5335	5389
4357	5340	5396
4358	5343	5404
4359	5350	5412
4363	5353	5413
4368	5355	5414
4369	5357	5418
4373	5366	5420
4374	5375	5423
4380	5383	5433
4382	5387	5439
4384	5388	5451
4385	5389	5455
4387	5396	5456
4389	5404	5457
4391	5414	5460
4392	5420	5464
4393	5426	5472
4394	5433	5495
4395	5439	5498

4397	5454	5499
4402	5455	5504
4411	5456	5508
4415	5457	5516
4419	5460	5519
4421	5463	5521
4425	5464	5524
4430	5465	5525
4433	5497	5531
4437	5502	5532
4438	5504	5533
4441	5506	5541
4442	5516	5543
4443	5519	5548
4444	5521	5551
4445	5524	5554
4449	5525	5557
4452	5531	5564
4458	5532	5565
4459	5533	5586
4461	5534	5596
4466	5541	5602
4471	5543	5604
4474	5551	5605
4476	5553	5607
4482	5556	5618
4484	5559	5621
4486	5564	5627
4487	5565	5628
4489	5568	5629
4491	5586	5636
4504	5591	5638
4510	5593	5641
4518	5594	5642
4519	5604	5644
4520	5607	5647
4526	5620	5650
4527	5627	5656
4539	5628	5661
4541	5629	5673
4546	5633	5674
4549	5637	5678
4550	5638	5682
4551	5641	5684
4554	5643	5686
4557	5647	5688

4558	5650	5692
4561	5651	5693
4562	5656	5695
4565	5661	5699
4567	5665	5701
4571	5673	5704
4578	5674	5709
4581	5675	5712
4582	5678	5713
4583	5682	5716
4585	5684	5724
4586	5686	5732
4587	5688	5733
4589	5689	5734
4601	5692	5735
4603	5693	5739
4608	5699	5740
4611	5701	5744
4615	5704	5745
4618	5707	5746
4620	5709	5749
4622	5712	5750
4626	5713	5761
4637	5720	5763
4640	5722	5787
4641	5724	5791
4644	5728	5795
4652	5732	5799
4655	5733	5800
4664	5734	5806
4666	5736	5810
4681	5739	5811
4682	5742	5815
4684	5745	5817
4685	5749	5818
4696	5750	5823
4697	5754	5828
4698	5763	5835
4701	5783	5843
4708	5787	5847
4713	5791	5848
4731	5792	5849
4739	5795	5850
4749	5800	5851
4751	5802	5855
4752	5806	5856

4754	5809	5883
4755	5810	5886
4757	5811	5901
4759	5818	5902
4760	5820	5928
4761	5823	5930
4763	5828	5931
4771	5829	5944
4773	5835	5962
4777	5847	5966
4780	5848	5967
4781	5849	5968
4782	5850	5969
4784	5856	5972
4785	5862	5974
4802	5883	5984
4810	5902	5985
4826	5919	5986
4828	5923	5988
4829	5934	5992
4840	5935	5994
4843	5939	5995
4855	5965	6006
4864	5966	6007
4867	5967	6009
4870	5969	6010
4872	5972	6011
4875	5974	6012
4880	5976	6014
4885	5985	6015
4888	5986	6021
4891	5989	6024
4895	5992	6025
4897	5994	6029
4901	5995	6030
4902	6004	6047
4904	6006	6055
4907	6007	6059
4908	6011	6060
4909	6012	6061
4918	6014	6066
4920	6015	6069
4923	6029	6071
4924	6030	6072
4925	6043	6073
4927	6045	6074

4938	6049	6075
4939	6054	6078
4946	6058	6079
4948	6059	6080
4950	6060	6088
4953	6061	6089
4954	6063	6091
4961	6065	6092
4975	6066	6093
4980	6069	6104
4984	6072	6118
4985	6073	6123
4986	6074	6130
5003	6075	6135
5010	6076	6136
5015	6079	6137
5016	6080	6145
5020	6084	6146
5021	6092	6147
5022	6093	6148
5023	6104	6150
5024	6109	6152
5027	6116	6154
5037	6117	6156
5039	6118	6157
5048	6130	6159
5055	6135	6163
5056	6144	6165
5060	6145	6167
5061	6146	6168
5073	6147	6169
5074	6148	6170
5076	6149	6171
5077	6150	6172
5078	6152	6173
5079	6153	6174
5080	6154	6175
5084	6156	6176
5087	6157	6177
5089	6159	6188
5091	6161	6192
5093	6163	6193
5097	6165	6194
5099	6167	6204
5100	6168	6206
5101	6169	6207

5106	6170	6208
5109	6171	6209
5111	6173	6210
5114	6174	6211
5118	6176	6212
5121	6177	6215
5124	6187	6221
5125	6188	6229
5128	6190	6237
5130	6192	6240
5137	6193	6253
5138	6194	6263
5141	6206	6284
5145	6207	6285
5147	6208	6286
5148	6212	6287
5157	6224	6297
5158	6225	6301
5159	6230	6303
5161	6234	6304
5168	6235	6306
5173	6238	6308
5174	6239	6310
5175	6251	6314
5180	6253	6315
5182	6263	6318
5183	6285	6320
5186	6290	6325
5189	6294	6330
5190	6297	6336
5193	6301	6342
5194	6302	6352
5195	6306	6353
5198	6308	6356
5203	6314	6365
5206	6315	6370
5208	6318	6374
5209	6326	6384
5210	6332	6386
5211	6342	6388
5213	6348	6389
5214	6351	6391
5222	6352	6395
5225	6353	6398
5228	6361	6402
5233	6365	6405

5234	6384	6406
5236	6385	6407
5247	6386	6409
5248	6391	6410
5252	6395	6417
5255	6396	6422
5256	6398	6424
5257	6402	6428
5262	6405	6431
5264	6408	6433
5286	6410	6435
5288	6417	6440
5290	6419	6443
5296	6422	6445
5299	6423	6446
5305	6425	6447
5308	6428	6449
5315	6430	6452
5316	6431	6453
5321	6433	6454
5332	6435	6455
5333	6440	6456
5335	6444	6457
5340	6445	6462
5343	6446	6463
5345	6449	6465
5353	6452	6466
5355	6455	6468
5357	6456	6470
5364	6457	6483
5380	6459	6485
5383	6462	6488
5388	6463	6492
5389	6464	6494
5393	6465	6496
5394	6466	6498
5397	6468	6505
5404	6483	6522
5414	6485	6526
5420	6488	6533
5426	6491	6538
5427	6492	6541
5430	6493	6558
5433	6494	6561
5439	6496	6563
5451	6498	6564

5452	6505	6565
5453	6521	6568
5454	6526	6569
5455	6533	6584
5459	6543	6586
5460	6560	6596
5464	6561	6601
5468	6564	6609
5473	6565	6612
5474	6567	6621
5484	6569	6625
5498	6575	6638
5502	6578	6642
5503	6580	6643
5504	6584	6647
5516	6586	6649
5519	6591	6652
5521	6595	6657
5525	6596	6660
5531	6597	6663
5532	6598	6672
5533	6603	6684
5534	6604	6685
5536	6608	6686
5540	6609	6697
5541	6612	6700
5543	6617	6705
5545	6619	6706
5551	6621	6714
5553	6638	6715
5555	6639	6725
5556	6643	6731
5562	6647	6737
5564	6651	6739
5565	6657	6748
5568	6659	6749
5586	6660	6754
5593	6663	6756
5596	6668	6761
5600	6675	6765
5602	6684	6769
5606	6685	6771
5607	6686	6772
5616	6698	6776
5618	6699	6779
5622	6701	6780

5626	6715	6782
5631	6717	6784
5634	6718	6791
5641	6720	6792
5643	6739	6793
5645	6740	6798
5647	6748	6807
5650	6749	6812
5658	6761	6817
5661	6765	6819
5663	6772	6820
5664	6781	6821
5671	6782	6825
5672	6788	6826
5673	6789	6828
5674	6798	6836
5675	6804	6839
5678	6807	6842
5681	6808	6844
5684	6810	6864
5689	6811	6865
5691	6816	6870
5693	6819	6878
5697	6820	6881
5698	6821	6889
5699	6825	6891
5703	6828	6893
5704	6839	6897
5712	6842	6898
5713	6844	6900
5714	6864	6901
5715	6870	6909
5720	6878	6910
5721	6881	6919
5724	6889	6924
5728	6895	6937
5731	6897	6939
5732	6898	6942
5733	6900	6949
5734	6905	6950
5738	6907	6951
5739	6909	6952
5741	6910	6953
5745	6911	6961
5746	6913	6968
5754	6917	6970

5767	6922	6971
5768	6924	6977
5769	6929	6979
5772	6937	6980
5773	6939	6982
5776	6940	6983
5777	6944	6985
5778	6949	6990
5779	6950	6999
5782	6952	7000
5785	6953	7003
5791	6957	7004
5795	6961	7005
5798	6964	7007
5800	6970	7024
5806	6971	7029
5810	6977	7041
5811	6980	7049
5818	6983	7050
5821	6985	7051
5823	6990	7054
5825	6993	7056
5828	6995	7057
5829	6996	7059
5833	6999	7064
5834	7000	7066
5835	7003	7067
5840	7005	7070
5846	7014	7071
5847	7019	7072
5848	7024	7073
5849	7028	7075
5850	7029	7082
5854	7031	7089
5855	7032	7092
5856	7041	7096
5857	7049	7100
5858	7050	7105
5866	7051	7111
5869	7054	7113
5871	7056	7114
5881	7057	7115
5883	7059	7120
5886	7064	7124
5889	7066	7130
5890	7067	7133

5902	7070	7161
5904	7072	7164
5908	7074	7165
5921	7075	7168
5925	7082	7171
5932	7083	7172
5933	7085	7174
5936	7092	7177
5940	7093	7178
5942	7096	7181
5944	7105	7182
5945	7107	7185
5947	7109	7188
5949	7110	7189
5954	7120	7190
5955	7130	7191
5956	7133	7192
5964	7161	7196
5967	7165	7197
5972	7172	7201
5973	7178	7203
5974	7181	7206
5980	7185	7212
5981	7188	7216
5982	7189	7218
5984	7192	7219
5992	7196	7221
5994	7197	7227
6001	7202	7229
6002	7203	7236
6010	7204	7244
6011	7212	7247
6012	7216	7254
6014	7219	7256
6018	7221	7258
6025	7227	7262
6026	7229	7263
6028	7233	7264
6029	7239	7267
6030	7240	7269
6033	7247	7277
6042	7254	7280
6045	7255	7284
6046	7256	7286
6048	7258	7294
6061	7263	7297

6065	7267	7298
6066	7269	7300
6068	7273	7313
6069	7280	7315
6070	7282	7317
6072	7285	7323
6073	7286	7327
6074	7293	7331
6075	7294	7338
6076	7298	7340
6077	7300	7344
6080	7303	7347
6084	7312	7349
6086	7313	7350
6088	7314	7351
6090	7317	7355
6094	7323	7356
6095	7327	7358
6104	7330	7361
6116	7331	7363
6117	7333	7364
6120	7334	7368
6131	7338	7376
6135	7339	7381
6145	7340	7384
6146	7347	7386
6148	7349	7392
6149	7350	7400
6150	7355	7402
6153	7356	7405
6154	7357	7406
6156	7361	7413
6157	7363	7417
6159	7364	7418
6163	7367	7419
6164	7368	7421
6166	7376	7422
6167	7379	7423
6169	7385	7424
6170	7386	7426
6173	7392	7429
6174	7395	7438
6176	7400	7440
6177	7402	7441
6181	7404	7442
6186	7405	7449

6187	7406	7461
6188	7417	7468
6189	7419	7469
6191	7424	7477
6192	7426	7481
6193	7428	7484
6194	7429	7487
6195	7440	7490
6207	7443	7494
6208	7449	7497
6211	7457	7498
6225	7461	7500
6230	7468	7505
6248	7470	7506
6259	7477	7508
6260	7481	7510
6262	7487	7519
6263	7490	7523
6264	7494	7529
6268	7498	7534
6296	7501	7536
6303	7502	7539
6318	7504	7541
6319	7505	7550
6322	7506	7555
6333	7508	7561
6336	7510	7563
6339	7517	7564
6342	7519	7568
6348	7523	7599
6352	7525	7601
6353	7529	7603
6365	7532	7604
6371	7534	7615
6376	7536	7616
6378	7539	7617
6382	7541	7620
6383	7543	7624
6384	7545	7625
6391	7550	7626
6395	7555	7627
6396	7560	7628
6397	7568	7629
6398	7579	7636
6402	7580	7639
6404	7581	7655

6407	7586	7659
6409	7591	7661
6410	7597	7664
6415	7615	7671
6419	7616	7676
6420	7624	7677
6421	7626	7679
6422	7630	7680
6428	7636	7684
6431	7640	7696
6435	7655	7702
6440	7659	7704
6442	7660	7705
6444	7661	7706
6446	7664	7708
6448	7668	7721
6452	7671	7723
6455	7673	7725
6456	7677	7727
6459	7679	7728
6462	7680	7733
6466	7684	7743
6470	7692	7745
6478	7700	7748
6483	7702	7749
6485	7704	7752
6488	7705	7754
6490	7706	7755
6491	7708	7756
6492	7717	7757
6493	7725	7760
6497	7727	7762
6499	7732	7764
6503	7737	7771
6505	7743	7773
6507	7745	7778
6509	7747	7783
6521	7749	7785
6526	7750	7789
6532	7751	7797
6533	7752	7798
6538	7755	7816
6541	7757	7820
6543	7759	7832
6548	7762	7833
6554	7763	7836

6556	7765	7837
6560	7770	7838
6561	7771	7843
6564	7773	7847
6566	7774	7848
6569	7779	7851
6574	7783	7856
6578	7789	7861
6580	7797	7869
6584	7798	7870
6586	7815	7874
6588	7826	7875
6590	7832	7876
6595	7833	7877
6604	7837	7878
6606	7838	7879
6612	7839	7889
6613	7841	7895
6617	7842	7897
6636	7843	7899
6639	7846	7902
6640	7847	7904
6642	7848	7906
6644	7851	7907
6648	7853	7913
6649	7854	7914
6651	7856	7915
6656	7861	7917
6657	7869	7919
6660	7874	7923
6662	7875	7938
6665	7893	7939
6667	7894	7940
6669	7895	7942
6670	7896	7948
6672	7897	7958
6675	7898	7960
6678	7901	7969
6684	7902	7971
6688	7904	7974
6694	7907	7975
6696	7909	7976
6699	7913	7978
6703	7914	7979
6709	7916	7981
6712	7917	7997

6714	7919	7999
6715	7923	8004
6718	7934	8005
6720	7939	8010
6726	7940	8013
6729	7942	8018
6735	7948	8021
6739	7968	8029
6742	7971	8031
6748	7974	8033
6749	7978	8034
6750	7979	8041
6752	7980	8042
6753	7981	8043
6756	7983	8047
6760	7994	8048
6761	7997	8050
6765	7999	8056
6766	8004	8059
6769	8005	8066
6770	8006	8067
6784	8009	8069
6786	8010	8070
6788	8013	8073
6800	8014	8074
6814	8017	8078
6815	8030	8081
6816	8032	8082
6820	8034	8092
6821	8041	8094
6824	8042	8100
6825	8045	8103
6826	8048	8104
6827	8050	8107
6832	8052	8113
6833	8056	8116
6836	8058	8126
6842	8059	8127
6843	8066	8133
6844	8067	8134
6852	8069	8135
6858	8074	8138
6863	8077	8144
6872	8078	8147
6875	8081	8157
6876	8082	8158

6877	8088	8162
6879	8092	8163
6881	8094	8164
6893	8100	8166
6897	8107	8168
6900	8116	8170
6901	8130	8172
6902	8134	8174
6903	8135	8175
6907	8138	8176
6909	8147	8178
6910	8153	8181
6911	8157	8184
6912	8162	8191
6914	8163	8192
6917	8168	8194
6919	8169	8202
6923	8170	8205
6924	8175	8207
6939	8178	8209
6941	8182	8212
6943	8183	8216
6944	8192	8222
6949	8194	8223
6950	8202	8226
6954	8205	8261
6955	8207	8272
6959	8209	8273
6962	8212	8277
6970	8215	8286
6977	8220	8287
6980	8223	8292
6982	8226	8303
6986	8261	8327
6988	8273	8331
6999	8277	8339
7000	8286	8347
7003	8292	8349
7014	8324	8350
7024	8331	8351
7026	8335	8354
7032	8339	8357
7036	8342	8359
7041	8344	8363
7043	8347	8365
7049	8349	8370

7050	8350	8371
7051	8351	8372
7054	8353	8373
7057	8357	8375
7059	8358	8376
7060	8359	8381
7061	8363	8384
7065	8364	8387
7066	8365	8390
7067	8369	8391
7072	8371	8393
7074	8372	8396
7085	8376	8398
7092	8379	8401
7093	8384	8408
7096	8385	8412
7105	8387	8414
7107	8390	8415
7108	8391	8416
7109	8392	8421
7111	8393	8428
7119	8396	8429
7120	8398	8431
7122	8399	8432
7126	8401	8436
7129	8408	8437
7130	8415	8444
7137	8416	8452
7143	8421	8453
7147	8426	8455
7155	8428	8457
7161	8429	8458
7163	8432	8459
7164	8436	8460
7168	8437	8462
7171	8442	8464
7173	8443	8465
7175	8444	8472
7178	8450	8475
7180	8453	8476
7181	8455	8477
7185	8458	8482
7187	8459	8483
7188	8460	8485
7191	8462	8487
7192	8476	8488

7194	8477	8489
7196	8487	8490
7202	8488	8491
7203	8490	8492
7214	8491	8493
7215	8492	8496
7217	8496	8500
7220	8497	8501
7221	8500	8505
7227	8505	8507
7228	8509	8509
7229	8511	8511
7236	8512	8512
7239	8514	8514
7240	8522	8518
7242	8525	8522
7253	8526	8525
7254	8532	8526
7255	8536	8532
7257	8537	8535
7258	8538	8536
7260	8540	8538
7263	8542	8540
7274	8545	8542
7284	8546	8546
7287	8549	8550
7291	8550	8554
7294	8554	8556
7297	8558	8558
7298	8563	8563
7303	8564	8564
7306	8565	8565
7307	8568	8568
7314	8571	8573
7315	8572	8574
7316	8573	8575
7318	8575	8576
7319	8576	8577
7321	8577	8580
7322	8580	8583
7323	8583	8584
7325	8584	8585
7326	8591	8587
7327	8593	8592
7330	8595	8595
7331	8596	8596

7333	8597	8597
7338	8598	8598
7347	8603	8603
7349	8604	8608
7355	8605	8613
7357	8613	8616
7361	8616	8617
7363	8617	8623
7365	8624	8624
7367	8631	8630
7368	8636	8631
7370	8646	8646
7371	8648	8648
7379	8649	8649
7385	8653	8653
7386	8658	8659
7388	8659	8665
7389	8665	8667
7392	8667	8668
7395	8668	8672
7400	8675	8673
7404	8678	8675
7406	8679	8678
7415	8680	8679
7418	8681	8680
7419	8683	8681
7426	8685	8682
7427	8686	8683
7429	8687	8685
7431	8689	8687
7432	8690	8689
7435	8693	8693
7437	8696	8696
7438	8701	8701
7439	8702	8702
7441	8705	8707
7442	8707	8709
7449	8709	8711
7452	8711	8715
7458	8718	8718
7460	8720	8719
7461	8721	8720
7468	8723	8722
7470	8724	8732
7473	8731	8738
7477	8732	8740

7481	8739	8741
7484	8745	8744
7487	8746	8745
7490	8748	8746
7498	8750	8749
7500	8752	8750
7502	8756	8752
7503	8760	8756
7506	8765	8761
7508	8766	8765
7512	8767	8767
7517	8769	8769
7520	8774	8771
7521	8775	8774
7523	8776	8778
7525	8778	8779
7528	8779	8780
7529	8780	8782
7532	8782	8783
7534	8784	8784
7536	8788	8793
7541	8790	8795
7543	8795	8799
7548	8799	8804
7551	8804	8805
7555	8805	8806
7564	8807	8808
7567	8808	8814
7568	8814	8820
7576	8821	8821
7577	8822	8822
7579	8823	8823
7583	8824	8824
7588	8826	8826
7593	8827	8827
7594	8832	8828
7601	8833	8831
7602	8834	8832
7607	8837	8833
7609	8838	8834
7610	8841	8837
7611	8844	8838
7614	8848	8844
7615	8850	8847
7624	8867	8848
7626	8868	8850

7628	8869	8854
7634	8877	8857
7636	8882	8858
7639	8885	8866
7640	8888	8867
7641	8890	8868
7645	8892	8869
7647	8893	8870
7652	8895	8871
7657	8897	8872
7658	8898	8877
7660	8899	8881
7664	8900	8882
7670	8901	8886
7671	8905	8888
7673	8909	8890
7676	8910	8893
7678	8911	8895
7680	8913	8896
7691	8915	8897
7696	8919	8898
7697	8921	8899
7701	8922	8901
7702	8924	8909
7703	8925	8910
7704	8929	8911
7706	8930	8914
7715	8932	8915
7716	8937	8921
7718	8938	8922
7720	8942	8924
7725	8944	8928
7727	8946	8930
7728	8947	8932
7732	8950	8933
7734	8953	8935
7749	8954	8937
7750	8955	8938
7752	8960	8940
7754	8962	8943
7755	8963	8944
7770	8968	8946
7771	8969	8947
7773	8971	8948
7779	8973	8950
7781	8976	8953

7784	8977	8954
7793	8979	8955
7795	8981	8960
7797	8983	8962
7798	8985	8963
7810	8986	8965
7818	8988	8969
7820	8989	8971
7822	8990	8973
7823	8992	8976
7826	8999	8977
7828	9001	8979
7832	9004	8981
7836	9007	8982
7837	9008	8983
7838	9009	8985
7841	9010	8986
7842	9013	8989
7843	9018	8990
7846	9019	8991
7847	9020	8992
7853	9021	8998
7854	9029	9001
7856	9034	9004
7860	9035	9007
7861	9037	9011
7869	9039	9012
7870	9042	9013
7877	9045	9018
7895	9047	9019
7897	9056	9020
7898	9057	9021
7901	9061	9025
7917	9064	9028
7919	9067	9029
7923	9068	9032
7931	9072	9033
7939	9073	9034
7940	9079	9037
7942	9081	9040
7944	9083	9042
7945	9088	9044
7947	9089	9045
7948	9091	9047
7953	9095	9049
7958	9097	9056

7960	9100	9061
7968	9105	9062
7970	9107	9063
7971	9111	9064
7973	9112	9068
7977	9117	9072
7978	9118	9073
7979	9119	9080
7981	9121	9083
7988	9123	9084
7994	9128	9085
7999	9134	9087
8004	9138	9089
8010	9139	9091
8013	9146	9096
8014	9147	9097
8017	9154	9100
8020	9155	9102
8024	9156	9105
8027	9157	9106
8028	9158	9107
8031	9161	9110
8035	9162	9115
8037	9165	9117
8038	9166	9118
8042	9167	9119
8048	9168	9121
8050	9169	9123
8054	9172	9133
8059	9173	9134
8060	9179	9137
8065	9181	9138
8067	9182	9139
8069	9183	9146
8070	9187	9147
8072	9191	9148
8074	9192	9149
8077	9193	9150
8078	9194	9154
8081	9195	9156
8085	9201	9157
8092	9208	9163
8093	9209	9165
8094	9211	9166
8097	9213	9167
8104	9215	9168

8107	9218	9171
8116	9219	9172
8125	9226	9173
8129	9227	9177
8135	9228	9179
8144	9229	9181
8147	9231	9182
8152	9233	9183
8153	9236	9186
8154	9239	9191
8157	9242	9192
8162	9243	9193
8163	9245	9194
8168	9249	9195
8169	9250	9197
8170	9251	9201
8175	9257	9208
8176	9262	9209
8185	9266	9211
8189	9267	9213
8192	9268	9218
8194	9281	9221
8197	9284	9226
8205	9285	9228
8221	9287	9231
8223	9290	9233
8232	9291	9235
8260	9292	9236
8262	9293	9243
8265	9294	9245
8272	9297	9249
8273	9298	9250
8274	9300	9251
8276	9303	9262
8277	9305	9266
8285	9307	9268
8286	9309	9273
8292	9310	9277
8301	9312	9284
8305	9313	9285
8314	9314	9286
8324	9317	9290
8332	9319	9291
8333	9322	9292
8335	9323	9293
8339	9326	9294

8347	9328	9297
8349	9329	9299
8350	9333	9300
8351	9334	9301
8353	9336	9303
8354	9337	9304
8357	9342	9305
8369	9343	9307
8370	9349	9308
8371	9350	9309
8375	9353	9312
8376	9360	9313
8381	9361	9314
8386	9367	9319
8387	9369	9321
8390	9381	9323
8391	9387	9326
8393	9390	9328
8394	9396	9329
8396	9400	9334
8397	9401	9339
8401	9406	9342
8405	9407	9349
8407	9411	9351
8408	9412	9362
8415	9413	9366
8416	9416	9367
8421	9417	9369
8428	9418	9375
8436	9419	9377
8437	9420	9380
8440	9421	9381
8452	9423	9387
8453	9424	9398
8455	9430	9400
8458	9432	9401
8459	9435	9406
8460	9437	9407
8461	9441	9411
8476	9442	9413
8481	9443	9415
8486	9447	9416
8487	9448	9418
8488	9461	9419
8489	9467	9420
8490	9470	9421

8491	9477	9423
8492	9481	9424
8493	9482	9430
8501	9491	9431
8505	9492	9433
8509	9494	9435
8511	9495	9437
8512	9499	9442
8514	9500	9443
8515	9502	9447
8520	9504	9458
8522	9506	9467
8523	9507	9469
8525	9512	9470
8526	9517	9471
8528	9519	9477
8529	9522	9481
8532	9524	9482
8535	9525	9485
8536	9530	9492
8538	9532	9495
8540	9534	9499
8542	9539	9500
8545	9540	9501
8546	9541	9504
8549	9543	9506
8550	9544	9507
8553	9547	9512
8557	9548	9517
8558	9549	9519
8560	9550	9522
8563	9553	9523
8564	9555	9524
8565	9558	9525
8568	9561	9527
8569	9562	9530
8572	9563	9532
8573	9566	9534
8575	9568	9539
8576	9569	9540
8577	9570	9541
8580	9572	9543
8582	9573	9544
8583	9577	9546
8584	9578	9548
8586	9579	9549

8587	9580	9550
8591	9582	9551
8593	9586	9552
8595	9588	9555
8596	9589	9561
8597	9590	9562
8598	9591	9563
8603	9599	9566
8604	9601	9568
8606	9604	9569
8607	9608	9570
8608	9609	9572
8609	9610	9573
8611	9611	9576
8612	9614	9577
8613	9616	9579
8616	9622	9586
8621	9626	9590
8622	9631	9596
8623	9632	9597
8630	9633	9598
8631	9634	9599
8632	9635	9601
8633	9636	9604
8635	9638	9606
8640	9642	9607
8643	9643	9608
8648	9644	9609
8649	9652	9610
8650	9655	9612
8653	9659	9614
8657	9661	9616
8658	9663	9622
8659	9664	9626
8665	9665	9627
8666	9667	9628
8667	9671	9632
8668	9675	9633
8671	9676	9634
8672	9681	9636
8673	9682	9640
8680	9683	9642
8681	9688	9643
8683	9689	9652
8684	9693	9654
8685	9695	9655

8686	9697	9659
8687	9701	9661
8689	9703	9664
8690	9708	9665
8691	9709	9667
8693	9710	9668
8696	9717	9669
8701	9720	9672
8702	9725	9673
8703	9726	9675
8705	9727	9676
8707	9728	9678
8710	9729	9681
8711	9735	9682
8718	9737	9686
8719	9744	9688
8720	9748	9689
8721	9749	9692
8723	9755	9693
8732	9756	9695
8733	9759	9696
8735	9760	9697
8737	9771	9701
8745	9772	9704
8746	9773	9710
8747	9774	9711
8748	9776	9712
8750	9777	9717
8752	9783	9726
8756	9784	9727
8760	9785	9729
8761	9786	9735
8765	9787	9737
8767	9788	9748
8769	9797	9749
8770	9807	9752
8774	9811	9756
8775	9812	9759
8776	9817	9761
8778	9818	9764
8779	9819	9770
8780	9823	9771
8782	9826	9772
8783	9827	9785
8784	9828	9786
8793	9829	9787

8795	9833	9789
8798	9834	9797
8799	9836	9807
8804	9837	9811
8806	9840	9812
8814	9844	9817
8815	9846	9818
8819	9847	9819
8821	9854	9823
8822	9858	9827
8826	9859	9828
8827	9864	9829
8832	9868	9831
8833	9870	9835
8834	9871	9836
8836	9875	9837
8837	9880	9839
8838	9882	9840
8844	9883	9844
8845	9885	9846
8850	9886	9850
8864	9888	9851
8865	9889	9855
8866	9890	9856
8867	9893	9858
8868	9894	9859
8869	9895	9868
8870	9901	9870
8874	9904	9871
8877	9907	9875
8881	9908	9882
8885	9909	9883
8886	9913	9885
8888	9914	9886
8889	9928	9887
8890	9929	9888
8892	9933	9889
8893	9935	9890
8895	9936	9891
8897	9937	9893
8898	9938	9894
8899	9939	9895
8900	9940	9906
8901	9942	9908
8903	9944	9913
8905	9947	9914

8909	9952	9922
8910	9954	9929
8913	9955	9933
8914	9957	9934
8915	9961	9935
8917	9962	9936
8919	9963	9939
8921	9965	9940
8924	9968	9943
8928	9971	9944
8929	9972	9949
8936	9973	9952
8937	9975	9954
8938	9976	9955
8940	9980	9957
8946	9983	9960
8947	9984	9961
8948	9985	9962
8949	9986	9963
8950	9987	9965
8953	9988	9966
8954	9991	9969
8955	9994	9970
8958	9996	9971
8959	10000	9972
8960	10001	9973
8961	10005	9975
8962	10006	9977
8969	10010	9978
8970	10014	9980
8973	10015	9983
8977	10016	9984
8978	10017	9985
8979	10019	9986
8981	10021	9987
8982	10022	9991
8983	10025	9993
8985	10029	9994
8986	10031	9996
8988	10032	9999
8989	10039	10000
8991	10041	10005
8992	10046	10006
9000	10050	10011
9004	10057	10015
9009	10058	10017

9012	10059	10019
9013	10060	10021
9014	10062	10022
9018	10063	10023
9019	10068	10027
9020	10069	10028
9021	10070	10029
9024	10071	10030
9025	10072	10031
9029	10078	10032
9032	10079	10039
9034	10080	10043
9036	10082	10046
9037	10086	10050
9038	10093	10051
9039	10095	10057
9042	10096	10058
9045	10098	10059
9047	10099	10060
9048	10100	10062
9052	10101	10063
9056	10104	10066
9061	10105	10069
9062	10107	10070
9063	10109	10071
9064	10111	10073
9065	10113	10078
9066	10115	10079
9072	10117	10080
9077	10125	10082
9079	10126	10086
9080	10129	10093
9083	10130	10095
9088	10131	10096
9089	10133	10097
9091	10134	10098
9095	10135	10099
9097	10136	10100
9100	10138	10101
9106	10143	10104
9107	10144	10105
9113	10146	10107
9118	10147	10108
9119	10148	10113
9120	10149	10115
9125	10151	10120

9129	10152	10131
9133	10153	10133
9134	10154	10142
9135	10157	10144
9138	10160	10146
9139	10163	10147
9146	10168	10149
9147	10169	10151
9148	10170	10153
9150	10171	10154
9152	10172	10160
9154	10173	10161
9155	10174	10166
9156	10175	10168
9157	10176	10169
9158	10181	10171
9162	10183	10172
9165	10186	10173
9166	10188	10174
9167	10189	10175
9168	10190	10176
9169	10191	10178
9172	10192	10180
9173	10195	10183
9180	10197	10189
9181	10198	10191
9182	10200	10192
9183	10203	10193
9185	10204	10195
9186	10205	10196
9191	10210	10197
9192	10213	10198
9194	10217	10200
9195	10218	10203
9198	10219	10204
9201	10220	10205
9208	10221	10210
9209	10222	10217
9210	10224	10220
9211	10225	10221
9212	10227	10222
9213	10228	10224
9218	10230	10225
9219	10231	10227
9226	10232	10228
9227	10235	10229

9228	10243	10230
9230	10249	10231
9231	10252	10232
9233	10254	10235
9235	10256	10240
9238	10257	10243
9239	10262	10245
9243	10265	10246
9249	10267	10247
9250	10268	10252
9253	10271	10254
9261	10274	10255
9262	10277	10256
9263	10279	10257
9267	10280	10265
9268	10282	10266
9269	10285	10267
9273	10286	10268
9277	10288	10271
9278	10294	10273
9280	10295	10275
9281	10296	10277
9284	10297	10279
9285	10298	10280
9287	10299	10282
9290	10300	10285
9291	10301	10286
9292	10305	10288
9293	10307	10291
9294	10311	10294
9297	10313	10295
9298	10318	10296
9299	10320	10298
9300	10321	10299
9303	10323	10300
9306	10327	10301
9309	10328	10303
9312	10329	10304
9313	10330	10305
9316	10331	10307
9317	10332	10308
9319	10335	10311
9321	10336	10312
9323	10339	10316
9327	10346	10318
9328	10348	10321

9329	10351	10323
9331	10352	10326
9333	10354	10327
9334	10355	10328
9340	10356	10329
9342	10358	10330
9343	10372	10331
9349	10373	10332
9350	10378	10335
9353	10381	10336
9355	10385	10337
9359	10386	10339
9360	10388	10341
9361	10390	10346
9362	10393	10348
9364	10398	10351
9365	10399	10352
9367	10400	10354
9369	10402	10355
9375	10403	10356
9377	10407	10372
9380	10408	10373
9384	10409	10377
9386	10411	10378
9387	10412	10381
9389	10417	10385
9390	10418	10386
9396	10420	10388
9398	10421	10389
9400	10423	10390
9401	10425	10391
9406	10426	10393
9407	10428	10398
9409	10433	10399
9411	10434	10401
9412	10438	10402
9413	10439	10407
9416	10440	10408
9417	10442	10410
9418	10446	10411
9419	10447	10412
9420	10448	10418
9421	10449	10420
9422	10451	10423
9423	10453	10425
9424	10455	10426

9425	10458	10428
9429	10459	10433
9432	10461	10434
9435	10463	10435
9437	10466	10437
9438	10470	10438
9441	10476	10439
9444	10485	10440
9445	10486	10446
9447	10487	10447
9448	10489	10449
9456	10492	10450
9458	10494	10451
9466	10495	10453
9469	10498	10455
9470	10499	10459
9475	10500	10461
9478	10502	10462
9479	10503	10463
9480	10505	10465
9481	10507	10466
9482	10510	10467
9483	10511	10470
9485	10512	10476
9494	10513	10485
9495	10514	10489
9497	10515	10494
9498	10517	10495
9499	10520	10496
9500	10529	10497
9502	10532	10498
9504	10535	10499
9505	10537	10500
9507	10539	10502
9509	10541	10503
9512	10543	10505
9516	10544	10507
9517	10546	10509
9518	10556	10510
9522	10558	10511
9523	10560	10512
9524	10561	10513
9525	10562	10514
9530	10563	10515
9532	10567	10517
9534	10569	10518

9539	10570	10520
9540	10571	10531
9541	10573	10532
9542	10574	10534
9543	10575	10535
9544	10579	10537
9548	10583	10539
9549	10584	10540
9550	10585	10543
9553	10587	10544
9555	10589	10546
9557	10594	10556
9562	10597	10558
9566	10598	10559
9568	10599	10560
9569	10604	10561
9570	10606	10563
9572	10614	10565
9576	10615	10567
9577	10616	10570
9579	10617	10571
9584	10618	10572
9585	10621	10574
9586	10623	10575
9588	10626	10578
9590	10627	10579
9591	10629	10582
9599	10630	10584
9601	10634	10585
9604	10635	10587
9608	10636	10589
9609	10638	10590
9610	10639	10594
9611	10640	10597
9613	10648	10598
9614	10649	10599
9616	10650	10600
9622	10651	10604
9624	10654	10606
9626	10655	10613
9627	10657	10614
9632	10659	10615
9633	10661	10616
9634	10663	10617
9635	10664	10618
9636	10665	10620

9637	10671	10623
9639	10673	10626
9643	10675	10627
9644	10678	10629
9646	10679	10630
9653	10681	10634
9655	10682	10635
9659	10684	10637
9661	10685	10638
9663	10690	10640
9664	10697	10641
9665	10700	10643
9667	10704	10648
9668	10705	10650
9669	10707	10651
9671	10708	10652
9673	10710	10654
9675	10713	10659
9676	10714	10660
9677	10717	10661
9678	10718	10662
9679	10719	10664
9680	10720	10670
9681	10721	10671
9682	10723	10674
9683	10726	10675
9685	10728	10678
9688	10730	10679
9689	10731	10682
9693	10732	10684
9695	10733	10685
9697	10735	10686
9698	10737	10688
9701	10742	10690
9707	10743	10697
9708	10744	10699
9709	10745	10700
9711	10747	10704
9720	10748	10706
9724	10752	10707
9725	10755	10709
9726	10756	10710
9727	10759	10713
9729	10760	10717
9732	10761	10718
9733	10766	10719

9734	10768	10720
9735	10770	10721
9737	10771	10723
9746	10775	10726
9748	10777	10728
9749	10778	10730
9750	10779	10732
9751	10780	10733
9754	10783	10735
9755	10785	10736
9756	10786	10737
9759	10787	10742
9767	10789	10743
9772	10791	10744
9773	10792	10747
9774	10795	10748
9779	10798	10751
9783	10799	10752
9784	10801	10759
9785	10803	10760
9786	10804	10761
9787	10805	10762
9788	10808	10764
9789	10812	10765
9800	10813	10766
9806	10814	10768
9807	10816	10769
9811	10817	10770
9812	10818	10771
9813	10819	10772
9815	10820	10777
9816	10822	10778
9817	10823	10779
9818	10824	10780
9819	10826	10781
9820	10829	10782
9823	10831	10784
9824	10832	10785
9827	10839	10786
9828	10841	10787
9829	10843	10789
9833	10847	10792
9835	10850	10794
9836	10851	10795
9837	10859	10798
9839	10860	10799

9840	10861	10801
9844	10865	10803
9847	10867	10804
9851	10868	10805
9857	10873	10808
9858	10874	10812
9859	10875	10813
9862	10876	10814
9866	10878	10816
9867	10880	10817
9868	10882	10819
9870	10883	10820
9871	10884	10821
9873	10885	10822
9874	10886	10823
9875	10887	10824
9879	10892	10826
9880	10894	10829
9882	10900	10831
9883	10902	10832
9885	10904	10833
9886	10905	10837
9888	10907	10838
9889	10911	10839
9890	10915	10841
9893	10921	10847
9894	10923	10850
9896	10925	10851
9902	10926	10852
9906	10927	10856
9907	10934	10857
9908	10936	10858
9909	10938	10859
9917	10940	10860
9918	10944	10861
9919	10945	10862
9924	10949	10864
9928	10950	10865
9929	10951	10867
9931	10952	10868
9932	10955	10870
9933	10956	10872
9935	10959	10873
9936	10962	10874
9938	10963	10875
9939	10966	10877

9940	10968	10880
9942	10969	10881
9944	10971	10882
9946	10972	10884
9947	10977	10885
9950	10978	10886
9954	10979	10887
9955	10980	10892
9956	10981	10900
9957	10983	10902
9960	10984	10904
9961	10985	10907
9962	10986	10908
9967	10987	10911
9971	10989	10913
9972	10990	10914
9973	10994	10915
9974	10997	10921
9975	10999	10923
9978	11005	10924
9980	11008	10925
9984	11009	10926
9985	11010	10927
9986	11016	10934
9988	11017	10936
9991	11018	10937
9992	11022	10938
10000	11026	10939
10001	11028	10940
10005	11031	10941
10006	11032	10947
10007	11037	10948
10015	11040	10949
10016	11041	10950
10019	11043	10951
10021	11044	10955
10022	11048	10957
10024	11050	10959
10028	11051	10962
10029	11052	10966
10030	11053	10967
10031	11056	10969
10032	11059	10970
10036	11065	10971
10039	11066	10977
10041	11068	10978

10044	11071	10979
10046	11073	10980
10047	11075	10981
10048	11077	10983
10050	11078	10984
10054	11081	10986
10057	11082	10987
10059	11083	10990
10060	11086	10994
10062	11087	10996
10063	11088	10997
10069	11089	10998
10071	11091	10999
10072	11094	11003
10073	11095	11004
10074	11097	11008
10075	11098	11009
10077	11100	11010
10078	11101	11014
10085	11102	11016
10088	11103	11017
10092	11105	11018
10093	11106	11019
10095	11107	11020
10096	11108	11022
10099	11109	11024
10100	11111	11027
10101	11112	11028
10104	11113	11029
10105	11114	11031
10107	11117	11032
10113	11118	11033
10115	11120	11034
10117	11124	11035
10120	11125	11038
10121	11126	11040
10125	11127	11041
10128	11128	11042
10130	11130	11043
10131	11131	11044
10133	11132	11046
10134	11133	11049
10136	11134	11050
10138	11136	11051
10143	11138	11052
10144	11142	11054

10147	11145	11056
10148	11149	11057
10149	11150	11059
10151	11152	11062
10153	11154	11063
10154	11155	11065
10157	11156	11066
10158	11162	11067
10160	11164	11068
10163	11166	11069
10166	11168	11070
10168	11169	11071
10170	11171	11073
10172	11172	11074
10173	11173	11075
10174	11175	11076
10176	11176	11077
10178	11179	11078
10180	11183	11080
10181	11185	11082
10183	11187	11083
10184	11190	11088
10185	11192	11089
10189	11193	11091
10191	11194	11094
10192	11195	11096
10197	11197	11097
10198	11198	11098
10200	11201	11099
10203	11206	11100
10204	11208	11101
10205	11209	11102
10210	11212	11103
10212	11213	11105
10213	11216	11106
10214	11217	11107
10217	11225	11108
10220	11226	11110
10221	11231	11111
10222	11232	11112
10224	11246	11113
10226	11247	11114
10227	11248	11117
10228	11251	11118
10230	11252	11120
10231	11253	11124

10232	11254	11125
10233	11260	11126
10234	11262	11127
10235	11264	11128
10240	11269	11131
10246	11270	11132
10249	11272	11133
10252	11274	11134
10254	11275	11135
10257	11278	11136
10258	11280	11138
10259	11282	11142
10260	11285	11145
10262	11286	11146
10263	11287	11147
10265	11288	11149
10266	11291	11152
10268	11292	11155
10270	11293	11156
10271	11294	11157
10273	11296	11161
10274	11297	11162
10275	11300	11165
10277	11303	11166
10278	11310	11168
10279	11311	11169
10282	11317	11171
10284	11318	11172
10285	11320	11173
10286	11321	11174
10287	11322	11175
10288	11323	11176
10295	11324	11179
10296	11325	11180
10297	11327	11182
10298	11329	11183
10299	11330	11185
10300	11332	11187
10301	11335	11190
10304	11336	11191
10305	11337	11192
10309	11339	11194
10311	11340	11195
10313	11341	11197
10316	11343	11198
10318	11344	11199

10321	11345	11200
10323	11346	11201
10326	11347	11202
10327	11348	11206
10328	11350	11208
10329	11351	11209
10330	11352	11210
10335	11353	11212
10336	11354	11213
10339	11355	11215
10341	11358	11216
10345	11359	11218
10346	11365	11219
10351	11368	11221
10352	11373	11222
10354	11374	11223
10356	11376	11224
10358	11379	11225
10372	11380	11226
10373	11382	11227
10378	11384	11229
10381	11385	11237
10383	11386	11240
10385	11389	11247
10386	11396	11248
10388	11399	11249
10389	11401	11250
10390	11405	11251
10393	11406	11252
10396	11407	11253
10397	11409	11254
10398	11410	11258
10399	11412	11260
10400	11413	11262
10402	11414	11264
10403	11417	11268
10406	11419	11269
10407	11420	11270
10408	11421	11272
10409	11422	11274
10410	11423	11275
10411	11427	11276
10412	11428	11278
10417	11430	11279
10418	11431	11280
10420	11433	11282

10421	11437	11283
10423	11442	11285
10425	11447	11286
10428	11448	11287
10429	11449	11288
10430	11450	11290
10433	11452	11291
10434	11453	11292
10435	11455	11293
10437	11456	11294
10438	11461	11295
10439	11462	11300
10440	11467	11303
10442	11468	11304
10447	11469	11307
10449	11471	11308
10450	11475	11311
10451	11478	11316
10455	11479	11317
10458	11480	11321
10459	11484	11322
10461	11486	11323
10462	11487	11325
10463	11488	11327
10466	11490	11329
10470	11491	11330
10476	11493	11332
10483	11494	11335
10485	11496	11336
10486	11497	11337
10487	11498	11338
10488	11504	11339
10490	11505	11340
10492	11506	11341
10494	11507	11343
10495	11509	11344
10498	11510	11345
10499	11512	11346
10500	11513	11347
10503	11514	11350
10505	11517	11351
10506	11518	11352
10507	11519	11353
10508	11521	11354
10509	11522	11355
10511	11524	11358

10513	11527	11359
10515	11529	11361
10520	11531	11363
10521	11532	11366
10522	11533	11368
10529	11535	11369
10532	11536	11370
10535	11538	11373
10536	11541	11374
10538	11542	11376
10539	11543	11379
10541	11544	11380
10543	11545	11382
10546	11547	11383
10547	11548	11384
10548	11554	11385
10550	11557	11387
10551	11558	11388
10558	11560	11396
10559	11561	11398
10561	11563	11399
10562	11570	11400
10564	11572	11401
10565	11574	11402
10570	11575	11405
10571	11576	11406
10574	11578	11407
10575	11579	11409
10578	11580	11410
10579	11581	11412
10580	11583	11417
10583	11585	11418
10584	11587	11420
10585	11588	11421
10587	11591	11422
10594	11594	11423
10597	11595	11428
10598	11596	11430
10599	11597	11431
10600	11598	11434
10604	11601	11435
10606	11603	11437
10614	11604	11438
10615	11606	11445
10616	11608	11448
10618	11611	11449

10623	11615	11450
10626	11618	11452
10627	11623	11453
10629	11625	11454
10630	11627	11455
10634	11628	11456
10635	11630	11458
10638	11631	11461
10639	11632	11462
10643	11635	11467
10648	11636	11468
10649	11637	11470
10650	11639	11471
10651	11640	11475
10654	11641	11476
10655	11642	11478
10661	11644	11480
10666	11645	11482
10669	11646	11484
10671	11647	11486
10673	11648	11487
10675	11652	11490
10678	11653	11491
10681	11655	11494
10682	11656	11497
10684	11658	11498
10686	11659	11502
10689	11660	11504
10697	11661	11505
10698	11662	11509
10705	11663	11510
10708	11664	11512
10710	11665	11513
10714	11666	11514
10716	11668	11517
10717	11672	11519
10718	11673	11521
10719	11675	11522
10725	11676	11528
10728	11677	11529
10730	11682	11530
10731	11683	11531
10732	11684	11532
10733	11685	11533
10736	11689	11534
10737	11691	11535

10740	11692	11536
10742	11693	11538
10744	11694	11539
10745	11695	11541
10747	11697	11542
10754	11700	11543
10757	11701	11544
10758	11705	11545
10760	11706	11546
10762	11707	11547
10766	11708	11548
10768	11710	11554
10769	11711	11557
10770	11713	11558
10771	11716	11560
10773	11717	11561
10774	11718	11562
10776	11721	11563
10777	11722	11564
10778	11723	11568
10779	11724	11569
10781	11725	11572
10783	11726	11573
10785	11728	11574
10786	11729	11575
10787	11733	11576
10789	11736	11578
10795	11738	11579
10799	11742	11580
10801	11743	11581
10802	11746	11582
10804	11751	11583
10805	11759	11585
10808	11760	11586
10812	11761	11591
10814	11762	11594
10815	11764	11595
10816	11766	11596
10817	11767	11597
10818	11770	11598
10822	11771	11599
10823	11773	11600
10824	11776	11601
10829	11777	11603
10831	11779	11604
10832	11781	11606

10839	11783	11608
10841	11784	11609
10847	11786	11611
10848	11788	11615
10850	11789	11618
10861	11791	11622
10862	11792	11625
10868	11793	11626
10871	11795	11627
10873	11796	11628
10874	11802	11629
10875	11803	11631
10879	11804	11632
10880	11805	11634
10882	11807	11635
10884	11811	11636
10885	11814	11637
10886	11816	11639
10887	11818	11640
10889	11819	11641
10892	11821	11644
10900	11822	11645
10902	11824	11646
10904	11825	11647
10905	11827	11648
10911	11828	11651
10915	11829	11652
10921	11834	11653
10922	11835	11654
10923	11837	11655
10925	11840	11656
10926	11844	11658
10927	11845	11659
10930	11848	11660
10936	11849	11661
10945	11851	11662
10946	11852	11663
10950	11857	11664
10951	11858	11665
10952	11862	11666
10955	11864	11667
10956	11865	11668
10962	11868	11672
10964	11869	11673
10966	11870	11675
10969	11873	11676

10971	11876	11680
10973	11878	11682
10977	11881	11683
10978	11882	11684
10979	11884	11685
10980	11886	11689
10981	11890	11691
10983	11893	11692
10986	11901	11693
10987	11907	11694
10990	11908	11695
10993	11909	11696
10994	11910	11697
10996	11911	11698
11005	11912	11699
11008	11914	11700
11009	11916	11701
11010	11918	11703
11016	11924	11704
11017	11926	11705
11018	11927	11706
11019	11931	11707
11022	11933	11708
11026	11934	11709
11028	11935	11710
11031	11936	11711
11032	11938	11714
11038	11939	11716
11041	11942	11718
11043	11943	11721
11044	11945	11724
11050	11946	11725
11051	11947	11726
11052	11948	11728
11059	11949	11729
11062	11950	11733
11064	11953	11736
11065	11954	11738
11066	11955	11741
11067	11956	11742
11068	11958	11743
11070	11959	11746
11072	11960	11747
11073	11962	11748
11075	11963	11750
11077	11964	11751

11078	11966	11752
11080	11967	11753
11083	11968	11757
11087	11970	11758
11089	11971	11759
11091	11981	11760
11093	11983	11761
11094	11984	11762
11098	11986	11764
11099	11988	11766
11100	11989	11767
11101	11991	11768
11102	11992	11770
11103	11995	11771
11106	11996	11773
11108	11997	11774
11109	11998	11776
11110	11999	11777
11111	12000	11779
11112	12001	11780
11113	12002	11781
11114	12004	11782
11118	12005	11783
11121	12011	11786
11124	12012	11788
11126	12013	11790
11127	12014	11791
11128	12015	11792
11131	12017	11793
11132	12019	11795
11134	12020	11796
11137	12022	11798
11144	12023	11799
11145	12024	11804
11149	12025	11805
11150	12026	11806
11152	12028	11811
11153	12029	11814
11154	12030	11815
11155	12032	11816
11157	12034	11817
11161	12035	11818
11162	12038	11819
11164	12041	11821
11166	12042	11823
11168	12043	11824

11169	12045	11825
11171	12046	11829
11172	12047	11833
11174	12050	11834
11175	12053	11835
11183	12054	11837
11185	12055	11839
11190	12057	11840
11192	12062	11841
11194	12063	11844
11196	12064	11845
11198	12066	11849
11200	12070	11851
11201	12071	11855
11202	12075	11856
11203	12079	11857
11206	12080	11858
11209	12081	11862
11212	12087	11866
11213	12091	11868
11214	12092	11869
11216	12093	11870
11217	12101	11872
11225	12103	11873
11231	12109	11874
11232	12110	11876
11239	12111	11878
11242	12114	11881
11246	12116	11882
11247	12119	11884
11248	12120	11885
11251	12122	11889
11252	12123	11898
11254	12124	11906
11260	12127	11907
11261	12128	11908
11262	12129	11909
11264	12131	11910
11266	12132	11911
11269	12133	11912
11270	12134	11915
11271	12135	11916
11272	12136	11918
11274	12139	11924
11277	12144	11926
11280	12145	11927

11281	12153	11929
11282	12154	11931
11285	12155	11933
11287	12159	11934
11288	12160	11935
11291	12161	11937
11297	12162	11938
11303	12164	11942
11305	12165	11943
11310	12168	11944
11311	12172	11946
11314	12173	11947
11317	12174	11948
11319	12175	11950
11320	12177	11951
11321	12178	11953
11323	12179	11956
11326	12181	11958
11327	12184	11959
11330	12185	11960
11336	12187	11961
11337	12188	11962
11340	12189	11964
11341	12190	11966
11343	12192	11967
11344	12193	11968
11345	12197	11969
11346	12198	11971
11347	12199	11974
11348	12202	11984
11351	12203	11985
11352	12204	11986
11358	12205	11988
11359	12206	11989
11361	12207	11990
11364	12214	11991
11365	12215	11992
11366	12216	11995
11367	12217	11996
11369	12218	11997
11370	12220	11998
11373	12221	11999
11374	12222	12001
11375	12225	12002
11376	12226	12004
11379	12227	12005

11380	12230	12007
11382	12232	12010
11386	12233	12012
11389	12235	12013
11392	12237	12014
11396	12239	12015
11397	12243	12017
11401	12244	12019
11402	12245	12020
11405	12246	12022
11409	12248	12023
11410	12249	12024
11411	12252	12025
11412	12253	12026
11413	12254	12027
11417	12255	12028
11418	12256	12029
11420	12258	12030
11421	12259	12034
11423	12262	12036
11424	12263	12038
11425	12264	12040
11428	12266	12041
11429	12270	12043
11433	12272	12045
11437	12273	12046
11445	12274	12047
11447	12277	12049
11448	12279	12050
11452	12280	12051
11455	12281	12054
11456	12283	12055
11458	12284	12057
11467	12285	12062
11468	12286	12063
11471	12289	12064
11475	12290	12066
11476	12291	12068
11478	12292	12071
11480	12293	12075
11484	12298	12079
11488	12299	12080
11489	12300	12081
11490	12304	12091
11491	12305	12092
11496	12307	12101

11497	12308	12102
11498	12311	12103
11502	12312	12105
11504	12314	12109
11505	12319	12110
11506	12321	12111
11512	12322	12114
11514	12323	12116
11515	12324	12117
11516	12325	12119
11518	12326	12122
11519	12327	12123
11521	12330	12124
11522	12331	12125
11525	12332	12127
11529	12333	12128
11530	12335	12131
11531	12338	12132
11532	12340	12133
11533	12341	12134
11535	12342	12135
11538	12343	12136
11540	12344	12139
11542	12346	12144
11543	12347	12145
11544	12348	12146
11545	12351	12147
11548	12353	12148
11554	12354	12149
11556	12355	12150
11557	12356	12153
11558	12358	12154
11560	12360	12159
11561	12362	12162
11563	12363	12164
11570	12366	12165
11572	12372	12167
11575	12378	12168
11576	12383	12169
11578	12384	12172
11579	12385	12173
11580	12386	12174
11581	12389	12175
11582	12390	12177
11583	12392	12178
11585	12393	12181

11591	12394	12185
11594	12395	12187
11595	12396	12189
11596	12397	12190
11597	12398	12192
11599	12400	12193
11600	12401	12194
11601	12403	12196
11603	12406	12197
11604	12408	12198
11605	12411	12199
11606	12413	12200
11609	12414	12202
11617	12415	12205
11618	12416	12206
11619	12417	12210
11623	12418	12213
11624	12419	12215
11625	12420	12216
11627	12422	12217
11628	12423	12219
11630	12425	12220
11631	12428	12221
11632	12433	12222
11635	12436	12224
11636	12437	12229
11639	12439	12230
11640	12440	12231
11642	12442	12233
11643	12443	12234
11645	12444	12237
11646	12446	12239
11647	12450	12240
11648	12452	12242
11654	12455	12245
11655	12456	12246
11656	12457	12248
11660	12458	12249
11661	12459	12253
11662	12463	12254
11664	12464	12256
11668	12465	12258
11672	12466	12259
11673	12467	12260
11675	12470	12262
11676	12471	12263

11681	12472	12264
11682	12473	12265
11684	12475	12266
11689	12476	12268
11692	12477	12273
11695	12478	12274
11697	12482	12277
11698	12483	12279
11699	12484	12280
11700	12486	12281
11701	12487	12283
11704	12488	12284
11705	12490	12285
11706	12494	12286
11707	12495	12289
11708	12496	12290
11709	12497	12291
11710	12501	12292
11711	12502	12293
11713	12505	12298
11714	12506	12299
11721	12507	12304
11723	12511	12305
11724	12512	12306
11725	12513	12307
11726	12514	12308
11729	12515	12309
11733	12517	12312
11743	12518	12314
11746	12520	12316
11748	12521	12319
11750	12523	12321
11751	12524	12322
11752	12525	12323
11755	12526	12324
11756	12531	12325
11757	12537	12326
11761	12539	12327
11764	12541	12329
11765	12543	12330
11767	12544	12331
11769	12546	12332
11770	12547	12333
11771	12549	12335
11773	12550	12338
11774	12551	12340

11776	12552	12342
11777	12555	12343
11779	12556	12344
11781	12557	12345
11783	12558	12346
11784	12560	12348
11786	12561	12350
11788	12562	12352
11789	12563	12353
11790	12566	12354
11792	12569	12355
11793	12573	12356
11795	12574	12357
11799	12575	12358
11800	12576	12359
11804	12580	12360
11807	12581	12361
11809	12582	12362
11810	12583	12363
11814	12585	12364
11815	12587	12366
11816	12588	12371
11817	12591	12372
11818	12592	12377
11819	12593	12378
11821	12598	12383
11824	12599	12384
11831	12603	12385
11832	12605	12390
11833	12606	12392
11835	12607	12393
11837	12609	12394
11841	12610	12395
11842	12611	12396
11844	12612	12397
11845	12613	12399
11849	12616	12400
11851	12619	12401
11852	12621	12402
11855	12622	12406
11856	12623	12408
11857	12625	12409
11864	12629	12413
11865	12630	12415
11868	12631	12417
11869	12634	12419

11870	12635	12420
11873	12643	12422
11878	12647	12423
11879	12648	12425
11882	12649	12427
11884	12651	12428
11890	12654	12431
11896	12655	12433
11897	12659	12435
11898	12660	12436
11901	12662	12437
11907	12669	12439
11908	12670	12440
11909	12671	12441
11910	12672	12442
11911	12673	12443
11914	12674	12445
11918	12675	12446
11924	12678	12450
11926	12679	12455
11931	12681	12457
11932	12685	12458
11933	12686	12459
11935	12688	12463
11938	12689	12464
11942	12691	12465
11943	12692	12466
11946	12693	12470
11947	12694	12471
11950	12697	12472
11951	12700	12473
11953	12703	12477
11954	12704	12479
11955	12705	12480
11958	12709	12482
11959	12710	12483
11960	12712	12484
11962	12713	12486
11963	12714	12487
11964	12715	12488
11965	12716	12490
11966	12721	12491
11967	12722	12494
11968	12725	12495
11969	12731	12496
11971	12732	12497

11972	12733	12499
11980	12735	12501
11983	12736	12502
11984	12740	12505
11986	12741	12507
11987	12745	12510
11988	12746	12511
11989	12747	12512
11990	12748	12514
11991	12750	12515
11992	12751	12517
11995	12752	12518
11996	12754	12520
11997	12756	12521
11998	12758	12522
12004	12760	12523
12005	12761	12524
12007	12762	12526
12012	12763	12530
12013	12771	12536
12014	12773	12537
12015	12774	12539
12017	12776	12540
12019	12777	12541
12020	12778	12543
12022	12779	12544
12023	12786	12545
12025	12788	12546
12028	12789	12547
12029	12790	12549
12030	12793	12551
12032	12794	12552
12036	12795	12553
12037	12796	12555
12039	12797	12556
12042	12798	12557
12043	12800	12558
12045	12801	12559
12046	12802	12560
12047	12804	12561
12049	12805	12562
12050	12808	12563
12051	12809	12564
12052	12810	12566
12055	12813	12569
12063	12815	12570

12068	12817	12573
12070	12818	12574
12071	12820	12575
12075	12821	12576
12077	12825	12578
12079	12830	12583
12080	12831	12585
12087	12833	12586
12091	12834	12587
12100	12835	12588
12101	12837	12591
12103	12839	12592
12106	12840	12593
12110	12841	12595
12111	12842	12598
12116	12843	12599
12119	12845	12600
12122	12847	12604
12123	12850	12605
12124	12851	12606
12126	12852	12607
12127	12853	12610
12128	12854	12611
12132	12855	12612
12133	12858	12615
12134	12859	12618
12135	12860	12619
12136	12861	12620
12139	12863	12621
12140	12865	12622
12144	12868	12623
12145	12869	12627
12147	12870	12630
12151	12875	12631
12154	12877	12637
12155	12879	12639
12157	12883	12643
12161	12884	12649
12162	12887	12651
12164	12888	12654
12167	12891	12655
12169	12895	12657
12172	12897	12659
12173	12898	12662
12175	12899	12663
12177	12900	12669

12184	12903	12670
12185	12904	12671
12186	12905	12672
12187	12909	12673
12189	12910	12674
12190	12911	12675
12192	12914	12678
12193	12920	12679
12194	12921	12682
12196	12923	12684
12197	12925	12685
12198	12927	12686
12199	12928	12687
12202	12932	12690
12205	12933	12691
12206	12936	12692
12207	12937	12693
12210	12938	12694
12213	12945	12696
12216	12948	12697
12217	12949	12700
12218	12950	12701
12221	12951	12703
12222	12953	12704
12227	12954	12705
12232	12955	12706
12233	12956	12708
12235	12958	12709
12237	12960	12710
12239	12961	12713
12240	12962	12714
12242	12966	12715
12244	12967	12716
12245	12969	12719
12247	12970	12720
12248	12971	12721
12249	12974	12722
12253	12977	12723
12254	12978	12724
12258	12979	12725
12260	12981	12728
12262	12982	12730
12263	12983	12731
12264	12984	12732
12266	12985	12733
12269	12987	12734

12270	12988	12735
12272	12989	12736
12273	12991	12738
12274	12993	12739
12276	12996	12740
12277	12997	12741
12280	12998	12745
12281	13004	12746
12283	13010	12747
12284	13011	12750
12285	13013	12751
12286	13014	12752
12287	13015	12754
12290	13018	12755
12292	13019	12756
12298	13021	12758
12303	13022	12761
12304	13023	12762
12305	13031	12763
12306	13032	12766
12307	13033	12774
12309	13034	12775
12311	13036	12776
12312	13038	12777
12314	13042	12778
12319	13043	12779
12320	13044	12783
12322	13045	12786
12323	13046	12790
12324	13049	12793
12325	13051	12794
12326	13054	12795
12330	13056	12796
12333	13057	12798
12335	13060	12800
12338	13061	12801
12341	13064	12802
12342	13066	12804
12344	13067	12805
12347	13068	12806
12348	13070	12808
12351	13073	12809
12352	13079	12810
12354	13081	12812
12355	13082	12814
12358	13084	12815

12359	13085	12817
12360	13086	12818
12361	13087	12820
12362	13088	12821
12363	13089	12823
12364	13090	12826
12367	13091	12831
12369	13092	12832
12371	13093	12833
12372	13094	12835
12373	13095	12837
12378	13096	12839
12379	13097	12840
12383	13098	12841
12384	13099	12842
12385	13101	12843
12386	13104	12845
12387	13105	12846
12388	13109	12847
12392	13110	12850
12393	13112	12851
12394	13113	12853
12395	13115	12854
12396	13116	12858
12397	13118	12859
12400	13121	12860
12408	13123	12861
12413	13125	12863
12415	13128	12865
12416	13130	12869
12417	13131	12871
12418	13132	12873
12419	13133	12875
12420	13135	12876
12421	13136	12877
12422	13137	12879
12423	13140	12882
12424	13141	12884
12427	13148	12885
12428	13149	12887
12429	13150	12891
12433	13151	12898
12436	13152	12899
12437	13153	12903
12439	13155	12904
12440	13156	12905

12442	13157	12909
12444	13158	12910
12446	13159	12911
12448	13160	12914
12449	13161	12920
12452	13162	12921
12454	13165	12926
12455	13166	12927
12456	13167	12928
12457	13168	12929
12458	13169	12932
12459	13170	12933
12463	13174	12934
12465	13175	12935
12466	13176	12936
12469	13177	12937
12470	13178	12938
12471	13181	12940
12472	13183	12948
12473	13184	12949
12475	13185	12950
12476	13188	12951
12477	13189	12952
12482	13190	12953
12483	13192	12954
12484	13195	12955
12485	13196	12956
12487	13197	12959
12488	13198	12960
12490	13200	12961
12495	13201	12962
12497	13205	12965
12505	13206	12966
12506	13207	12967
12508	13208	12968
12512	13209	12970
12514	13210	12974
12515	13211	12977
12518	13212	12978
12520	13213	12979
12521	13215	12980
12522	13217	12981
12524	13219	12982
12525	13224	12984
12526	13227	12985
12535	13228	12987

12537	13230	12989
12539	13234	12998
12541	13235	12999
12542	13236	13004
12543	13237	13007
12544	13238	13008
12545	13239	13011
12549	13240	13012
12550	13242	13014
12551	13244	13015
12552	13245	13018
12553	13246	13019
12556	13247	13022
12557	13248	13028
12559	13249	13031
12561	13251	13032
12563	13252	13033
12564	13254	13034
12570	13255	13036
12573	13260	13037
12574	13261	13038
12575	13262	13040
12578	13263	13042
12580	13264	13043
12581	13266	13044
12582	13270	13046
12583	13273	13049
12587	13279	13051
12588	13280	13055
12591	13281	13057
12592	13282	13060
12593	13283	13063
12596	13284	13065
12599	13287	13067
12602	13288	13068
12603	13289	13070
12605	13291	13078
12608	13292	13079
12614	13293	13080
12616	13295	13082
12618	13296	13084
12619	13300	13086
12620	13302	13087
12621	13303	13088
12623	13304	13090
12630	13305	13091

12631	13306	13092
12636	13307	13093
12637	13308	13094
12641	13309	13095
12644	13311	13096
12647	13312	13097
12648	13313	13098
12653	13315	13099
12656	13316	13100
12660	13317	13104
12661	13318	13105
12662	13319	13107
12668	13320	13110
12670	13322	13111
12672	13325	13112
12673	13326	13113
12674	13333	13115
12675	13334	13116
12677	13335	13122
12679	13336	13125
12684	13340	13126
12686	13341	13128
12687	13345	13129
12691	13348	13130
12692	13350	13131
12693	13351	13132
12694	13352	13133
12695	13353	13134
12697	13354	13135
12700	13355	13141
12701	13356	13145
12703	13357	13148
12704	13361	13149
12705	13362	13150
12707	13363	13151
12708	13364	13152
12709	13365	13155
12713	13366	13156
12714	13368	13157
12720	13369	13158
12722	13370	13159
12725	13371	13160
12730	13372	13161
12731	13373	13162
12733	13374	13163
12736	13375	13168

12741	13378	13169
12743	13381	13170
12750	13382	13174
12751	13383	13175
12752	13385	13176
12753	13389	13177
12756	13392	13178
12758	13393	13180
12760	13394	13183
12761	13396	13184
12762	13398	13185
12763	13399	13187
12764	13400	13188
12765	13401	13189
12770	13402	13190
12771	13406	13191
12772	13407	13192
12774	13408	13200
12776	13410	13201
12778	13411	13206
12779	13412	13207
12781	13413	13208
12783	13414	13209
12786	13416	13212
12787	13417	13213
12788	13418	13215
12789	13425	13217
12790	13427	13219
12791	13428	13224
12793	13429	13225
12794	13431	13227
12795	13433	13228
12796	13434	13229
12797	13436	13231
12799	13440	13232
12801	13444	13234
12802	13446	13236
12804	13455	13237
12805	13457	13239
12808	13458	13240
12809	13459	13241
12810	13460	13242
12811	13464	13245
12813	13465	13246
12814	13466	13247
12815	13468	13248

12817	13470	13251
12818	13471	13252
12821	13472	13253
12822	13473	13260
12825	13475	13261
12828	13476	13262
12829	13477	13263
12831	13478	13264
12833	13479	13266
12835	13480	13267
12836	13484	13270
12837	13485	13273
12838	13487	13275
12839	13488	13280
12840	13489	13281
12841	13491	13282
12842	13495	13283
12843	13496	13284
12844	13498	13286
12845	13499	13287
12846	13500	13288
12847	13501	13289
12849	13503	13291
12850	13505	13292
12851	13507	13295
12853	13509	13296
12854	13510	13300
12855	13512	13301
12858	13513	13302
12859	13514	13303
12860	13517	13305
12863	13518	13306
12868	13519	13307
12869	13520	13308
12870	13521	13309
12873	13522	13311
12874	13523	13312
12875	13524	13313
12877	13525	13317
12879	13527	13318
12884	13530	13319
12887	13535	13320
12889	13536	13322
12890	13537	13323
12891	13539	13325
12897	13543	13326

12898	13545	13328
12899	13546	13330
12900	13547	13332
12904	13548	13335
12905	13549	13336
12907	13550	13338
12909	13553	13339
12910	13554	13345
12911	13555	13346
12912	13556	13347
12914	13560	13349
12915	13561	13350
12916	13562	13351
12920	13563	13352
12921	13565	13353
12922	13566	13354
12923	13568	13356
12927	13570	13357
12928	13571	13362
12931	13572	13363
12932	13573	13364
12933	13575	13366
12940	13576	13367
12941	13577	13368
12942	13578	13369
12945	13584	13370
12948	13585	13371
12949	13589	13372
12950	13591	13373
12951	13592	13375
12953	13594	13378
12954	13595	13381
12956	13596	13382
12958	13597	13383
12960	13598	13385
12961	13599	13389
12963	13600	13390
12965	13602	13391
12966	13604	13392
12967	13606	13394
12969	13607	13396
12970	13608	13398
12974	13612	13399
12976	13613	13400
12977	13614	13402
12978	13616	13406

12979	13617	13407
12980	13618	13408
12982	13619	13411
12983	13626	13412
12985	13627	13413
12987	13631	13414
12988	13632	13415
12989	13633	13416
12992	13636	13417
12996	13639	13418
12997	13642	13424
12998	13643	13428
13000	13646	13430
13001	13647	13431
13002	13648	13433
13003	13651	13434
13006	13652	13435
13012	13656	13438
13015	13657	13439
13016	13658	13440
13018	13660	13442
13019	13662	13444
13020	13663	13445
13021	13665	13454
13022	13666	13457
13023	13668	13458
13030	13670	13460
13031	13671	13461
13032	13672	13464
13033	13673	13465
13035	13677	13470
13038	13680	13471
13039	13681	13472
13042	13683	13473
13043	13684	13475
13051	13685	13476
13054	13686	13478
13055	13687	13479
13056	13688	13484
13057	13689	13485
13061	13690	13487
13062	13693	13488
13063	13695	13489
13066	13697	13491
13067	13698	13492
13068	13700	13495

13070	13703	13496
13072	13708	13498
13078	13712	13499
13079	13713	13500
13081	13719	13501
13082	13721	13503
13084	13722	13504
13085	13726	13505
13086	13727	13506
13087	13730	13507
13088	13732	13509
13090	13739	13510
13091	13740	13512
13093	13741	13513
13095	13743	13514
13096	13744	13517
13097	13745	13518
13098	13749	13519
13099	13752	13520
13104	13753	13521
13105	13754	13522
13106	13755	13523
13107	13759	13524
13109	13761	13525
13110	13762	13526
13112	13763	13527
13113	13764	13530
13115	13765	13535
13116	13771	13536
13118	13772	13537
13124	13777	13543
13125	13778	13545
13126	13779	13546
13127	13780	13548
13128	13782	13549
13129	13785	13553
13130	13786	13555
13132	13788	13560
13133	13789	13565
13134	13790	13566
13135	13792	13571
13136	13794	13577
13137	13795	13578
13138	13796	13579
13141	13797	13585
13142	13798	13589

13143	13799	13590
13147	13800	13591
13149	13802	13592
13150	13803	13595
13152	13804	13596
13155	13809	13597
13156	13811	13598
13157	13812	13599
13159	13813	13600
13161	13816	13602
13162	13817	13604
13163	13819	13606
13165	13820	13607
13166	13822	13612
13167	13823	13613
13168	13824	13614
13169	13825	13616
13170	13827	13617
13171	13828	13618
13175	13830	13619
13176	13831	13620
13178	13832	13626
13179	13836	13628
13183	13838	13632
13184	13839	13633
13185	13841	13636
13186	13842	13637
13188	13843	13639
13189	13845	13642
13190	13847	13643
13191	13848	13644
13192	13849	13646
13194	13851	13647
13195	13852	13648
13199	13853	13651
13200	13854	13652
13201	13856	13653
13202	13859	13654
13205	13860	13655
13207	13861	13657
13208	13862	13658
13209	13864	13659
13213	13867	13660
13215	13869	13666
13216	13873	13669
13217	13874	13670

13218	13878	13671
13219	13879	13672
13225	13880	13674
13226	13881	13677
13227	13882	13679
13228	13883	13681
13232	13884	13682
13234	13888	13683
13238	13889	13684
13239	13890	13685
13240	13892	13686
13242	13893	13687
13246	13894	13688
13247	13895	13689
13248	13896	13693
13251	13898	13698
13252	13899	13700
13254	13904	13701
13256	13907	13703
13257	13909	13704
13258	13912	13705
13260	13915	13707
13262	13918	13708
13263	13920	13712
13264	13923	13716
13266	13925	13719
13269	13926	13721
13272	13927	13722
13273	13928	13726
13274	13930	13727
13277	13931	13730
13279	13936	13733
13280	13939	13739
13281	13940	13740
13282	13944	13741
13283	13945	13743
13284	13946	13746
13285	13947	13750
13287	13949	13752
13288	13953	13754
13289	13954	13755
13291	13955	13756
13296	13956	13757
13297	13957	13758
13300	13958	13759
13301	13959	13761

13302	13961	13763
13303	13962	13764
13304	13965	13765
13305	13966	13767
13306	13967	13769
13308	13969	13772
13309	13970	13775
13310	13971	13786
13312	13972	13788
13313	13973	13789
13314	13974	13790
13317	13975	13792
13319	13976	13795
13320	13977	13797
13321	13978	13799
13322	13979	13800
13325	13984	13801
13326	13987	13802
13331	13988	13803
13335	13989	13809
13336	13992	13810
13340	13993	13811
13342	13994	13812
13344	13997	13813
13346	13998	13814
13347	14001	13816
13348	14002	13817
13352	14003	13819
13353	14009	13822
13355	14010	13823
13356	14011	13824
13357	14013	13825
13359	14014	13827
13362	14015	13828
13363	14016	13831
13364	14018	13832
13366	14020	13835
13368	14021	13836
13369	14025	13837
13370	14027	13838
13371	14030	13839
13372	14033	13841
13373	14037	13842
13374	14038	13845
13375	14041	13847
13376	14042	13848

13378	14043	13849
13382	14044	13851
13383	14046	13852
13385	14048	13854
13387	14049	13856
13389	14050	13858
13392	14052	13859
13393	14057	13860
13394	14060	13861
13395	14061	13862
13397	14062	13864
13398	14063	13867
13399	14066	13873
13400	14069	13878
13401	14070	13879
13402	14071	13880
13406	14074	13881
13409	14076	13882
13410	14079	13883
13411	14080	13884
13412	14081	13885
13414	14086	13886
13415	14092	13888
13416	14093	13889
13417	14095	13890
13418	14097	13891
13421	14098	13892
13425	14099	13893
13426	14101	13895
13428	14102	13896
13430	14103	13899
13431	14104	13903
13433	14105	13904
13434	14106	13906
13435	14107	13907
13436	14110	13910
13437	14111	13915
13438	14113	13916
13439	14116	13918
13440	14117	13920
13442	14118	13922
13444	14123	13923
13446	14124	13925
13447	14128	13926
13448	14129	13927
13451	14130	13930

13453	14131	13934
13459	14132	13936
13464	14133	13939
13466	14134	13940
13468	14135	13941
13469	14136	13946
13470	14137	13949
13471	14138	13953
13473	14139	13954
13476	14140	13955
13477	14141	13957
13478	14143	13958
13479	14145	13959
13480	14146	13960
13483	14147	13961
13486	14148	13962
13488	14149	13965
13489	14151	13966
13490	14152	13967
13495	14154	13969
13497	14156	13970
13498	14158	13972
13499	14159	13973
13500	14160	13974
13501	14161	13976
13503	14162	13977
13504	14163	13979
13505	14165	13985
13509	14170	13987
13510	14171	13989
13511	14172	13992
13512	14173	13993
13513	14174	13995
13514	14179	13997
13515	14183	13998
13517	14184	14001
13519	14186	14003
13520	14188	14005
13521	14193	14009
13522	14194	14010
13523	14195	14011
13524	14197	14012
13525	14198	14013
13526	14199	14014
13527	14201	14016
13528	14204	14018

13529	14205	14020
13531	14208	14021
13535	14210	14025
13536	14212	14027
13537	14215	14031
13540	14216	14033
13543	14218	14034
13545	14219	14035
13546	14220	14037
13547	14224	14038
13548	14225	14041
13549	14226	14043
13551	14227	14044
13553	14229	14046
13554	14230	14047
13555	14234	14048
13556	14236	14049
13559	14237	14052
13560	14238	14056
13561	14240	14058
13562	14242	14060
13563	14244	14061
13565	14246	14062
13566	14247	14063
13568	14250	14066
13569	14251	14069
13570	14252	14070
13572	14256	14071
13573	14258	14074
13575	14259	14075
13576	14261	14076
13577	14262	14079
13578	14263	14080
13579	14264	14081
13581	14270	14088
13584	14271	14091
13585	14275	14092
13586	14276	14093
13588	14278	14095
13589	14281	14097
13591	14289	14098
13592	14292	14101
13594	14293	14102
13596	14294	14103
13599	14295	14104
13600	14296	14105

13603	14298	14106
13604	14301	14107
13606	14302	14110
13607	14304	14111
13609	14307	14113
13612	14308	14118
13613	14310	14123
13614	14311	14124
13615	14312	14127
13616	14313	14128
13617	14314	14130
13618	14317	14131
13619	14318	14132
13620	14319	14133
13625	14322	14134
13626	14324	14136
13627	14326	14138
13630	14329	14139
13632	14330	14140
13633	14331	14141
13634	14332	14145
13635	14333	14146
13639	14334	14147
13641	14335	14148
13642	14336	14149
13644	14337	14151
13645	14340	14152
13647	14341	14154
13648	14345	14158
13651	14346	14159
13653	14347	14160
13655	14350	14162
13656	14351	14163
13657	14352	14165
13658	14357	14170
13659	14358	14171
13660	14359	14172
13662	14360	14173
13663	14362	14177
13664	14363	14178
13668	14364	14179
13669	14368	14184
13670	14369	14186
13671	14374	14191
13672	14377	14193
13675	14378	14194

13676	14379	14195
13677	14381	14197
13680	14383	14198
13681	14385	14203
13683	14387	14205
13684	14393	14210
13686	14394	14212
13687	14395	14215
13688	14396	14217
13690	14399	14218
13692	14400	14220
13693	14402	14221
13694	14403	14222
13697	14405	14224
13698	14406	14226
13700	14408	14227
13702	14409	14229
13703	14411	14230
13704	14412	14232
13706	14416	14234
13708	14419	14236
13711	14420	14237
13713	14421	14238
13714	14423	14244
13716	14424	14246
13721	14425	14247
13722	14426	14248
13723	14427	14250
13726	14428	14251
13730	14429	14252
13734	14431	14253
13738	14433	14254
13739	14434	14256
13740	14436	14258
13741	14437	14259
13742	14439	14260
13743	14440	14261
13744	14443	14262
13745	14444	14264
13747	14450	14270
13748	14451	14272
13749	14454	14274
13752	14455	14275
13753	14456	14278
13755	14457	14281
13756	14459	14289

13759	14464	14292
13762	14465	14293
13763	14468	14294
13764	14471	14296
13765	14472	14297
13766	14473	14300
13768	14474	14301
13769	14479	14302
13771	14482	14305
13772	14484	14308
13774	14486	14310
13776	14490	14311
13778	14491	14312
13781	14505	14313
13782	14507	14314
13784	14508	14315
13785	14510	14317
13786	14511	14318
13788	14515	14320
13789	14516	14322
13790	14517	14324
13791	14518	14326
13792	14521	14331
13795	14522	14332
13796	14523	14333
13797	14524	14334
13798	14526	14335
13799	14527	14336
13800	14528	14337
13803	14529	14340
13804	14530	14341
13807	14531	14343
13808	14532	14346
13809	14533	14347
13810	14534	14348
13811	14535	14350
13812	14536	14351
13813	14539	14352
13815	14541	14357
13816	14543	14358
13817	14544	14359
13820	14545	14360
13822	14547	14362
13823	14548	14363
13824	14549	14364
13827	14550	14368

13828	14551	14369
13830	14552	14374
13831	14556	14377
13832	14557	14378
13835	14558	14379
13836	14559	14381
13837	14560	14383
13838	14561	14384
13839	14565	14385
13841	14567	14388
13842	14568	14393
13845	14573	14394
13846	14574	14395
13848	14579	14396
13849	14580	14400
13851	14583	14402
13854	14585	14403
13856	14588	14404
13857	14589	14405
13859	14590	14406
13860	14591	14408
13861	14593	14409
13862	14595	14411
13866	14596	14412
13867	14601	14414
13868	14602	14420
13869	14603	14423
13870	14607	14424
13871	14613	14425
13873	14614	14426
13876	14615	14427
13878	14617	14429
13879	14618	14430
13881	14619	14431
13883	14621	14433
13884	14622	14434
13885	14626	14437
13887	14628	14439
13888	14629	14440
13889	14631	14441
13892	14633	14443
13893	14634	14444
13894	14637	14445
13895	14638	14447
13897	14640	14450
13898	14641	14451

13899	14642	14454
13901	14643	14455
13903	14644	14456
13906	14645	14457
13907	14646	14458
13908	14648	14459
13909	14649	14460
13912	14650	14464
13914	14651	14465
13915	14656	14468
13917	14657	14470
13918	14659	14471
13919	14661	14472
13920	14663	14473
13921	14665	14474
13922	14667	14475
13923	14668	14478
13924	14669	14479
13925	14673	14482
13926	14674	14486
13927	14676	14488
13929	14677	14491
13930	14678	14493
13931	14679	14494
13934	14681	14502
13936	14683	14505
13939	14684	14507
13940	14686	14508
13944	14687	14509
13945	14688	14510
13947	14692	14515
13948	14693	14517
13949	14694	14518
13953	14695	14521
13954	14697	14522
13958	14700	14523
13959	14701	14524
13960	14702	14526
13961	14703	14527
13962	14707	14528
13965	14709	14529
13966	14710	14534
13969	14714	14536
13970	14715	14539
13971	14716	14540
13972	14718	14541

13973	14720	14543
13974	14723	14544
13975	14724	14545
13976	14725	14548
13977	14726	14549
13979	14727	14550
13980	14729	14551
13982	14730	14552
13984	14731	14557
13986	14733	14558
13987	14734	14559
13989	14737	14560
13992	14738	14561
13993	14739	14566
14001	14742	14567
14002	14743	14568
14003	14744	14569
14005	14746	14573
14009	14747	14574
14010	14748	14577
14011	14749	14579
14012	14754	14580
14016	14755	14583
14019	14757	14585
14020	14759	14589
14021	14763	14590
14030	14764	14591
14032	14765	14593
14033	14766	14595
14035	14767	14596
14037	14768	14600
14038	14771	14601
14039	14773	14602
14041	14774	14603
14042	14777	14607
14043	14778	14613
14044	14779	14615
14046	14780	14617
14047	14781	14618
14048	14783	14619
14049	14784	14621
14056	14787	14622
14059	14788	14624
14060	14789	14625
14061	14790	14626
14062	14795	14628

14063	14796	14629
14065	14800	14631
14066	14801	14633
14069	14802	14634
14070	14803	14637
14071	14804	14638
14073	14805	14640
14075	14808	14641
14076	14809	14642
14081	14811	14644
14086	14813	14645
14087	14816	14646
14090	14819	14648
14092	14820	14649
14093	14821	14650
14097	14822	14651
14098	14823	14654
14099	14825	14657
14101	14827	14659
14102	14828	14661
14103	14830	14663
14104	14833	14665
14106	14834	14667
14107	14835	14668
14108	14836	14669
14109	14839	14676
14110	14840	14677
14114	14843	14678
14117	14845	14679
14120	14848	14681
14121	14849	14683
14123	14850	14684
14124	14852	14686
14125	14853	14687
14126	14855	14688
14129	14858	14691
14130	14859	14692
14131	14862	14693
14132	14863	14695
14133	14867	14697
14134	14868	14700
14136	14870	14702
14137	14874	14704
14138	14875	14705
14139	14877	14709
14140	14878	14711

14141	14879	14714
14145	14882	14715
14147	14883	14716
14148	14884	14719
14149	14886	14720
14150	14887	14723
14151	14892	14724
14152	14893	14725
14154	14897	14726
14156	14898	14727
14157	14900	14729
14158	14905	14731
14159	14906	14733
14161	14908	14734
14162	14911	14737
14165	14918	14739
14170	14924	14743
14172	14925	14744
14175	14926	14747
14177	14927	14754
14179	14928	14755
14180	14931	14757
14181	14932	14765
14182	14933	14766
14183	14935	14767
14188	14936	14768
14189	14938	14771
14192	14941	14773
14194	14943	14774
14195	14945	14778
14196	14946	14781
14198	14948	14783
14201	14949	14787
14202	14956	14790
14205	14958	14793
14207	14961	14795
14208	14962	14796
14209	14963	14800
14210	14966	14801
14211	14968	14802
14212	14970	14803
14215	14973	14804
14216	14975	14805
14217	14976	14807
14218	14977	14809
14219	14978	14810

14220	14980	14811
14222	14981	14816
14224	14985	14820
14225	14989	14821
14226	14990	14822
14227	14991	14823
14228	14992	14825
14230	14994	14826
14234	14997	14827
14237	14998	14828
14238	14999	14829
14239	15001	14830
14241	15002	14833
14243	15003	14835
14244	15005	14836
14246	15006	14839
14247	15007	14840
14248	15009	14842
14250	15014	14843
14251	15017	14845
14252	15018	14848
14254	15019	14850
14257	15020	14852
14258	15022	14853
14259	15023	14855
14261	15024	14858
14264	15025	14859
14265	15026	14862
14268	15032	14863
14270	15033	14864
14271	15034	14865
14272	15036	14866
14273	15037	14867
14275	15040	14868
14277	15041	14873
14278	15042	14874
14281	15043	14875
14284	15046	14877
14289	15049	14878
14290	15053	14879
14292	15054	14882
14293	15056	14883
14294	15059	14884
14298	15060	14886
14299	15061	14892
14301	15063	14893

14302	15064	14900
14305	15067	14902
14306	15068	14905
14307	15075	14906
14308	15079	14908
14309	15080	14911
14310	15081	14912
14312	15082	14917
14313	15084	14918
14314	15087	14919
14315	15089	14921
14317	15090	14924
14322	15091	14925
14323	15093	14926
14324	15096	14927
14328	15097	14928
14329	15098	14932
14331	15099	14933
14332	15101	14935
14333	15103	14936
14334	15108	14938
14335	15110	14945
14337	15111	14946
14339	15114	14949
14340	15116	14961
14341	15119	14962
14343	15121	14963
14347	15123	14968
14348	15124	14969
14349	15127	14972
14350	15128	14973
14351	15129	14978
14352	15131	14980
14353	15132	14981
14354	15134	14984
14355	15136	14985
14356	15138	14990
14357	15139	14991
14359	15144	14992
14360	15145	14994
14362	15147	14997
14363	15149	14999
14364	15150	15001
14365	15152	15004
14366	15154	15005
14367	15156	15007

14369	15158	15010
14370	15159	15012
14373	15160	15014
14375	15162	15015
14378	15163	15019
14379	15168	15020
14380	15172	15022
14381	15174	15024
14383	15176	15025
14384	15180	15026
14386	15183	15027
14387	15184	15030
14389	15185	15031
14391	15187	15032
14394	15190	15034
14396	15192	15035
14398	15194	15036
14399	15197	15037
14400	15199	15040
14401	15200	15041
14402	15201	15042
14404	15202	15046
14406	15203	15047
14408	15204	15053
14409	15205	15055
14411	15208	15058
14412	15210	15060
14413	15211	15061
14414	15212	15062
14416	15216	15067
14419	15219	15068
14420	15220	15073
14421	15221	15074
14422	15222	15075
14423	15223	15079
14424	15225	15080
14425	15227	15082
14426	15239	15083
14427	15240	15084
14428	15241	15087
14429	15247	15088
14431	15250	15089
14433	15251	15090
14434	15252	15091
14437	15255	15093
14440	15258	15096

14441	15264	15097
14442	15265	15099
14443	15268	15101
14450	15269	15103
14451	15270	15104
14454	15271	15110
14456	15272	15111
14457	15277	15112
14458	15278	15114
14460	15279	15116
14461	15280	15119
14462	15284	15121
14465	15286	15124
14466	15288	15128
14468	15293	15129
14471	15294	15131
14472	15297	15132
14473	15299	15134
14474	15301	15136
14475	15302	15138
14476	15305	15143
14478	15306	15144
14479	15307	15145
14482	15308	15147
14483	15309	15149
14484	15310	15151
14485	15316	15152
14486	15317	15154
14487	15320	15158
14488	15322	15159
14489	15325	15162
14490	15326	15163
14491	15328	15164
14493	15329	15165
14494	15330	15169
14495	15331	15174
14496	15337	15180
14501	15338	15183
14502	15340	15185
14504	15341	15187
14505	15343	15188
14507	15344	15194
14508	15345	15195
14510	15346	15197
14511	15348	15199
14512	15350	15200

14513	15351	15202
14514	15355	15203
14516	15358	15205
14517	15359	15208
14518	15362	15209
14520	15363	15210
14521	15364	15211
14523	15365	15214
14524	15366	15219
14527	15367	15221
14529	15368	15222
14531	15370	15223
14533	15373	15225
14534	15374	15241
14535	15378	15247
14536	15379	15250
14541	15382	15251
14543	15384	15252
14544	15385	15258
14547	15386	15265
14549	15387	15267
14550	15388	15269
14551	15391	15271
14557	15393	15277
14558	15395	15279
14559	15396	15281
14560	15398	15288
14561	15399	15291
14562	15400	15294
14565	15403	15297
14566	15404	15301
14568	15405	15302
14569	15409	15305
14573	15411	15306
14574	15412	15307
14575	15413	15308
14578	15415	15309
14579	15416	15315
14580	15417	15316
14582	15420	15319
14583	15424	15320
14585	15428	15323
14586	15430	15325
14588	15433	15328
14589	15434	15329
14591	15435	15330

14593	15436	15331
14594	15438	15337
14595	15440	15340
14596	15442	15341
14598	15445	15342
14601	15446	15344
14602	15447	15345
14603	15448	15346
14607	15451	15350
14608	15452	15351
14613	15453	15355
14617	15457	15358
14618	15458	15364
14619	15459	15366
14620	15460	15370
14621	15467	15373
14622	15500	15374
14625	15503	15377
14626	15504	15378
14628	15512	15379
14632	15513	15382
14633	15519	15384
14634	15521	15385
14635	15523	15387
14640	15524	15391
14641	15528	15393
14642	15531	15395
14643	15533	15396
14644	15534	15397
14645	15537	15399
14646	15540	15400
14647	15541	15404
14649	15543	15405
14650	15544	15409
14651	15547	15410
14654	15548	15412
14657	15550	15413
14660	15551	15415
14661	15558	15416
14662	15563	15417
14663	15564	15420
14665	15565	15423
14666	15567	15424
14668	15569	15428
14669	15570	15430
14670	15573	15433

14673	15574	15436
14674	15575	15438
14676	15576	15440
14679	15578	15441
14681	15580	15442
14682	15581	15445
14683	15582	15446
14684	15584	15447
14686	15586	15452
14687	15589	15453
14688	15590	15457
14691	15592	15458
14694	15594	15459
14696	15595	15460
14697	15598	15465
14698	15602	15473
14700	15605	15487
14701	15606	15500
14702	15607	15512
14703	15608	15523
14705	15609	15533
14706	15610	15534
14707	15611	15537
14709	15612	15538
14710	15613	15542
14712	15615	15552
14714	15617	15553
14715	15618	15558
14718	15624	15560
14719	15625	15564
14722	15627	15575
14723	15628	15580
14724	15629	15582
14726	15630	15586
14729	15631	15589
14731	15633	15593
14732	15634	15594
14733	15635	15612
14734	15636	15613
14735	15638	15614
14737	15639	15615
14738	15640	15617
14739	15642	15618
14742	15643	15624
14743	15645	15627
14744	15646	15633

14746	15649	15641
14747	15650	15644
14751	15653	15646
14753	15654	15649
14755	15655	15653
14759	15658	15655
14765	15661	15657
14766	15662	15670
14767	15667	15673
14770	15668	15676
14772	15669	15677
14773	15670	15678
14774	15672	15682
14775	15676	15683
14776	15677	15686
14777	15678	15687
14778	15679	15690
14779	15683	15691
14780	15686	15694
14781	15688	15699
14784	15689	15703
14787	15690	15704
14788	15691	15710
14790	15692	15712
14792	15694	15713
14794	15695	15716
14795	15698	15717
14796	15699	15726
14800	15700	15729
14801	15701	15731
14802	15703	16012
14805	15704	16023
14807	15706	16030
14808	15708	16059
14810	15709	16105
14812	15711	16108
14813	15712	16112
14816	15714	16114
14818	15717	16117
14820	15719	16118
14822	15720	16119
14824	15721	16123
14825	15722	16125
14827	15723	16126
14829	15724	16127
14830	15725	16128

14833	15726	16130
14834	15727	16131
14836	15729	16132
14837	15730	16134
14839	15731	16135
14840	3	16136
14842	15	16137
14843	39	16138
14844	42	16139
14850	64	16140
14852	77	16141
14853	93	16145
14855	99	16147
14856	104	16148
14857	119	16149
14858	138	16153
14859	148	16155
14860	153	16156
14861	157	16157
14862	165	16159
14864	180	16162
14866	211	16164
14868	236	16165
14874	238	16166
14875	243	16167
14878	247	16191
14882	258	16195
14883	271	16196
14884	272	16199
14885	288	16201
14887	295	16202
14890	298	16205
14891	320	16206
14892	345	16209
14893	351	16210
14894	358	16212
14895	384	16213
14897	405	16214
14899	407	16215
14900	408	16216
14903	412	16217
14905	417	16218
14906	419	16219
14908	426	16221
14912	440	16222
14913	463	16223

14915	492	16224
14916	512	16225
14917	536	16226
14919	537	16227
14923	544	16228
14926	558	16230
14927	560	16231
14928	561	16232
14932	562	16233
14936	567	16235
14938	570	16236
14940	574	16238
14941	577	16239
14942	580	16241
14943	582	16242
14945	583	16244
14947	585	16246
14948	592	16248
14951	593	16250
14957	594	16251
14958	596	16253
14960	599	16255
14962	611	16257
14965	613	16258
14970	631	16259
14972	654	16260
14975	678	16261
14978	686	16262
14981	693	16263
14991	709	16264
15007	710	16265
15022	712	16266
15023	713	16268
15025	724	16269
15026	729	16270
15031	771	16271
15034	776	16272
15036	784	16274
15040	795	16275
15046	834	16277
15047	847	16278
15050	850	16279
15051	860	16280
15060	864	16281
15062	877	16283
15080	878	16284

15088	885	16285
15089	889	16286
15090	890	16287
15096	895	16288
15097	900	16289
15099	909	16290
15101	910	16291
15103	917	16292
15108	920	16294
15109	935	16295
15110	936	16298
15111	942	16349
15112	952	16372
15115	954	16380
15124	955	16388
15125	956	16391
15126	958	16403
15128	970	16406
15132	980	16418
15139	986	16428
15145	988	16435
15147	993	16440
15152	994	16446
15159	999	16449
15160	1007	16451
15162	1016	16455
15171	1030	16456
15174	1074	16457
15176	1108	16458
15181	1110	16468
15183	1118	16471
15184	1121	16472
15195	1122	16475
15198	1123	16476
15199	1127	16480
15203	1150	16483
15210	1159	16486
15212	1160	16488
15219	1176	16489
15222	1181	16514
15225	1211	16516
15226	1217	16517
15227	1241	16518
15228	1242	16526
15229	1319	16530
15230	1320	16532

15231	1321	16534
15232	1322	16540
15236	1336	16541
15238	1359	16542
15242	1365	16543
15243	1388	16544
15245	1406	16545
15247	1432	16547
15249	1435	16548
15250	1441	16549
15251	1459	16550
15252	1465	16553
15253	1467	16555
15254	1483	16557
15255	1488	16558
15260	1492	16559
15263	1502	16561
15271	1506	16562
15278	1535	16564
15294	1553	16565
15300	1566	16568
15307	1592	16569
15313	1624	16570
15316	1661	16572
15317	1663	16573
15330	1665	16574
15341	1672	16575
15343	1685	16576
15344	1693	16578
15346	1697	16579
15351	1701	16582
15355	1711	16583
15358	1728	16585
15359	1735	16586
15361	1739	16587
15370	1741	16588
15378	1746	1
15384	1763	14
15385	1771	15
15391	1784	20
15393	1788	42
15395	1825	59
15398	1829	60
15400	1841	62
15409	1858	64
15411	1862	77

15413	1864	103
15419	1867	112
15420	1875	113
15433	1894	119
15435	1910	121
15438	1955	141
15446	1962	143
15459	1982	148
15467	1990	159
15472	2004	165
15498	2010	226
15521	2056	272
15567	2077	278
15590	2109	296
15594	2115	324
15611	2116	345
15613	2120	358
15637	2129	361
15656	2134	387
15721	2137	390
15734	2154	410
15751	2166	416
15752	2168	426
15753	2173	427
15754	2176	446
15755	2179	455
15757	2186	456
15758	2192	462
15760	2194	465
15762	2201	472
15767	2202	478
15769	2282	492
15770	2300	526
15771	2311	536
15940	2334	542
16168	2337	544
16169	2341	546
16170	2346	553
16171	2352	555
16174	2353	556
16175	2360	558
16176	2379	561
16177	2381	562
16178	2393	565
16179	2404	566
16180	2421	570

16183	2435	574
16184	2440	580
16186	2453	585
16187	2455	590
16189	2460	591
16190	2474	594
16491	2484	598
16492	2485	607
16493	2488	610
16494	2500	611
16496	2506	612
16497	2517	613
16498	2528	631
16499	2529	678
16501	2531	680
16502	2533	682
16504	2548	686
16505	2552	693
16507	2564	694
16508	2570	697
16509	2571	700
16510	2580	701
16511	2581	712
16768	2582	713
16769	2584	728
16770	2585	733
16771	2587	734
16772	2588	743
12	2602	760
15	2614	781
19	2665	784
20	2694	800
39	2697	839
42	2708	847
45	2710	853
60	2731	874
64	2762	880
93	2766	887
99	2768	890
119	2770	895
139	2787	898
148	2802	900
149	2805	904
153	2810	909
155	2813	910
157	2817	914

159	2824	920
163	2839	935
165	2873	936
173	2878	938
174	2882	942
177	2889	954
209	2898	956
217	2900	958
239	2902	967
262	2906	970
265	2912	991
266	2920	993
272	2923	994
287	2939	995
288	2941	999
295	2951	1054
343	2953	1074
345	2955	1083
358	2960	1091
365	2961	1093
380	2990	1097
390	3008	1110
395	3023	1118
398	3026	1122
412	3030	1127
417	3033	1152
420	3045	1160
426	3060	1182
440	3073	1184
448	3089	1185
450	3105	1199
458	3113	1217
478	3118	1263
493	3120	1271
495	3121	1282
526	3152	1283
535	3179	1284
536	3186	1293
544	3188	1302
546	3194	1303
548	3196	1319
553	3212	1321
556	3214	1342
557	3225	1353
560	3226	1378
561	3228	1401

562	3230	1418
570	3237	1425
583	3243	1427
589	3244	1435
590	3248	1437
600	3250	1444
607	3265	1445
615	3275	1453
648	3284	1461
651	3303	1483
659	3304	1492
666	3311	1493
678	3326	1502
680	3333	1530
682	3336	1543
688	3340	1592
701	3342	1593
702	3366	1596
709	3372	1634
713	3379	1662
727	3382	1663
746	3384	1671
776	3429	1672
781	3435	1675
782	3437	1685
784	3442	1686
795	3488	1693
800	3493	1696
834	3495	1725
839	3517	1726
841	3518	1735
855	3532	1739
860	3535	1784
862	3536	1788
873	3569	1811
885	3590	1825
889	3592	1830
893	3594	1834
895	3608	1838
900	3609	1841
909	3612	1846
914	3624	1856
917	3625	1858
920	3627	1861
922	3628	1862
936	3629	1864

938	3649	1867
942	3672	1875
958	3700	1880
964	3710	1903
970	3715	1910
991	3732	1955
995	3740	1995
996	3771	2004
1000	3775	2010
1016	3788	2011
1032	3791	2016
1048	3818	2023
1062	3820	2056
1068	3830	2074
1074	3832	2077
1080	3835	2115
1083	3836	2120
1089	3842	2134
1101	3846	2137
1102	3851	2150
1118	3856	2159
1121	3870	2165
1125	3877	2166
1127	3881	2168
1133	3885	2172
1141	3886	2173
1142	3908	2190
1150	3911	2198
1152	3942	2202
1154	3965	2205
1160	3975	2241
1169	3976	2242
1176	3981	2258
1187	3992	2296
1192	3994	2300
1196	4017	2304
1199	4020	2308
1207	4023	2314
1208	4031	2334
1213	4068	2340
1217	4071	2345
1240	4079	2346
1251	4093	2381
1263	4098	2390
1265	4107	2392
1298	4112	2393

1317	4124	2394
1321	4130	2421
1329	4134	2435
1331	4136	2453
1359	4150	2455
1393	4152	2460
1405	4156	2475
1406	4161	2500
1427	4166	2509
1435	4170	2517
1436	4183	2525
1444	4191	2529
1448	4194	2552
1465	4196	2556
1474	4198	2580
1490	4203	2581
1492	4213	2582
1503	4215	2595
1506	4218	2627
1516	4219	2671
1593	4222	2672
1624	4229	2687
1625	4232	2689
1630	4262	2692
1635	4263	2715
1663	4277	2734
1665	4281	2748
1673	4293	2802
1675	4299	2805
1676	4312	2808
1684	4314	2810
1686	4318	2813
1696	4332	2817
1697	4340	2841
1702	4342	2872
1705	4343	2878
1711	4350	2882
1713	4378	2884
1735	4386	2899
1749	4396	2906
1754	4402	2920
1769	4411	2937
1776	4416	2941
1784	4417	2949
1785	4418	2951
1793	4419	2960

1797	4428	2961
1811	4433	2983
1815	4444	3001
1825	4447	3008
1829	4463	3023
1830	4476	3028
1845	4483	3032
1854	4485	3036
1858	4487	3042
1862	4516	3045
1864	4517	3057
1867	4519	3060
1873	4526	3063
1910	4534	3069
1915	4538	3076
1921	4546	3081
1941	4557	3139
1955	4561	3140
1980	4562	3142
2009	4567	3147
2010	4582	3152
2023	4584	3160
2045	4585	3177
2053	4597	3179
2082	4607	3187
2091	4625	3224
2116	4629	3225
2134	4635	3238
2165	4650	3240
2173	4652	3241
2176	4658	3244
2179	4662	3247
2185	4697	3250
2192	4704	3283
2202	4706	3285
2229	4711	3304
2244	4713	3308
2247	4737	3311
2260	4751	3335
2291	4753	3352
2297	4756	3356
2299	4760	3363
2312	4778	3366
2314	4785	3379
2334	4786	3382
2337	4800	3395

2346	4805	3410
2352	4813	3416
2353	4824	3419
2378	4854	3421
2381	4892	3427
2393	4894	3437
2401	4901	3442
2404	4906	3466
2413	4907	3469
2418	4920	3472
2421	4921	3493
2430	4959	3495
2432	4980	3497
2440	5006	3517
2459	5008	3518
2467	5029	3519
2484	5032	3521
2490	5074	3531
2500	5079	3544
2513	5101	3548
2515	5104	3590
2517	5114	3594
2521	5137	3608
2523	5142	3609
2528	5147	3612
2529	5201	3624
2565	5223	3625
2584	5246	3628
2585	5252	3629
2590	5263	3649
2610	5291	3661
2616	5322	3663
2624	5324	3669
2625	5325	3672
2672	5334	3679
2682	5339	3684
2686	5347	3695
2690	5360	3698
2693	5368	3706
2694	5369	3710
2695	5372	3713
2710	5386	3729
2734	5397	3732
2750	5409	3754
2775	5427	3773
2802	5451	3775

2805	5468	3788
2808	5470	3819
2810	5472	3835
2827	5482	3846
2851	5485	3848
2864	5503	3870
2898	5505	3875
2918	5508	3885
2931	5515	3886
2936	5517	3905
2939	5540	3936
2948	5545	3956
2951	5584	3974
2955	5588	3983
3011	5602	3992
3020	5605	3998
3026	5606	4017
3032	5608	4020
3045	5610	4023
3056	5618	4035
3058	5622	4038
3069	5624	4044
3075	5626	4047
3076	5636	4070
3090	5645	4073
3093	5652	4076
3117	5685	4079
3124	5726	4091
3131	5735	4093
3139	5737	4100
3140	5740	4104
3141	5746	4110
3152	5761	4112
3160	5807	4113
3161	5817	4118
3176	5843	4122
3211	5851	4123
3220	5855	4124
3230	5869	4130
3238	5882	4136
3244	5886	4156
3247	5901	4179
3248	5903	4198
3250	5913	4207
3262	5930	4213
3270	5932	4218

3276	5941	4219
3284	5959	4229
3311	5977	4239
3333	5983	4249
3335	5984	4305
3336	5990	4312
3363	6005	4318
3379	6009	4333
3382	6010	4350
3417	6016	4353
3419	6021	4355
3448	6042	4365
3467	6046	4370
3479	6047	4378
3493	6051	4379
3495	6071	4396
3517	6077	4402
3544	6078	4416
3552	6088	4428
3553	6089	4429
3569	6091	4458
3574	6121	4464
3590	6123	4467
3609	6136	4468
3624	6137	4471
3629	6141	4473
3651	6172	4477
3663	6186	4481
3666	6204	4483
3669	6209	4487
3672	6211	4496
3686	6233	4497
3698	6260	4516
3706	6262	4517
3710	6284	4519
3713	6286	4521
3714	6287	4523
3720	6304	4524
3730	6320	4526
3741	6324	4534
3751	6330	4557
3763	6333	4567
3769	6336	4568
3791	6340	4582
3795	6359	4584
3803	6388	4590

3818	6389	4597
3820	6400	4599
3830	6407	4606
3832	6415	4607
3846	6421	4609
3850	6429	4617
3856	6432	4629
3870	6439	4650
3910	6443	4651
3914	6448	4652
3929	6453	4662
3936	6471	4669
3951	6476	4692
3954	6482	4751
3957	6535	4754
3974	6538	4756
3975	6558	4757
3976	6599	4784
3994	6601	4802
3998	6613	4805
4003	6625	4817
4005	6629	4824
4009	6642	4836
4010	6649	4841
4027	6658	4864
4041	6662	4870
4042	6669	4874
4053	6673	4892
4076	6697	4898
4079	6706	4921
4107	6714	4927
4112	6728	4934
4115	6730	4947
4117	6731	4948
4118	6737	4959
4125	6753	4965
4130	6756	5004
4136	6766	5005
4143	6767	5008
4150	6769	5029
4156	6773	5069
4163	6776	5070
4170	6778	5080
4183	6779	5083
4194	6780	5090
4197	6786	5100

4198	6793	5101
4213	6796	5102
4215	6803	5105
4218	6823	5112
4219	6826	5114
4222	6829	5121
4229	6836	5122
4245	6858	5130
4261	6874	5134
4273	6876	5137
4288	6880	5151
4303	6890	5205
4305	6891	5223
4312	6901	5245
4332	6918	5257
4340	6919	5261
4345	6923	5263
4355	6926	5284
4360	6938	5287
4366	6942	5288
4378	6951	5300
4408	6954	5301
4409	6968	5324
4417	6979	5325
4418	6982	5331
4426	6988	5337
4447	7013	5338
4448	7027	5339
4456	7033	5343
4462	7036	5344
4464	7043	5346
4468	7065	5353
4473	7068	5356
4483	7069	5358
4496	7081	5360
4501	7089	5369
4514	7095	5381
4516	7111	5384
4517	7124	5397
4524	7144	5421
4529	7147	5427
4534	7148	5434
4536	7150	5436
4538	7163	5438
4584	7164	5452
4596	7171	5468

4597	7174	5482
4598	7180	5485
4607	7182	5503
4609	7191	5505
4617	7209	5506
4625	7210	5515
4629	7214	5527
4635	7228	5529
4638	7260	5530
4651	7262	5540
4658	7264	5545
4662	7268	5546
4671	7272	5547
4675	7277	5578
4689	7283	5584
4691	7284	5588
4692	7291	5590
4711	7297	5591
4716	7306	5606
4746	7307	5610
4756	7315	5611
4758	7326	5613
4765	7328	5622
4766	7332	5624
4778	7342	5626
4786	7351	5640
4800	7372	5645
4819	7373	5652
4821	7383	5654
4823	7388	5660
4824	7389	5662
4841	7412	5664
4851	7418	5675
4874	7421	5677
4906	7422	5705
4919	7423	5714
4921	7438	5726
4929	7441	5728
4934	7497	5737
4935	7500	5747
4940	7515	5748
4959	7553	5797
4965	7561	5807
4967	7563	5821
4974	7564	5822
5006	7567	5882

5007	7571	5887
5011	7572	5923
5032	7582	5938
5051	7583	5941
5071	7584	5942
5090	7585	5945
5102	7601	5954
5104	7602	5963
5112	7603	5975
5127	7605	5976
5170	7617	5983
5172	7620	5990
5201	7628	6005
5217	7639	6013
5218	7657	6016
5223	7701	6027
5231	7709	6045
5245	7726	6063
5253	7728	6065
5259	7733	6068
5263	7734	6077
5287	7735	6081
5298	7736	6082
5322	7738	6121
5324	7754	6122
5325	7761	6141
5327	7785	6142
5334	7786	6186
5344	7791	6187
5347	7810	6190
5352	7812	6200
5354	7813	6201
5360	7822	6222
5365	7828	6249
5368	7836	6259
5369	7870	6260
5372	7877	6262
5373	7889	6264
5376	7899	6283
5386	7906	6293
5421	7924	6294
5432	7925	6317
5437	7937	6326
5456	7944	6327
5472	7945	6333
5485	7946	6340

5495	7955	6359
5500	7958	6383
5505	7960	6396
5508	7964	6400
5515	7966	6401
5529	7973	6421
5530	7975	6423
5547	7977	6432
5569	7984	6436
5583	7990	6439
5584	7991	6448
5588	8028	6458
5605	8029	6471
5610	8033	6476
5611	8047	6493
5620	8060	6506
5624	8063	6525
5627	8064	6528
5633	8065	6534
5636	8070	6535
5639	8072	6545
5652	8079	6556
5654	8080	6589
5657	8083	6593
5660	8085	6610
5686	8097	6623
5690	8103	6624
5701	8126	6628
5726	8158	6629
5740	8167	6633
5747	8171	6639
5748	8173	6658
5752	8176	6666
5761	8177	6675
5765	8181	6679
5783	8184	6692
5797	8187	6696
5815	8189	6702
5817	8204	6707
5819	8211	6713
5843	8222	6721
5851	8272	6722
5863	8274	6726
5901	8285	6728
5909	8291	6730
5923	8304	6746

5924	8309	6766
5931	8316	6767
5941	8329	6768
5952	8341	6773
5959	8354	6774
5963	8370	6777
5966	8375	6778
5971	8378	6786
5975	8397	6796
5976	8404	6803
5983	8412	6804
5990	8414	6805
5991	8430	6813
6005	8447	6816
6007	8452	6823
6009	8456	6858
6013	8457	6862
6016	8470	6863
6021	8482	6867
6024	8483	6874
6027	8485	6880
6032	8486	6883
6054	8489	6885
6060	8501	6890
6064	8507	6892
6067	8508	6904
6071	8513	6917
6081	8515	6918
6089	8535	6923
6091	8543	6926
6093	8544	6943
6097	8556	6954
6100	8559	6959
6108	8560	6988
6121	8566	7006
6123	8570	7008
6139	8574	7010
6141	8585	7014
6144	8587	7017
6172	8592	7027
6190	8607	7033
6221	8608	7036
6249	8615	7043
6269	8621	7068
6283	8622	7081
6284	8623	7083

6285	8628	7084
6286	8630	7093
6287	8634	7119
6293	8670	7125
6306	8672	7142
6307	8674	7144
6317	8677	7148
6327	8684	7150
6330	8703	7163
6332	8717	7169
6340	8719	7198
6354	8722	7214
6359	8728	7239
6363	8730	7260
6368	8741	7268
6377	8744	7272
6386	8770	7273
6387	8771	7282
6392	8781	7283
6400	8783	7291
6401	8785	7303
6406	8794	7306
6423	8806	7307
6432	8825	7318
6476	8831	7325
6495	8866	7326
6523	8870	7328
6525	8871	7332
6531	8878	7334
6535	8881	7336
6558	8896	7345
6583	8902	7360
6585	8914	7366
6591	8918	7367
6594	8923	7371
6610	8928	7372
6616	8933	7379
6623	8940	7383
6625	8948	7385
6666	8949	7388
6673	8965	7389
6679	8978	7404
6702	8980	7425
6707	8982	7433
6716	8984	7448
6727	8991	7467

6728	9012	7471
6732	9015	7474
6737	9017	7479
6757	9024	7482
6767	9030	7485
6773	9032	7502
6777	9033	7511
6778	9040	7515
6782	9041	7532
6791	9044	7572
6793	9049	7573
6796	9054	7575
6809	9063	7576
6811	9069	7582
6823	9078	7583
6829	9080	7584
6839	9084	7585
6862	9085	7589
6873	9093	7594
6874	9098	7600
6890	9106	7602
6891	9110	7605
6926	9116	7609
6938	9127	7638
6951	9132	7641
6967	9135	7649
6968	9137	7657
6971	9150	7665
6972	9160	7675
6979	9163	7690
6989	9170	7701
7001	9171	7709
7004	9174	7726
7010	9180	7732
7017	9185	7750
7027	9186	7751
7028	9199	7774
7033	9203	7782
7052	9204	7784
7068	9205	7786
7071	9207	7791
7080	9216	7802
7081	9220	7804
7083	9223	7807
7101	9235	7812
7123	9263	7822

7133	9269	7855
7144	9273	7857
7148	9274	7871
7150	9277	7880
7174	9279	7890
7182	9295	7894
7210	9301	7898
7211	9311	7903
7259	9320	7916
7262	9325	7918
7268	9330	7937
7272	9339	7944
7277	9351	7945
7283	9358	7955
7311	9362	7966
7332	9366	7968
7336	9370	7973
7344	9371	7977
7351	9372	7982
7372	9377	7983
7374	9388	7985
7383	9391	7990
7387	9398	7995
7421	9399	8003
7423	9408	8007
7425	9415	8009
7440	9429	8011
7457	9431	8028
7467	9458	8044
7474	9460	8045
7482	9469	8055
7494	9472	8060
7497	9475	8062
7509	9480	8064
7513	9483	8077
7515	9485	8079
7531	9501	8083
7544	9516	8085
7561	9523	8097
7572	9529	8105
7574	9545	8145
7575	9551	8155
7582	9556	8167
7584	9559	8169
7585	9560	8173
7586	9567	8177

7603	9574	8189
7605	9576	8204
7606	9587	8211
7608	9596	8221
7617	9597	8260
7620	9607	8274
7621	9617	8285
7649	9627	8291
7690	9630	8299
7717	9637	8302
7748	9649	8304
7761	9654	8309
7767	9657	8314
7782	9666	8316
7786	9670	8321
7802	9674	8323
7849	9678	8337
7855	9680	8358
7889	9692	8361
7906	9694	8364
7918	9696	8378
7928	9699	8383
7934	9711	8385
7937	9712	8392
7946	9715	8404
7955	9733	8427
7966	9734	8430
7974	9736	8447
7975	9742	8456
7982	9752	8470
7984	9761	8486
7986	9764	8497
7990	9767	8508
7995	9769	8513
8003	9775	8519
8029	9780	8530
8047	9782	8539
8055	9794	8541
8058	9806	8543
8061	9808	8545
8063	9813	8549
8066	9816	8551
8079	9830	8555
8080	9831	8559
8083	9835	8560
8095	9839	8566

8100	9842	8569
8103	9851	8570
8105	9855	8591
8140	9857	8593
8145	9863	8599
8158	9869	8600
8171	9874	8606
8177	9884	8607
8181	9887	8615
8184	9891	8620
8202	9903	8621
8204	9906	8622
8212	9919	8625
8222	9922	8626
8231	9941	8628
8249	9945	8638
8291	9949	8657
8310	9960	8677
8316	9966	8690
8329	9969	8694
8378	9978	8699
8392	9979	8703
8404	9993	8728
8412	9998	8730
8414	9999	8770
8420	10008	8775
8426	10011	8781
8434	10012	8785
8439	10023	8800
8447	10028	8807
8456	10030	8818
8470	10033	8829
8483	10061	8849
8497	10066	8851
8500	10073	8873
8506	10081	8887
8513	10097	8892
8519	10106	8894
8537	10120	8902
8539	10141	8916
8543	10142	8918
8556	10145	8923
8559	10161	8925
8566	10177	8926
8570	10178	8949
8574	10193	8964

8585	10196	8975
8592	10207	8978
8594	10208	8980
8614	10211	8984
8615	10229	8993
8624	10234	8996
8625	10238	9015
8628	10246	9017
8655	10255	9022
8677	10260	9030
8678	10273	9039
8679	10275	9051
8682	10278	9052
8699	10281	9057
8717	10291	9058
8722	10302	9082
8730	10303	9086
8734	10304	9090
8741	10312	9093
8744	10317	9098
8757	10322	9099
8771	10325	9109
8781	10326	9116
8785	10334	9127
8792	10337	9132
8800	10341	9135
8801	10342	9142
8823	10349	9160
8829	10360	9161
8847	10376	9162
8848	10377	9169
8849	10383	9170
8854	10392	9174
8871	10397	9178
8878	10413	9180
8882	10430	9189
8894	10435	9190
8896	10437	9200
8902	10450	9203
8916	10457	9204
8920	10462	9205
8922	10465	9207
8923	10467	9215
8925	10477	9216
8930	10478	9219
8932	10483	9227

8933	10493	9229
8934	10496	9242
8935	10497	9244
8943	10504	9252
8963	10508	9263
8965	10518	9264
8974	10519	9267
8984	10521	9269
8996	10534	9274
9007	10538	9279
9017	10545	9283
9030	10547	9295
9033	10559	9302
9035	10577	9322
9040	10578	9325
9041	10600	9330
9044	10624	9352
9049	10625	9353
9054	10637	9358
9057	10643	9370
9078	10660	9371
9081	10668	9372
9084	10669	9388
9085	10676	9391
9086	10680	9397
9090	10686	9399
9093	10692	9414
9098	10696	9426
9099	10698	9429
9105	10699	9440
9109	10716	9450
9111	10722	9452
9116	10725	9454
9149	10727	9457
9160	10736	9461
9161	10739	9462
9163	10746	9472
9171	10753	9480
9174	10754	9483
9199	10764	9487
9203	10765	9490
9204	10772	9491
9205	10776	9494
9207	10781	9496
9215	10793	9498
9220	10794	9515

9229	10797	9516
9245	10800	9528
9251	10802	9529
9274	10815	9545
9279	10821	9556
9295	10837	9559
9301	10838	9560
9302	10848	9564
9304	10852	9567
9305	10856	9571
9307	10862	9580
9311	10872	9581
9314	10881	9587
9320	10888	9591
9322	10890	9592
9324	10896	9595
9325	10899	9617
9330	10909	9625
9337	10924	9630
9339	10928	9637
9351	10929	9638
9358	10930	9639
9366	10939	9657
9370	10960	9663
9371	10961	9666
9388	10964	9670
9391	10970	9677
9399	10982	9680
9404	10998	9690
9405	11019	9694
9408	11023	9699
9414	11024	9702
9415	11029	9703
9426	11038	9715
9430	11061	9723
9431	11063	9725
9440	11067	9728
9442	11072	9733
9443	11099	9734
9450	11110	9740
9454	11121	9742
9457	11123	9755
9467	11129	9767
9472	11137	9775
9486	11140	9780
9487	11151	9782

9488	11153	9794
9491	11157	9795
9496	11161	9806
9503	11165	9810
9506	11174	9813
9511	11188	9816
9515	11189	9821
9545	11202	9830
9547	11204	9833
9556	11211	9842
9559	11220	9857
9560	11222	9869
9574	11236	9874
9587	11250	9884
9592	11266	9903
9595	11267	9905
9596	11276	9907
9606	11279	9909
9607	11283	9926
9617	11302	9930
9625	11304	9931
9628	11307	9938
9630	11319	9942
9649	11356	9945
9654	11361	9948
9657	11366	9953
9674	11369	9958
9692	11370	9968
9694	11375	9979
9696	11378	9988
9699	11390	9998
9702	11397	10002
9703	11400	10007
9704	11402	10008
9712	11408	10012
9715	11411	10016
9717	11418	10025
9718	11424	10033
9728	11429	10061
9736	11438	10068
9740	11443	10072
9742	11445	10074
9744	11458	10081
9752	11472	10106
9760	11481	10117
9761	11489	10135

9764	11515	10141
9768	11540	10143
9769	11549	10145
9776	11562	10148
9777	11571	10159
9780	11582	10163
9782	11605	10165
9794	11619	10170
9808	11620	10177
9814	11621	10181
9830	11624	10186
9831	11633	10199
9841	11643	10207
9842	11649	10208
9845	11654	10211
9846	11657	10213
9849	11674	10234
9855	11688	10242
9856	11699	10249
9869	11704	10260
9881	11709	10261
9887	11712	10264
9891	11714	10269
9914	11734	10270
9915	11737	10281
9922	11741	10302
9923	11747	10317
9937	11748	10322
9941	11752	10325
9945	11753	10334
9949	11755	10342
9951	11756	10347
9952	11757	10349
9953	11758	10353
9963	11769	10375
9965	11774	10376
9969	11775	10384
9979	11778	10387
9983	11794	10392
9997	11797	10397
9998	11800	10405
9999	11815	10413
10008	11833	10430
10011	11839	10441
10014	11841	10442
10020	11853	10444

10023	11856	10458
10025	11879	10477
10033	11889	10478
10051	11894	10479
10061	11895	10483
10065	11898	10486
10066	11915	10487
10081	11917	10493
10082	11944	10504
10091	11951	10519
10097	11952	10521
10109	11965	10529
10111	11969	10538
10141	11975	10545
10142	11985	10547
10145	11987	10549
10152	12006	10553
10159	12021	10562
10161	12036	10568
10162	12037	10577
10175	12048	10580
10177	12051	10586
10186	12052	10596
10193	12059	10608
10196	12068	10624
10199	12072	10636
10207	12077	10639
10208	12082	10649
10211	12089	10655
10229	12099	10665
10237	12100	10672
10238	12102	10676
10239	12105	10677
10247	12106	10680
10253	12107	10681
10255	12117	10692
10256	12118	10695
10264	12146	10696
10267	12147	10698
10272	12151	10714
10281	12163	10716
10302	12167	10722
10303	12169	10724
10312	12194	10725
10317	12200	10727
10319	12201	10731

10322	12210	10738
10325	12213	10739
10331	12219	10746
10334	12224	10753
10337	12231	10754
10342	12238	10756
10353	12240	10757
10359	12260	10791
10375	12265	10793
10376	12278	10797
10377	12287	10802
10392	12294	10810
10405	12306	10811
10413	12316	10815
10426	12318	10830
10441	12320	10834
10465	12329	10849
10467	12336	10876
10477	12352	10883
10478	12374	10888
10493	12376	10890
10497	12379	10899
10504	12387	10905
10518	12391	10917
10534	12405	10918
10542	12421	10928
10545	12427	10942
10549	12435	10952
10560	12441	10954
10577	12445	10956
10592	12453	10960
10596	12460	10961
10608	12468	10963
10625	12480	10964
10637	12485	10972
10660	12491	10991
10672	12493	11006
10676	12504	11007
10677	12510	11015
10680	12527	11023
10692	12528	11039
10695	12536	11053
10696	12540	11061
10699	12545	11072
10709	12559	11081
10721	12570	11085

10722	12578	11086
10727	12586	11087
10739	12589	11115
10741	12590	11121
10746	12594	11123
10753	12600	11129
10756	12601	11130
10764	12618	11137
10765	12620	11140
10772	12624	11141
10793	12627	11144
10794	12632	11151
10819	12637	11153
10830	12656	11154
10837	12661	11184
10838	12663	11188
10851	12667	11203
10855	12683	11204
10869	12684	11205
10890	12695	11211
10893	12696	11220
10898	12701	11231
10899	12702	11246
10924	12707	11266
10928	12708	11267
10942	12717	11284
10947	12726	11289
10954	12728	11297
10960	12730	11302
10961	12755	11305
10970	12769	11306
10982	12775	11310
10984	12783	11319
10997	12807	11331
10998	12811	11334
11023	12823	11342
11029	12827	11348
11039	12846	11349
11061	12873	11365
11063	12874	11375
11088	12876	11378
11104	12880	11390
11105	12885	11391
11120	12892	11397
11123	12913	11403
11129	12916	11404

11151	12929	11411
11178	12930	11414
11184	12934	11416
11187	12939	11419
11188	12952	11424
11189	12972	11427
11220	12975	11429
11224	12980	11436
11240	13001	11443
11241	13003	11447
11250	13005	11459
11267	13006	11466
11276	13008	11472
11279	13016	11481
11283	13020	11488
11284	13024	11489
11299	13026	11493
11302	13028	11496
11304	13029	11503
11307	13037	11515
11331	13041	11518
11334	13063	11524
11335	13071	11525
11349	13077	11526
11354	13078	11537
11356	13107	11549
11378	13114	11566
11390	13126	11571
11400	13129	11577
11422	13163	11592
11438	13164	11605
11443	13191	11607
11444	13214	11619
11459	13225	11620
11463	13229	11633
11472	13241	11643
11481	13253	11649
11487	13256	11657
11524	13267	11674
11537	13275	11677
11549	13277	11687
11562	13286	11688
11571	13290	11712
11577	13297	11715
11584	13301	11730
11590	13328	11731

11598	13330	11734
11607	13338	11737
11613	13344	11744
11620	13347	11749
11633	13358	11755
11638	13367	11756
11657	13377	11765
11674	13384	11775
11687	13387	11778
11688	13388	11784
11712	13391	11794
11715	13415	11797
11720	13422	11800
11722	13435	11803
11734	13445	11810
11737	13467	11838
11738	13474	11848
11744	13482	11852
11747	13483	11853
11753	13492	11854
11758	13497	11864
11775	13526	11865
11794	13559	11879
11797	13564	11883
11838	13567	11892
11839	13582	11899
11853	13605	11904
11854	13620	11930
11862	13629	11936
11863	13630	11945
11881	13637	11952
11886	13644	11979
11891	13645	11980
11915	13654	11983
11927	13655	12000
11939	13674	12021
11944	13679	12037
11945	13691	12048
11952	13704	12052
11975	13705	12059
11976	13716	12060
11979	13724	12087
11999	13725	12093
12000	13756	12094
12006	13757	12099
12021	13768	12100

12026	13769	12106
12048	13810	12107
12053	13814	12112
12059	13829	12115
12060	13835	12155
12072	13837	12156
12083	13855	12163
12089	13885	12170
12098	13886	12179
12099	13887	12201
12102	13891	12207
12105	13906	12214
12107	13913	12218
12146	13922	12226
12163	13934	12228
12195	13938	12232
12200	13960	12235
12219	14005	12238
12226	14012	12244
12238	14019	12255
12241	14023	12278
12255	14034	12294
12256	14035	12300
12265	14047	12310
12268	14053	12318
12278	14059	12320
12300	14075	12328
12308	14087	12336
12310	14108	12351
12316	14112	12370
12318	14127	12376
12329	14177	12387
12336	14178	12391
12370	14190	12405
12376	14203	12407
12391	14214	12421
12402	14221	12424
12405	14232	12444
12407	14248	12453
12441	14254	12460
12447	14260	12468
12450	14272	12474
12453	14274	12475
12460	14297	12485
12480	14305	12492
12481	14316	12493

12493	14338	12498
12502	14343	12508
12504	14348	12527
12523	14370	12528
12528	14384	12568
12529	14391	12582
12558	14401	12589
12586	14404	12594
12589	14414	12601
12600	14432	12624
12601	14441	12632
12604	14460	12634
12649	14470	12647
12667	14478	12656
12681	14488	12661
12683	14493	12666
12688	14494	12667
12702	14502	12681
12717	14506	12683
12726	14519	12695
12727	14525	12702
12728	14554	12707
12755	14566	12717
12784	14569	12726
12806	14592	12727
12807	14609	12784
12823	14623	12789
12852	14625	12792
12861	14680	12807
12878	14705	12813
12880	14706	12827
12882	14719	12834
12886	14721	12868
12892	14750	12874
12893	14793	12880
12906	14794	12886
12913	14807	12892
12929	14810	12893
12930	14826	12897
12937	14829	12913
12939	14842	12916
12972	14866	12923
12991	14873	12930
13005	14885	12939
13008	14890	12969
13014	14912	12972

13024	14917	12975
13026	14919	12993
13028	14972	13001
13029	14984	13005
13037	15030	13009
13041	15031	13016
13069	15035	13020
13071	15047	13024
13077	15058	13026
13094	15062	13029
13100	15069	13041
13111	15076	13061
13114	15083	13069
13131	15085	13077
13151	15088	13114
13164	15112	13146
13177	15115	13147
13196	15164	13164
13206	15182	13166
13212	15195	13181
13214	15198	13216
13223	15274	13223
13229	15291	13249
13236	15318	13256
13241	15324	13269
13245	15397	13278
13253	15498	13279
13267	15509	13290
13275	15517	13297
13286	15527	13304
13290	15530	13337
13292	15536	13355
13298	15545	13377
13328	15554	13384
13337	15560	13388
13350	15566	13393
13367	15571	13466
13377	15583	13468
13384	15588	13474
13388	15596	13477
13391	15599	13482
13422	15600	13559
13445	15601	13561
13461	15603	13562
13474	15604	13563
13492	15616	13564

13564	15619	13567
13567	15620	13568
13580	15622	13573
13582	15623	13576
13597	15637	13580
13610	15647	13610
13629	15663	13630
13637	15664	13645
13654	15665	13663
13678	15666	13667
13691	15671	13690
13701	15680	13724
13719	15693	13725
13724	15696	13753
13725	15697	13768
13727	15705	13771
13793	15707	13793
13814	15710	13798
13904	20	13804
13938	31	13829
13941	59	13853
13956	72	13868
13994	74	13887
14008	76	13913
14018	113	13945
14023	118	14008
14028	125	14019
14036	143	14030
14053	159	14036
14067	219	14039
14111	324	14042
14112	347	14059
14118	387	14067
14164	390	14086
14178	410	14108
14187	411	14117
14203	416	14192
14213	422	14213
14214	458	14214
14221	462	14219
14229	478	14263
14232	510	14271
14255	526	14295
14263	542	14316
14266	546	14390
14267	548	14391

14283	551	14392
14295	553	14399
14297	554	14432
14311	556	14477
14316	589	14519
14325	591	14531
14390	598	14533
14392	600	14547
14432	606	14565
14459	607	14609
14470	615	14643
14477	651	14673
14515	652	14680
14571	659	14706
14609	666	14718
14623	679	14721
14677	680	14750
14680	682	14759
14690	694	14763
14721	701	14808
14740	703	14813
14760	708	14834
14786	733	14885
14865	760	14890
14925	765	14970
14961	781	14998
15020	792	15017
15164	819	15063
15202	839	15064
15239	841	15115
15241	858	15123
15256	862	15146
15257	874	15181
15262	880	15198
15310	887	15201
15750	904	15204
15756	905	15212
15766	914	15240
15768	922	15255
16173	961	15299
16181	991	15317
16185	995	15318
16495	996	15326
16503	1018	15367
16506	1019	15386
6	1032	15389

31	1033	15398
59	1081	15451
67	1091	15531
76	1105	15539
77	1124	15548
101	1142	15592
112	1152	15605
151	1154	16113
180	1170	16129
188	1182	16194
210	1184	16207
211	1192	16229
212	1199	16376
228	1213	16379
275	1222	16381
279	1240	16382
306	1243	16383
322	1258	16385
324	1263	16387
327	1305	16390
387	1400	16401
401	1401	16402
410	1425	16404
416	1427	16405
442	1428	16411
472	1436	16414
473	1439	16417
492	1445	16422
515	1453	16425
530	1471	16427
537	1474	16429
542	1490	16430
545	1530	16432
551	1543	16433
554	1544	16434
585	1550	16437
591	1568	16438
592	1586	16441
598	1596	16444
599	1600	16448
606	1615	16453
610	1662	16454
613	1670	16459
631	1673	16461
654	1684	16463
660	1686	16465

662	1705	16466
679	1721	16470
691	1723	16473
694	1729	16477
703	1733	16481
708	1747	16482
736	1778	16485
752	1815	16487
760	1835	16490
765	1838	16512
780	1861	16513
791	1870	16515
808	1873	16520
821	1879	16523
838	1903	16524
849	1909	16525
887	1991	16527
899	1993	16529
905	1995	16531
931	2011	16533
932	2016	16535
961	2023	16536
967	2062	16537
981	2074	16538
986	2099	16539
1018	2122	16551
1051	2150	16552
1067	2165	16560
1072	2182	16563
1077	2185	16566
1081	2205	16577
1087	2231	16580
1091	2244	16581
1093	2247	16584
1095	2259	11
1097	2314	38
1119	2317	41
1168	2345	68
1170	2390	74
1174	2394	76
1181	2396	102
1214	2402	104
1222	2515	110
1249	2523	117
1303	2550	118
1305	2595	125

1342	2625	180
1395	2635	187
1400	2683	204
1425	2689	207
1437	2711	210
1453	2751	236
1471	2790	294
1487	2806	318
1491	2809	331
1493	2812	347
1502	2820	349
1530	2841	365
1550	2872	379
1568	2916	395
1574	2918	411
1582	2935	428
1584	2936	442
1592	2944	448
1596	2945	449
1600	3001	459
1614	3028	473
1662	3036	510
1664	3056	530
1683	3057	545
1685	3063	548
1690	3068	551
1700	3070	572
1712	3076	592
1726	3081	599
1729	3111	600
1731	3131	603
1733	3139	606
1739	3147	615
1744	3158	648
1767	3183	652
1812	3187	660
1838	3191	662
1856	3220	663
1875	3224	666
1879	3238	691
1894	3270	708
1903	3276	710
1909	3285	719
1968	3301	721
1993	3307	742
1995	3321	765

2044	3335	780
2074	3363	789
2083	3380	795
2122	3392	834
2124	3411	850
2137	3416	855
2139	3421	858
2150	3456	863
2154	3466	905
2155	3469	921
2159	3516	922
2168	3555	932
2172	3561	953
2182	3572	962
2186	3615	976
2190	3619	981
2205	3635	996
2238	3650	1018
2283	3651	1038
2296	3655	1051
2304	3661	1066
2311	3663	1081
2360	3669	1102
2366	3684	1111
2379	3698	1114
2390	3706	1115
2394	3722	1119
2402	3738	1121
2453	3742	1124
2455	3747	1141
2463	3751	1151
2474	3754	1154
2537	3763	1168
2544	3773	1170
2550	3785	1181
2572	3795	1222
2580	3819	1240
2581	3841	1251
2591	3860	1258
2621	3875	1304
2627	3910	1320
2633	3936	1336
2684	3974	1349
2687	3989	1400
2711	3998	1434
2723	4009	1454

2729	4010	1471
2806	4038	1490
2809	4041	1491
2846	4042	1499
2878	4047	1501
2935	4053	1516
2944	4091	1518
2945	4096	1584
2950	4110	1600
2956	4113	1614
2960	4122	1615
2961	4174	1659
3023	4175	1664
3030	4220	1683
3036	4230	1684
3057	4239	1688
3068	4249	1708
3070	4250	1712
3081	4255	1721
3091	4273	1723
3159	4290	1731
3182	4333	1733
3187	4353	1778
3191	4355	1790
3224	4370	1812
3240	4379	1813
3242	4409	1815
3253	4422	1821
3255	4426	1873
3292	4427	1879
3294	4458	1882
3303	4464	1909
3307	4467	1959
3339	4468	1993
3392	4473	2009
3411	4474	2044
3421	4477	2124
3437	4481	2145
3469	4496	2154
3482	4501	2160
3516	4521	2186
3559	4523	2193
3572	4524	2194
3628	4528	2229
3657	4548	2231
3661	4560	2247

3670	4590	2299
3679	4606	2311
3684	4609	2317
3736	4617	2331
3738	4620	2360
3773	4651	2378
3788	4654	2402
3819	4667	2405
3860	4718	2410
3875	4754	2422
3907	4757	2423
3908	4784	2432
3956	4806	2463
3980	4836	2491
3992	4841	2495
4038	4853	2515
4044	4880	2523
4047	4912	2550
4064	4929	2572
4092	4930	2591
4122	4934	2625
4131	4936	2655
4151	4986	2684
4161	5004	2694
4175	5007	2710
4207	5050	2711
4249	5063	2743
4250	5069	2806
4253	5085	2809
4293	5090	2812
4331	5095	2827
4379	5102	2846
4386	5103	2898
4396	5112	2924
4427	5121	2925
4428	5122	2935
4455	5130	2939
4480	5135	2942
4481	5138	2944
4521	5180	2945
4525	5240	2956
4528	5259	2972
4532	5261	3033
4548	5295	3058
4590	5296	3070
4606	5297	3105

4610	5320	3112
4650	5344	3118
4654	5346	3131
4667	5352	3137
4676	5354	3144
4717	5356	3164
4742	5358	3182
4753	5365	3183
4774	5373	3185
4805	5379	3220
4806	5381	3253
4817	5413	3277
4820	5418	3303
4836	5421	3307
4853	5436	3331
4892	5438	3361
4893	5449	3392
4912	5450	3411
5004	5452	3415
5005	5498	3417
5029	5511	3434
5050	5529	3456
5058	5530	3516
5063	5546	3558
5069	5547	3559
5070	5548	3561
5083	5569	3572
5095	5590	3635
5103	5613	3651
5105	5639	3655
5107	5640	3666
5122	5642	3670
5135	5657	3685
5200	5660	3699
5243	5662	3736
5250	5664	3738
5320	5705	3741
5326	5716	3749
5328	5727	3777
5329	5748	3779
5331	5780	3785
5346	5808	3799
5351	5822	3802
5356	5844	3803
5358	5887	3824
5378	5929	3825

5379	5933	3833
5384	5940	3841
5410	5942	3910
5413	5954	3969
5418	5971	3980
5425	5991	4010
5434	6013	4064
5436	6025	4092
5438	6027	4101
5490	6050	4175
5496	6068	4183
5511	6070	4196
5517	6081	4250
5524	6133	4273
5539	6142	4297
5578	6151	4358
5603	6200	4386
5613	6201	4408
5640	6222	4409
5646	6249	4410
5648	6259	4422
5662	6264	4431
5683	6283	4479
5716	6293	4485
5719	6303	4513
5766	6327	4532
5780	6346	4536
5844	6363	4548
5887	6383	4560
5905	6401	4596
5977	6409	4610
6050	6413	4612
6078	6451	4631
6114	6523	4647
6122	6525	4667
6133	6534	4714
6142	6556	4764
6151	6563	4765
6180	6589	4787
6200	6593	4820
6204	6610	4823
6300	6616	4831
6320	6623	4861
6362	6633	4862
6379	6666	4912
6413	6672	4929

6424	6702	4930
6439	6705	4977
6451	6707	5007
6471	6726	5028
6473	6741	5032
6475	6742	5033
6506	6746	5049
6534	6757	5058
6545	6784	5063
6552	6791	5074
6563	6805	5092
6593	6818	5095
6599	6862	5097
6602	6863	5103
6624	6867	5104
6628	6873	5135
6629	6883	5259
6697	6904	5320
6713	6914	5347
6730	6943	5348
6746	6959	5354
6783	6994	5365
6803	7010	5368
6804	7017	5373
6867	7052	5395
6883	7063	5437
6915	7071	5454
6942	7084	5471
6961	7119	5511
7009	7122	5517
7018	7142	5597
7063	7223	5599
7084	7236	5608
7102	7276	5617
7125	7308	5632
7156	7318	5639
7193	7325	5648
7198	7336	5657
7232	7344	5679
7261	7345	5683
7266	7354	5685
7275	7366	5719
7276	7370	5755
7308	7371	5765
7328	7378	5780
7342	7425	5789

7345	7445	5842
7354	7448	5844
7366	7467	5905
7378	7474	5952
7381	7479	5971
7391	7482	5991
7433	7485	6000
7445	7511	6041
7448	7513	6067
7471	7531	6114
7479	7573	6133
7485	7575	6149
7511	7594	6151
7571	7600	6180
7573	7604	6198
7591	7609	6202
7600	7638	6214
7604	7641	6223
7638	7649	6300
7667	7650	6307
7675	7690	6363
7711	7740	6392
7756	7756	6413
7758	7782	6415
7792	7784	6473
7804	7802	6523
7807	7804	6570
7812	7807	6585
7813	7816	6599
7816	7855	6602
7857	7857	6613
7871	7871	6617
7890	7890	6727
7905	7905	6741
7927	7918	6742
7941	7941	6743
7962	7970	6757
7972	7982	6763
7985	7995	6764
8011	8003	6775
8025	8025	6802
8040	8044	6873
8044	8062	6876
8087	8087	6899
8099	8095	6911
8102	8102	6914

8110	8105	6915
8146	8145	6938
8151	8151	6956
8167	8164	7009
8187	8206	7015
8206	8260	7018
8218	8299	7028
8225	8303	7052
8238	8314	7063
8247	8321	7078
8299	8337	7080
8309	8348	7122
8323	8361	7147
8337	8420	7152
8348	8424	7193
8356	8499	7211
8383	8516	7223
8406	8519	7232
8433	8533	7266
8435	8547	7275
8449	8569	7276
8498	8578	7292
8516	8600	7335
8547	8618	7342
8551	8620	7354
8555	8625	7378
8578	8647	7391
8589	8655	7393
8618	8657	7403
8620	8660	7444
8626	8671	7445
8660	8673	7454
8670	8694	7513
8692	8697	7531
8694	8753	7535
8697	8759	7569
8753	8761	7606
8759	8800	7619
8794	8801	7650
8808	8812	7660
8812	8818	7667
8818	8829	7691
8858	8845	7731
8872	8847	7734
8873	8849	7758
8887	8851	7767

8918	8854	7776
8926	8855	7792
8951	8858	7811
8957	8872	7813
8964	8873	7905
8975	8887	7912
8980	8916	7941
8993	8926	7946
8998	8934	7970
9001	8935	7972
9015	8951	7986
9022	8964	7991
9051	8993	8025
9069	8996	8063
9087	8998	8065
9132	9022	8084
9137	9025	8087
9142	9051	8095
9170	9082	8137
9200	9087	8140
9216	9099	8146
9232	9109	8150
9234	9142	8151
9244	9200	8187
9252	9244	8206
9264	9252	8218
9283	9283	8225
9341	9304	8247
9372	9321	8253
9446	9404	8289
9452	9414	8293
9460	9426	8294
9501	9440	8324
9521	9452	8325
9528	9454	8348
9531	9457	8352
9533	9486	8356
9551	9487	8366
9564	9489	8406
9567	9496	8420
9581	9498	8422
9593	9503	8424
9594	9515	8425
9605	9528	8498
9640	9531	8506
9670	9533	8515

9690	9564	8516
9719	9571	8544
9757	9581	8547
9775	9592	8578
9791	9594	8589
9809	9605	8594
9810	9606	8605
9821	9619	8610
9863	9625	8618
9905	9639	8619
9912	9640	8634
9958	9658	8655
9966	9677	8656
9981	9702	8658
9990	9716	8660
9996	9718	8684
10002	9723	8692
10012	9791	8697
10045	9795	8753
10068	9821	8759
10084	9845	8768
10106	9872	8794
10127	9881	8801
10194	9898	8812
10293	9923	8845
10349	9931	8852
10371	9990	8875
10374	10002	8878
10384	10007	8951
10387	10020	8972
10395	10051	8974
10427	10237	9041
10444	10240	9054
10519	10242	9069
10533	10247	9078
10586	10253	9101
10591	10264	9125
10602	10270	9128
10609	10293	9155
10724	10353	9199
10738	10365	9202
10763	10375	9214
10782	10384	9222
10806	10395	9232
10827	10405	9234
10828	10441	9241

10833	10444	9320
10834	10531	9341
10849	10542	9344
10888	10549	9350
10910	10565	9408
10918	10580	9446
11024	10596	9460
11053	10602	9486
11085	10608	9503
11116	10642	9531
11140	10656	9593
11141	10672	9594
11177	10677	9605
11211	10691	9674
11236	10695	9705
11244	10711	9716
11255	10757	9718
11289	10782	9719
11312	10827	9736
11391	10828	9757
11416	10833	9769
11446	10849	9777
11457	10869	9798
11493	10893	9801
11503	10898	9808
11727	10918	9809
11749	10942	9814
11808	10954	9822
11812	10988	9841
11883	11039	9845
11922	11085	9863
11923	11115	9880
12094	11116	9898
12112	11141	9981
12115	11177	9990
12156	11184	10014
12224	11203	10034
12231	11242	10045
12234	11244	10056
12250	11255	10076
12267	11284	10116
12294	11312	10127
12313	11331	10167
12375	11334	10194
12474	11349	10219
12492	11391	10253

12510	11416	10278
12517	11444	10293
12519	11459	10297
12568	11463	10358
12624	11502	10359
12640	11503	10365
12663	11526	10394
12664	11537	10424
12666	11577	10452
12775	11590	10468
12780	11600	10471
12827	11607	10542
12885	11715	10591
12901	11720	10609
12902	11727	10625
13010	11731	10656
13017	11744	10669
13074	11749	10691
13181	11765	10711
13452	11790	10741
13542	11812	10776
13593	11838	10809
13608	11854	10827
13667	11863	10828
13855	11883	10840
13875	11899	10842
13886	11922	10853
13913	11961	10869
14026	11979	10898
14166	11980	10932
14260	12049	10988
14405	12060	11058
15759	12084	11104
15765	12095	11116
1	12098	11189
3	12112	11207
9	12130	11235
11	12156	11241
38	12170	11244
62	12195	11255
74	12196	11312
113	12241	11356
115	12250	11371
117	12276	11408
118	12309	11444
127	12310	11463

202	12370	11511
248	12402	11588
277	12407	11590
311	12447	11610
323	12474	11624
331	12481	11638
337	12492	11722
379	12498	11727
427	12519	11802
428	12529	11812
446	12584	11842
449	12604	11859
459	12784	11863
464	12792	11905
465	12806	11914
470	12878	11922
552	12886	11965
555	13009	11982
572	13069	11987
603	13074	12006
605	13147	12008
674	13216	12072
676	13223	12095
697	13337	12098
710	13342	12142
749	13419	12186
779	13452	12195
789	13461	12241
792	13580	12250
854	13610	12257
870	13701	12313
880	13736	12373
896	13793	12375
897	13834	12481
921	13868	12519
976	13875	12529
984	14008	12584
1058	14026	12590
1065	14039	12609
1086	14067	12664
1111	14192	12759
1115	14211	12773
1135	14213	12878
1139	14243	12918
1149	14279	13074
1166	14290	13222

1185	14371	13342
1293	14390	13422
1320	14392	13452
1418	14477	13483
1433	14584	13542
1434	15073	13575
1454	15243	13593
1460	15501	13875
1469	15505	14026
1483	15506	14028
1501	15529	14050
1505	15532	14087
1518	15553	14112
1559	15555	14188
1561	15561	14208
1632	15597	14211
1659	15621	14279
1679	15626	14371
1688	15648	14584
1721	15660	14592
1723	1	14788
1778	6	16109
1882	11	16373
1907	14	16384
1990	38	16396
2140	60	16407
2147	62	16409
2156	112	16413
2166	139	16415
2171	147	16420
2193	197	16423
2231	210	16426
2331	277	16431
2358	311	16436
2383	361	16442
2384	414	16443
2420	427	16445
2423	428	16447
2441	465	16450
2460	468	16452
2495	470	16460
2499	472	16464
2507	473	16467
2542	545	16474
2594	590	16478
2683	610	16521

2709	691	16522
2742	697	16528
2812	700	16546
2882	749	16554
2888	791	16556
2924	808	6
2929	838	10
2942	849	65
2972	855	67
3028	931	70
3034	932	72
3147	938	94
3183	965	124
3304	967	127
3321	976	128
3329	1038	151
3347	1066	197
3356	1072	230
3395	1078	241
3410	1089	311
3415	1093	337
3434	1097	372
3456	1111	441
3466	1115	464
3558	1119	468
3655	1135	547
3699	1141	604
3747	1172	605
3749	1185	657
3814	1236	659
3824	1238	674
3825	1293	702
3841	1342	720
3905	1418	725
3969	1434	736
3970	1437	767
3991	1444	772
4110	1461	779
4239	1469	792
4252	1487	793
4266	1491	838
4313	1493	841
4333	1503	897
4362	1527	899
4398	1563	931
4467	1574	965

4477	1577	984
4497	1584	1000
4523	1593	1019
4569	1676	1044
4573	1683	1058
4604	1688	1059
4612	1696	1060
4631	1709	1067
4647	1713	1072
4710	1726	1088
4787	1731	1090
4797	1737	1135
4831	1821	1139
4926	1830	1142
4930	1849	1146
4971	1854	1150
4977	1882	1202
5049	2009	1350
5057	2140	1381
5098	2148	1409
5238	2155	1433
5244	2159	1460
5319	2172	1469
5330	2190	1474
5371	2211	1484
5381	2235	1487
5382	2296	1544
5412	2304	1574
5449	2331	1625
5527	2378	1669
5546	2432	1679
5548	2491	1691
5550	2499	1709
5617	2507	1713
5632	2591	1737
5649	2621	1822
5679	2627	1828
5727	2672	1835
5737	2684	1849
5755	2687	1962
5822	2763	1990
5842	2846	1998
5919	2924	2140
5938	2925	2142
5958	2931	2155
6000	2942	2156

6051	2950	2182
6082	2956	2185
6085	3032	2212
6427	3058	2235
6429	3069	2339
6437	3094	2441
6528	3140	2462
6589	3146	2499
6626	3161	2507
6633	3164	2578
6706	3177	2594
6723	3236	2685
6741	3240	2834
6743	3242	2867
6802	3247	2888
6805	3294	2914
6810	3308	2978
6818	3329	3043
6831	3331	3075
6860	3339	3159
6885	3361	3161
6899	3395	3290
6904	3417	3294
6953	3419	3329
6956	3482	3339
6963	3544	3380
7023	3558	3401
7095	3666	3452
7139	3679	3471
7142	3685	3503
7169	3695	3532
7223	3728	3592
7292	3731	3648
7337	3736	3660
7393	3749	3728
7414	3799	3747
7569	3803	3751
7619	3814	3764
7665	3824	3781
7683	3905	3814
7731	3956	3862
7769	3969	3911
7776	3970	3970
7811	3991	3978
7902	4027	3989
7907	4035	4009

7909	4044	4022
7912	4055	4027
7933	4092	4042
7957	4125	4125
7976	4151	4252
7989	4204	4253
8007	4398	4313
8084	4408	4319
8137	4455	4331
8142	4461	4480
8150	4480	4500
8155	4497	4529
8293	4513	4537
8294	4529	4573
8321	4532	4616
8358	4536	4695
8361	4569	4753
8400	4599	4766
8410	4631	4774
8424	4647	4797
8425	4764	4806
8503	4765	4893
8530	4766	4923
8600	4817	4926
8610	4823	4971
8619	4831	4989
8634	4923	5253
8647	4926	5286
8704	4971	5303
8813	4977	5319
8851	5049	5351
8852	5070	5363
8875	5097	5376
8880	5245	5449
8891	5250	5450
9058	5286	5500
9101	5287	5539
9184	5294	5603
9190	5331	5649
9282	5337	5727
9352	5338	5766
9356	5376	5784
9397	5378	5790
9439	5384	5940
9571	5410	5970
9618	5434	5977

9619	5437	6028
9656	5490	6085
9658	5527	6272
9662	5578	6346
9666	5611	6414
9716	5617	6437
9723	5648	6451
9753	5654	6626
9795	5679	6691
9822	5683	6716
9872	5714	6723
9926	5747	6733
9930	5765	6770
10056	5797	6831
10076	5815	6860
10116	5821	6967
10123	5911	7023
10164	5945	7034
10236	5975	7047
10242	6022	7065
10310	6067	7139
10320	6122	7160
10347	6180	7261
10357	6300	7308
10364	6307	7362
10365	6317	7370
10394	6354	7374
10424	6362	7396
10452	6406	7401
10471	6437	7434
10482	6458	7683
10553	6506	7766
10642	6528	7787
10703	6545	7810
10711	6594	7908
10821	6624	7911
10840	6626	7920
10842	6628	7933
10852	6696	7989
10853	6713	7994
10896	6716	8102
10932	6723	8217
10972	6727	8267
10991	6733	8328
11115	6743	8329
11181	6777	8340

11228	6802	8410
11371	6831	8499
11408	6892	8503
11610	6915	8533
11612	6967	8647
11731	7139	8688
11859	7169	8734
12003	7193	8763
12008	7198	8813
12095	7211	8860
12142	7232	8900
12498	7266	8934
12584	7275	8941
12918	7287	9074
13009	7335	9126
13222	7360	9240
13482	7362	9335
13710	7374	9513
13767	7391	9536
13834	7393	9649
14480	7471	9650
14754	7484	9658
14956	7526	9662
7	7535	9763
34	7576	9872
51	7665	9881
70	7667	9937
81	7675	10013
94	7691	10250
102	7718	10310
103	7731	10364
123	7758	10395
128	7767	10457
141	7792	10533
208	7911	10602
230	7927	10763
241	7957	10800
264	7976	10848
297	7985	10893
320	7986	10895
349	8007	10922
436	8084	11002
462	8133	11150
547	8137	11177
604	8140	11178
677	8146	11181

719	8155	11228
720	8218	11236
725	8247	11261
735	8302	11299
737	8340	11446
742	8356	11457
767	8383	11720
772	8410	11808
793	8498	12447
865	8506	12452
883	8551	12504
1038	8555	12640
1059	8589	12645
1060	8594	12780
1082	8599	13650
1100	8610	13710
1114	8626	14166
1124	8688	14401
1146	8692	14943
1190	8704	15347
1200	8729	15365
1202	8852	15635
1409	8974	15669
1522	8975	16240
1528	9058	16378
1669	9086	16394
1691	9090	16399
1737	9148	16421
1770	9202	16479
1821	9214	16484
1822	9232	8
1828	9264	34
1835	9302	51
1849	9341	98
1861	9352	147
1905	9439	192
1914	9446	202
1959	9450	228
2067	9462	343
2119	9471	436
2191	9595	470
2214	9662	513
2235	9690	532
2367	9740	552
2405	9810	650
2410	9814	676

2491	9841	677
2498	9905	715
2534	9926	727
2535	9930	749
2569	9958	808
2578	9981	821
2884	10013	849
2909	10045	854
2911	10074	870
2925	10127	896
2938	10164	933
3043	10167	1029
3046	10236	1077
3137	10266	1085
3146	10310	1086
3148	10359	1133
3164	10387	1144
3192	10394	1149
3222	10452	1172
3334	10468	1177
3349	10482	1278
3361	10586	1281
3380	10609	1464
3401	10703	1522
3402	10724	1551
3471	10741	1599
3503	10763	1606
3575	10834	1690
3643	10932	1694
3781	10991	1729
3858	11104	1836
3862	11240	1905
3882	11241	1907
3928	11289	1972
3947	11299	2067
4300	11305	2119
4306	11371	2122
4319	11446	2139
4410	11525	2147
4479	11610	2197
4500	11638	2357
4513	11687	2367
4537	11808	2458
4616	11810	2569
4776	11842	2621
4803	11859	2674

4916	12094	2682
5018	12115	2724
5028	12142	2807
5033	12186	2844
5092	12234	2929
5303	12268	2936
5337	12313	2938
5338	12373	2950
5348	12375	3046
5363	12424	3074
5395	12508	3098
5450	12568	3146
5458	12664	3148
5580	12666	3237
5597	12727	3276
5625	12759	3358
5790	12780	3402
5885	12893	3514
5948	12906	3581
6022	12918	3643
6198	13111	3790
6346	13222	3947
6375	13298	4055
6414	13453	4096
6416	13542	4131
6458	13667	4398
6570	13710	4427
6722	13734	4461
6733	13767	4514
6892	14028	4525
7034	14036	4528
7048	14187	4564
7086	14799	4569
7104	15137	4776
7160	15425	4803
7335	15510	4962
7360	15559	5018
7362	15562	5371
7401	15587	5496
7434	15591	5580
7535	67	5961
7650	70	6022
7710	98	6064
7787	102	6070
7908	103	6099
7911	127	6255

7920	128	6305
7949	202	6309
8098	260	6331
8224	269	6354
8267	343	6355
8289	349	6362
8303	365	6416
8422	372	6427
8499	375	6429
8599	379	6693
8651	395	6886
8656	421	6972
8669	442	7086
8688	446	7279
8768	449	7287
8860	459	7373
8941	530	7414
9202	552	7656
9214	555	7844
9335	572	8040
9489	603	8080
9650	657	8098
9705	662	8110
9798	663	8142
9898	674	8203
10013	677	8400
10034	719	8537
10250	721	8637
10261	727	8651
10340	780	8704
10468	789	8879
10544	793	8880
10656	853	9282
10825	896	9318
11011	897	9356
11511	899	9378
11559	921	9439
12170	953	9533
12548	981	9791
12645	984	10041
12759	1000	10089
13450	1044	10164
14015	1051	10340
14	1058	10357
29	1065	10427
92	1067	10472

114	1077	10480
125	1090	10703
133	1102	10910
147	1114	11242
187	1139	11559
204	1168	11612
225	1433	12003
229	1454	12058
235	1499	12130
247	1518	12267
468	1614	13737
513	1625	13834
575	1659	13855
663	1664	15324
933	1675	16375
1044	1712	16395
1078	1797	16412
1085	1813	16439
1088	1822	16462
1144	1836	16519
1171	1846	9
1172	1856	23
1464	1959	81
1484	2044	101
1499	2064	115
1606	2124	120
1708	2147	149
1809	2160	172
1972	2225	208
1998	2229	209
2022	2299	233
2077	2358	375
2125	2405	495
2160	2410	563
2195	2423	575
2197	2441	735
2357	2572	766
2422	2736	774
2424	2827	788
2611	2884	883
2807	2888	1021
2834	2972	1065
2844	3034	1078
2879	3182	1100
3062	3253	1166
3074	3401	1269

3120	3434	1272
3290	3471	1273
3346	3514	1505
3412	3550	1525
3514	3575	1580
3581	3657	1632
3648	3660	1674
3660	3699	1738
3715	3741	1914
3764	3777	1974
3774	3781	2121
3903	3802	2171
3978	3825	2295
3989	3862	2498
4055	3980	2561
4431	4022	2729
4695	4064	2736
4764	4073	2749
4955	4102	2879
5261	4131	2965
5599	4253	3034
5784	4313	3071
5789	4319	3162
5926	4331	3222
6113	4456	3334
6202	4479	3347
6266	4525	3453
6305	4573	3483
6331	4596	3485
6369	4820	3549
6705	4870	3639
6886	4965	3654
7022	5058	3722
7100	5092	3774
7152	5105	3858
7208	5253	3882
7279	5303	4099
7396	5348	4135
7403	5351	4139
7444	5371	4300
7546	5395	4306
7780	5425	4546
7991	5495	5057
8057	5496	5098
8062	5500	5244
8253	5597	5250

8328	5603	5550
8340	5632	5625
8427	5649	5653
8956	5719	5655
8972	5755	5730
9126	5905	5885
9318	5931	5948
9344	5938	5958
9378	5958	5996
9471	6000	6244
9552	6064	6245
9589	6082	6266
9801	6113	6369
10457	6114	6426
10479	6198	6682
10895	6202	6683
12058	6219	6783
12203	6414	6818
13467	6424	6837
10	6474	6963
63	6481	7012
65	6570	7022
68	6585	7048
116	6602	7123
124	6674	7337
184	6860	7453
192	6885	7514
233	6956	7872
363	6972	7957
372	7004	8224
375	7009	8297
507	7023	8494
518	7086	8789
563	7125	8883
595	7433	8956
650	7444	10020
774	7454	10236
788	7606	10374
851	7619	10642
906	7741	10806
1021	7776	10825
1151	7811	11001
1177	7920	11005
1201	7933	11232
1245	7972	15300
1599	8011	16393

1836	8040	16410
2064	8098	16419
2126	8099	16424
2142	8110	7
2780	8142	26
2867	8221	29
2978	8225	44
3071	8253	63
3162	8267	92
3237	8289	150
3502	8293	160
3654	8294	189
3790	8323	225
4133	8406	445
4139	8422	474
4248	8425	507
4403	8503	518
4406	8530	851
4564	8539	865
4962	8619	906
4989	8651	1082
5117	8768	1245
5653	8875	1558
5655	8894	1608
5996	8943	1700
6183	8972	1715
6255	9101	1809
6309	9125	1937
6426	9126	2146
6500	9282	2364
7324	9335	2444
7453	9344	2521
7454	9378	2836
7844	9397	3062
8297	9650	3178
8552	9704	3412
8789	9705	3502
9074	9719	3657
9513	9798	3888
9536	9801	3903
9763	9809	4029
10089	9822	4403
10472	10034	4450
10480	10056	4911
11982	10116	5117
23	10199	5425

168	10340	5646
189	10347	5898
267	10357	5959
476	10364	5960
532	10427	6113
571	10471	6183
848	10553	6594
1525	10591	7310
1694	10646	7324
1715	10738	7730
2092	10825	7780
2546	10842	8036
2965	10853	8099
3452	10922	8552
3675	11228	10482
4029	11457	11011
4349	11511	11170
4429	11530	12548
5258	12008	14243
5801	12267	16397
5910	12640	35
6837	12645	108
8494	14166	114
8641	15300	123
8879	15508	167
8883	15577	168
9254	68	229
9434	81	267
9623	94	302
136	117	330
144	204	363
160	228	476
304	274	752
715	297	966
740	306	1095
787	331	1395
1527	337	1416
1579	450	1517
1608	464	2125
1871	495	2191
2121	604	2717
2170	605	2723
2222	660	2760
2444	676	3346
2561	702	3444
2804	725	3588

3010	742	3620
3245	779	3739
3308	870	3900
3343	883	4248
3358	1086	4349
3483	1088	4553
3595	1151	4654
3793	1202	4955
4135	1409	5258
4450	1464	5676
4488	1501	6096
4553	1505	6196
4614	1516	6226
4709	1551	6312
4911	1679	7710
5407	1694	7984
5676	1809	8839
5706	1812	9254
5961	1905	9623
6031	1907	10182
6245	2139	10215
6272	2156	13459
6385	2197	16398
6683	2422	16416
7730	2463	140
7872	2495	142
7881	2578	303
9508	2594	571
9510	2682	740
10269	2777	1519
26	2844	1871
44	2867	2130
120	2879	2170
171	2929	2184
172	2938	2195
330	3137	2780
730	3159	3242
966	3290	3254
969	3347	3375
1029	3356	3782
1063	3410	3793
1143	3503	3928
1145	3559	4133
1415	3581	4380
1416	3643	4406
1622	3670	4488

1837	3764	4604
1974	3766	4614
2184	3774	4937
2295	3790	5899
2579	3947	6500
3409	3978	7546
3453	4306	7549
3485	4358	7817
3588	4410	9220
3739	4500	16386
4636	4514	184
4899	4537	299
4937	4610	304
5085	4612	1604
6099	4695	1622
6196	4774	1705
6312	4776	2022
7514	4787	2244
8203	4797	2875
8763	4803	3039
8839	5033	3349
9739	5083	3575
9852	5319	4508
35	5363	4636
386	5539	4916
445	5784	5382
837	5789	5706
1414	5790	6927
1604	5842	11867
1714	5885	12302
2365	6085	16400
2382	6309	16408
2428	6392	136
2836	6416	171
2875	6427	235
3782	6679	692
5311	6722	787
5751	6837	1145
6927	6886	1414
7549	6899	1415
167	6989	1508
385	7018	1515
494	7048	1819
745	7080	2239
1002	7123	2546
1515	7261	2709

1517	7381	2964
1519	7401	3204
1937	7434	3245
2018	7569	3343
2019	7908	5490
2146	7912	5729
2646	7949	7881
2717	7989	8641
3178	8055	46
3444	8150	232
3900	8297	386
5960	8427	494
6096	8734	947
8352	8789	1057
80	8813	1063
110	8860	1514
142	9190	2447
213	9234	2461
232	9318	2721
303	9356	2746
474	9508	2804
1508	9536	3595
1922	9593	3596
2721	9757	5012
2733	9763	5751
3254	9953	8061
3596	10076	9739
3620	10159	116
3888	10194	144
3990	10250	213
6654	10424	595
7817	10479	649
8036	10533	837
46	10806	1002
61	10840	1714
79	11178	1922
129	11261	2365
140	11559	2380
158	11891	2579
250	11897	3018
393	12003	3279
1057	12935	3922
1514	10	4041
2118	34	5801
3018	51	7631
3375	65	8527

4434	92	9852
7589	133	11891
5	187	80
108	209	255
692	230	385
948	241	480
1053	334	508
1887	436	679
2226	720	706
2239	736	707
2307	772	1143
2380	774	2118
2436	1021	2382
2476	1059	2428
3320	1060	3409
3571	1100	3447
5012	1144	3571
5868	1146	3955
7631	1149	4142
8527	1166	4634
24	1177	4709
441	1395	4957
707	1522	5567
766	1669	16389
1819	1690	16392
2236	1691	5
2461	1828	24
2603	1972	61
3107	2125	79
4508	2367	737
5567	2420	2236
524	2424	3809
1252	2569	4434
1417	2834	5013
2130	3043	5868
2447	3046	6394
2462	3192	6508
2964	3199	738
3884	3219	916
4634	3222	1417
7858	3402	1814
373	3452	3884
1449	3648	4050
2189	3654	5311
2736	3990	6689
3279	4431	7858

4957	4564	137
6508	4893	404
150	4954	848
480	5018	1137
706	5580	2307
1047	5599	2436
1126	5655	10929
1137	5766	16377
2518	5948	129
3447	5996	969
3922	6183	2226
7926	6255	2
8117	6305	524
135	6480	1126
137	6770	1887
1738	6963	3543
3543	7034	3632
4050	7160	6687
5013	7310	3099
6044	7337	6690
1134	7414	158
6394	7453	342
382	7787	2476
404	7962	2520
1814	8061	8117
3955	8366	8567
4142	8656	13649
2	8879	382
2520	8880	1449
4423	8941	2189
4515	8956	135
214	9074	1180
508	9222	2747
5918	9552	373
249	10114	10523
1558	10480	745
3809	10895	2518
6034	11982	4515
6687	12058	948
7924	13269	1134
391	13593	1053
649	14672	4423
2745	15514	16374
2533	15539	9239
1210	7	133
313	63	1047

1627	151	3010
957	189	297
252	507	2759
278	513	2366
292	547	1277
1173	563	6032
	788	2742
	854	6039
	865	2733
	933	6036
	1029	1276
	1085	
	1239	
	1460	
	1484	
	1525	
	1606	
	1715	
	1914	
	1998	
	2018	
	2119	
	2171	
	2195	
	2357	
	2364	
	2521	
	2546	
	2978	
	3074	
	3162	
	3415	
	3882	
	4139	
	4248	
	4252	
	4300	
	4309	
	4349	
	4406	
	4429	
	4450	
	4604	
	4687	
	4989	
	5028	

5057
5098
5117
5412
5646
5706
6369
6426
7022
7152
7279
7292
7324
7396
7403
7546
7710
7844
8203
8449
8552
8883
9513
10261
11011
11612
15511
15526
15557
44
192
225
328
341
441
532
715
851
1133
1233
1245
1416
1608
1632
1700
1974

2067
2126
2193
2444
2498
2729
2784
2807
2965
3320
3349
3483
3502
3793
3858
3903
4029
4133
4135
4207
4911
4962
5244
5625
5751
6479
7715
7748
7780
7817
8224
8400
8763
9623
10089
10472
10910
11181
11902
15579
124
160
235
330
363
476

575
730
738
766
906
1235
1599
2121
2170
2191
2709
3062
3148
3334
3346
3358
3485
3888
3928
4553
4616
4636
4709
4916
4937
5382
5550
5676
6331
6927
7683
7881
8036
8494
8699
9254
10269
15516
26
29
184
233
267
571
767
1095

1559
1843
2146
2295
2721
2723
2836
3071
3453
3588
3739
4488
4705
5258
5653
5926
6196
6245
6272
6349
7730
11055
12548
12653
15518
15522
23
79
149
172
229
518
595
648
787
848
969
1063
1519
1622
1787
2184
2603
2804
3132
3620

3782
3900
4955
5567
5868
5896
7514
7549
7872
8352
8641
8839
9739
10374
120
167
304
318
323
445
650
735
745
1082
1143
1234
1515
1708
1871
1922
1937
2717
2875
3245
3254
3412
3444
3595
4403
5801
6096
6312
15525
15535
123
141

232
524
786
1002
1324
1414
1604
2022
2447
2722
4614
7592
15507
9
80
114
150
386
474
1627
1819
2380
3375
6266
6500
15499
35
61
101
168
1415
1517
2118
2365
2382
2462
2561
2750
3596
4508
5960
6783
110
142
208
707

737
740
837
966
1561
1714
2239
2428
3447
3557
5012
7100
9852
303
692
1145
1508
1514
1738
1887
2142
2436
2579
3409
5013
6032
24
46
373
385
1417
2226
2733
2964
3018
3178
3279
3343
7589
140
171
706
1057
2461
3714
4099

4434
5788
6683
7631
8117
494
2130
2476
4957
948
3884
4634
5311
6508
116
261
1814
2236
3571
5
108
129
135
144
382
1053
480
1047
1126
2307
3955
137
3010
3922
4142
6031
252
1137
1449
3809
6654
136
158
3720
4050
8328

2
508
649
1744
2189
292
752
1134
2033
6687
7858
4423
6394
6034
2518
3543
4515
8527
624
2520
10239
213
313
6035
2742
6044
404
5961
332
1558
1190
1952
2776
6099
214
2366
2745
2774
278
115
1230
1231
248
6473
1171

16
